# *SLC2A9* rs1014290 Polymorphism is Associated with Prediabetes and Type 2 Diabetes

**DOI:** 10.1155/2022/4947684

**Published:** 2022-12-12

**Authors:** Xuemei Zhang, Guangsen Hou, Fang Li, Xiao Zheng, Qian Nie, Guangyao Song

**Affiliations:** ^1^Department of Rheumatism and Immunology, Hebei General Hospital, 348 Heping West Road, Shijiazhuang, Hebei 050000, China; ^2^Department of Geriatric, Affiliated Hospital of Hebei Engineering University, 81 Congtai Road, Handan, Hebei 056000, China; ^3^Physical Examination Center, Hebei General Hospital, 348 Heping West Road, Shijiazhuang, Hebei 050000, China; ^4^Hebei Key Laboratory of Metabolic Diseases, Hebei General Hospital, 348 Heping West Road, Shijiazhuang, Hebei 050000, China

## Abstract

**Purpose:**

To investigate the association of the A/G rs1014290 polymorphism in *SLC2A9* with type 2 diabetes (T2DM) and prediabetes mellitus (pre-DM). *Patients and Methods*. We enrolled 1058 patients who attended the Hebei General Hospital, Shijiazhuang, Hebei Province, China. The patients underwent general testing and oral glucose tolerance tests and were divided into three groups: 352 patients newly diagnosed with T2DM, 358 patients with pre-DM, and 348 healthy controls. The single nucleotide polymorphism (SNP) was detected by ligase detection reactions. The *χ*^2^ test, one-way ANOVA, and binary logistic regression analysis were used to analyze the results.

**Results:**

In the T2DM group, the GG genotype frequency at the rs1014290 locus was significantly lower (14.8%) than it was in the healthy controls. Furthermore, the GG genotype group was associated with a reduced risk of T2DM in unadjusted and confounder-adjusted models compared with the risk in the AA genotype group. The G allele in the *SLC2A9* rs1014290 locus decreased susceptibility to T2DM. In the pre-DM group, the GG and AG genotype groups had no significant correlation with the risk of pre-DM in any of the models. In the T2DM group, the uric acid level was significantly lower in the GG genotype group. In the T2DM and pre-DM groups, the HOMA-*β* levels were significantly higher in the GA (*P*  < 0.001) and GG (*P*  < 0.001) genotype groups than it was in the AA genotype group, and HOMA-IR was significantly lower in the GA (*P*  < 0.001) and GG (*P*  < 0.001) genotype groups than it was in the AA genotype group.

**Conclusion:**

The A/G (rs1014290) SNP in *SLC2A9* is closely related to the occurrence and development of diabetes.

## 1. Introduction

Type 2 diabetes (T2DM) is a complex disease characterized by insulin resistance and a relative lack of insulin. Its etiology is related to heredity and the environment. The association between genetic polymorphisms and type 2 diabetes mellitus has been confirmed by many studies. The study of Aka et al. in 2021 shows that KCNJ11rs5219, SLC30A8 rs13266634, and HHEXrs1111875 polymorphisms are associated with T2DM [1]. The prevalence of T2DM and prediabetes (pre-DM) in the Chinese adult population were reported to be 11.6% and 50.1%, respectively [2]. Pre-DM is an early warning signal of T2DM. If early detection and early intervention do not occur, pre-DM will progress to T2DM, leading to delays in diagnosis and treatment.

Glucose transporter 9 (GLUT9), encoded by the solute carrier *SLC2A9*, can transport glucose, fructose, and/or uric acid [3, 4]. The expression and functions of GLUT9 are tissue-selective. The abundance of GLUT9 is significantly increased in the liver and kidney tissue of T2DM mice [5]. In pancreatic beta cells, GLUT9 was found to be related to insulin secretion after glucose stimulation [6]. GLUT9 is also a high-throughput uric acid transporter [7–10], and serum uric acid is associated with insulin resistance and is considered an independent risk factor for T2DM [11–14]. Despite these findings, the relationship between GLUT9 and diabetes is still unclear, and the specific mechanism needs to be further explored. Therefore, we investigated whether the *SLC2A9* rs1014290 polymorphism was associated with T2DM and pre-DM.

Many studies have shown that *SLC2A9* has >60 variant sites [15]. These studies explored mainly the correlation between single nucleotide polymorphisms (SNPs) in *SLC2A9* and hyperuricemia and gout [16, 17], but a few of them focused on T2DM. One study in a Taiwan Han population showed that SNP rs1014290 in the third intron of *SLC2A9* was associated with T2DM [18]. The wild type of this gene locus is AA, the homozygous mutant is GG, and the heterozygous mutant is AG. The GG genotype of rs1014290 can reduce the risk of T2DM, and therefore the correlation between *SLC2A9* SNPs and glucose metabolism disorders is of interest. Most of the current studies have focused on the relationship between *SLC2A9* SNPs and T2DM and not on pre-DM, especially in the Han population. In this study, descendants of Han nationality in Hebei Province, China, with T2DM and pre-DM were selected as the study population. The correlation between rs1014290 and T2DM and pre-DM was investigated by identifying rs1014290 variants.

## 2. Materials and Methods

### 2.1. Study Subjects

We enrolled 1058 patients who were outpatients or inpatients at the Endocrinology Department of Hebei General Hospital, Shijiazhuang City, Hebei Province, China, from January to December 2018 in this study. Among them, there were 358 patients with pre-DM (165 males and 193 females), 352 patients with T2DM (159 males and 193 females) who were controlled by diet and exercise alone and did not receive diabetes medication, and 348 healthy individuals (145 males and 203 females) in the physical examination who were matched by age and sex. We use the 1999 World Health Organization's diagnostic and classification criteria for diabetes to determine [19]: pre-DM, 6.1 mmol/L ≤ fasting blood glucose <7.0 mmol/L, 7.8 mmol/L ≤ 2 h postprandial blood glucose (measured after taking 75 g glucose orally) < 11.1 mmol/L; T2DM: fasting blood glucose ≥7.0 mmol/L or 2 h postprandial blood glucose (measured after taking 75 g glucose orally) ≥ 11.1 mmol/L. Collect general information such as age, gender, body mass index, and blood pressure. The study obtained the consent of all subjects and was approved by the Ethics Committee of Hebei General Hospital. Subjects voluntarily signed an informed consent form.

### 2.2. Biochemical Analysis

We use an automatic biochemical analyzer to detect blood glucose at each time of the glucose tolerance test, uric acid (UA), and blood lipids (including TC, TG, LDL, and HDL) (Hitachi 7600–110, Japan). Detection of Glycated Hemoglobin (HbA1c) by Chromatographic Analysis (ADAMS A1c HA-8180 analyzer, Japan). Determination of serum insulin levels by electrochemiluminescence (Roche Cobas e601, Germany). The degree of insulin resistance was assessed by calculating beta cell function (HOMA-*β*) and the insulin resistance index (HOMA-IR), using the HOMA2 calculator version 2.2.3 (Oxford University Diabetes Trial Unit).

### 2.3. Blood Sampling and Genotyping

Genotyping and determination of gene polymorphisms were carried out with reference to the literature [20]. Genomic DNA extraction kit (Generay Biotechnology, Shanghai, China). Genotyping of patient SLC2A9 by DNA sequencing (ABI3730 genetic analyzer, Applied Biosystems, USA). The primers for gene amplification were as follows: 5'-GGATTCACAACTATCTTACTCAT-3' (forward); 5'-CAGGGTTATGTTCCATTTATCT-3' (reverse); designed using Primer Premier 5.0 (Premier Biosoft International, CA, USA). The samples were sequenced using GeneMarker v2.2.0 (PA, USA).

### 2.4. Statistical Analysis

The data were analyzed using SPSS 22.0 (IBM Corp., Armonk, NY, USA). Quantitative data conforming to a normal distribution were analyzed by one-way ANOVA. The Pearson's *χ*^2^ test was applied to verify whether each genotype conforms to Hardy–Weinberg equilibrium. The comparison of genotype and allele frequencies between groups was performed using a simple *χ*^2^ test, and the risk of T2DM and pre-DM among the genotype groups was assessed by binary logistic regression analysis. All *P* values were two-tailed, and *P* values <0.05 were considered significant.

## 3. Results

### 3.1. Comparison of the General Data between the Three Groups

Systolic blood pressure and BMI in the T2DM and pre-DM groups were significantly higher than those in the healthy control group (*P*  < 0.05), whereas UA, TC, TG, LDL, HbA1c, FPG, and PBG levels were significantly higher than those in the control group, and the differences were statistically significant (*P*  < 0.05). HOMA-IR in the T2DM group was higher than that in the control group, whereas there was no significant difference in HOMA-IR between the pre-DM and control groups. The levels of HDL, fasting serum insulin, and HOMA-*β* in the T2DM and pre-DM groups were significantly decreased compared with their levels in the control group (*P*  < 0.001). Comparisons of the general data and biochemical indexes for the three groups are provided in Table 1.

### 3.2. Comparison of the Genetic Correlation of the *SLC2A9* A/G (rs1014290) SNP in the Three Groups

The expression of the three genotypes of the *SLC2A9* rs1014290 SNP conforms to Hardy–Weinberg equilibrium. In the pre-DM group, the frequencies of the GA and GG genotypes were 50.3% and 17.6%, respectively, and the frequency of the G allele (42.7%) was lower than its frequency in the healthy controls, but the difference was not statistically significant. In the T2DM group, the frequencies of the GA and GG genotypes were 48.9% and 14.8%, respectively, and the frequency of the G allele (39.2%) was also significantly lower than its frequency in the controls. Analysis of the *SLC2A9* A/G comparisons, dominant ((GG + AG) vs. AA), additive (GG vs. AA), recessive (GG vs. (AG + AA)), and codominant ((AA + GG) vs. AG), showed that the recessive and additive models were both significantly correlated with T2DM (*P*=0.05, *P*=0.021). In the pre-DM group, no statistically significant difference was found for the dominant, additive, recessive, and codominant comparisons (*P*=0.520, *P*=0.316, *P*=0.342, and *P*=0.880 respectively). The results are shown in Table 2.

In the T2DM group, the GG genotype was associated with a reduced risk of T2DM in both the unadjusted and adjusted models. In the model that was not adjusted for confounding factors, the odds ratio (OR) was 0.595 (95% confidence index (CI) 0.383–0.925, *P*=0.021). In model 1, which was adjusted for age, sex, and BMI, the OR was 0.375 (95% CI 0.197–0.646, *P*=0.001). In model 2, which was adjusted for age, sex, BMI, and UA, the OR was 0.278 (95% CI 0.078–0.990, *P*=0.048). In model 3, which was adjusted for age, sex, BMI, UA, TC, TG, HDL, and LDL, the OR was 0.248 (95% CI 0.063–0.966, *P*=0.045). In the T2DM group, the association of the AG and AA genotypes with reduced risk was only significantly different in model 1 (OR = 0.484, 95% CI 0.265–0.884, *P*=0.018) (Table 3).

In the pre-DM group, the GG and AG genotypes had no significant correlation with the risk of pre-DM in the unadjusted and adjusted models (Table 4).

### 3.3. Correlation of *SLC2A9* rs1014290 with Different Clinicopathological Characteristics of T2DM and Pre-DM Groups

In the T2DM group, the levels of UA, FPG, PBG, and HbA1c in the GG genotype group were significantly decreased compared with their levels in the GA and AA genotype groups. The levels of TC and TG in the GG genotype group were significantly decreased compared with their levels in the AA genotype group. In the pre-DM group, the FPG level in the GG genotype group was significantly decreased compared with its levels in the AA and GA genotype groups, whereas the UA, TG, TC, and HbA1c levels were not significantly different among the three genotype groups. In both the T2DM and pre-DM groups, the HOMA-*β* levels in the GA (*P*  < 0.001) and GG (*P*  < 0.001) genotype groups were significantly higher than those in the AA genotype group. However, HOMA-IR was significantly lower in the GA (*P*  < 0.001) and GG (*P*  < 0.001) genotype groups compared with its level in the AA genotype group (Tables 5 and 6).

## 4. Discussion

The results of this study show that the serum UA level in the T2DM and pre-DM groups was significantly higher than it was in the healthy control group. Significant increases in TC, TG, LDL, HbA1c, FPG, and PBG levels were also found in the T2DM and pre-DM groups compared with their levels in the control group. The number of patients with the GG genotype was significantly lower in the T2DM group than it was in the control group. The risk of developing T2DM was significantly reduced for the GG genotype compared with the risk for the AA genotype, and this association persisted after adjusting for confounders of UA. The serum UA, TG, TC, FPG, PBG, and HbA1c levels in the GG genotype group were significantly decreased compared with their levels in the AA and GA genotype groups. These findings indicate the GG genotype was significantly associated with lower blood UA levels and a lower risk of T2DM compared with the other two genotypes.

GLUT9 [20] was once classified as a glucose and/or fructose transporter and, although its transport activity is very low, GLUT9 has a large effect on glucose metabolism [7, 21, 22]. *GLUT9* is upregulated in the liver and kidney tissues of diabetic mice and affects glucose-induced insulin secretion in pancreatic *β* cells [6, 23–25]. In two of these studies [6, 25], small interfering RNA knockdown of *GLUT9* in MIN6 and INS cells resulted in reduced cellular ATP levels that correlated with reductions in glucose-stimulated insulin secretion. These studies confirmed that *GLUT9* was expressed in murine and human *β*-cells and suggested that GLUT9 may participate in glucose sensing in *β*-cells. In previous studies, we also showed that *GLUT9* was upregulated in the renal tissues of hyper-lipid-induced insulin resistant mice, along with elevated serum UA, which can be ameliorated by resveratrol intervention [26].

More recently, GLUT9 has been identified as a UA transporter, as well as being a glucose and/or fructose transporter [7, 23, 27, 28]. Increased UA levels were found to be associated with the incidence of T2DM, and every 60 *μ*mol/L increase of UA was associated with a 1.17 times an increase in T2DM incidence [14, 28]. The incidence of T2DM in patients with hyperuricemia is about 35% [11]. UA affects the risk of diabetes by affecting glucose metabolism and insulin secretion of pancreatic *β*-cells [29, 30]. Other studies have shown that UA was closely correlated with FPG, fasting serum insulin, and PBG, and was positively proportional to HOMA-IR [31, 32]. The occurrence of diabetes and hyperuricemia is closely related to eating habits, but genetic background may be the internal source of the occurrence and development of T2DM and per-DM.

*SLC2A9* encodes GLUT9 [33], and SNPs in *SLC2A9* are not only associated with UA levels, but also with pancreatic *β*-cell function and diabetes [31, 34–36]. This association is influenced by region, ethnicity, and sex [7]. SNP rs1014290 is an A/G variant located in the third intron of *SLC2A9* [37, 38]. Previous studies have shown that the *SLC2A9* rs1014290 SNP was associated with the risk of diabetes, and that the GG genotype played a protective role in the occurrence of T2DM [18, 33, 39]; however, another study concluded that this protective role was not related to the level of blood UA [40]. Only a few studies have investigated the association between the rs1014290 SNP and T2DM and pre-DM, and no relevant studies have been conducted in northern China. In this study, a northern Chinese cohort was divided into T2DM and pre-DM groups. In the T2DM group, the GG genotype and G allele frequencies were significantly lower than they were in the healthy control group. In the pre-DM group, there were no significant differences in the GA and GG genotype and G allele frequencies compared with those in the control group. However, the FPG level was significantly decreased and the HOMA-*β* level was significantly higher in the GG genotype group than they were in the AA genotype group, and HOMA-IR was significantly lower than it was in the AA genotype group. The binary regression analysis showed that the risk of developing T2DM was significantly reduced in GG genotype group compared with the risk in the AA genotype group. Studies have shown that the G allele in *GLUT9* rs1014290 locus reduced susceptibility to T2DM.

This study has a number of limitations. (1) To avoid the influence of different regions on the results, all participants were from northern China. The pathogenesis of diabetes is complex and is known to be affected by heredity, environment. And dietary habits, but the role of environmental factors was not considered in this study. To confirm the effects of the *SLC2A9* A/G (rs1014290) SNP on diabetes, large-scale clinical data are needed. (2) *GLUT9* expression was not measured directly in this study, so the effects of the A/G (rs1014290) SNP on *GLUT9* expression and its impact on the onset and progression of pre-DM and T2DM needs further study. (3) *SLC2A9* has multiple SNP loci, but in this study only a single site rs1014290 in intron 3 was considered. Given the complexity and conjunction of gene regulation, further studies that include more SNP loci are needed to guide the diagnosis and treatment of clinical T2DM and pre-DM.

## 5. Conclusion

The *SLC2A9* A/G (rs1014290) SNP is closely related to the occurrence and development of pre-DM and T2DM. The G allele in the *SLC2A9* rs1014290 locus reduced the susceptibility to T2DM. Our findings need to be replicated in a larger group of patients with different ethnic backgrounds to confirm this relationship.

## Figures and Tables

**Table 1:** Comparison of the general data and biochemical parameters for the control, prediabetic, and type 2 diabetic groups.

Variables	Groups			Pairwise comparisons		
	Controls (*n* = 348)	Pre-DM (*n* = 358)	T2DM (*n* = 352)	All *P*	*P*1	*P*2
Age (years)	42.8 ± 13.05	52.56 ± 12.32	59.1 ± 10.23	<0.001	<0.001	<0.001
Male/female (*n*)	145/203	165/193	159/193	0.372	0.180	0.291
BMI (kg/m^2^)	25.09 ± 3.83	26.99 ± 3.84	26.89 ± 3.71	<0.001	<0.001	<0.001
SBP (mmHg)	124.03 ± 14.12	127.13 ± 13.39	131.42 ± 13.20	<0.001	0.002	<0.001
DBP (mmHg)	76.43 ± 9.52	77.14 ± 9.28	78.18 ± 8.86	0.041	0.306	0.012
UA (umol/L)	241.34 ± 45.8	319.76 ± 37.6	448.08 ± 73.41	<0.001	<0.001	<0.001
TG (mmol/L)	1.37 ± 0.98	1.76 ± 1.45	1.94 ± 1.98	<0.001	0.001	<0.001
TC (mmol/L)	4.60 ± 0.91	4.98 ± 0.96	5.07 ± 1.04	<0.001	<0.001	<0.001
HDL (mmol/L)	1.37 ± 0.32	1.27 ± 0.25	1.30 ± 0.31	<0.001	<0.001	0.014
LDL (mmol/L)	2.79 ± 0.71	3.08 ± 0.78	2.97 ± 0.85	<0.001	<0.001	0.007
FPG (mmol/L)	5.00 ± 0.36	5.76 ± 0.59	7.83 ± 1.84	<0.001	<0.001	<0.001
PBG (mmol/L)	5.47 ± 1.03	8.20 ± 1.33	12.71 ± 2.72	<0.001	<0.001	<0.001
FINS (*μ*U/mL)	9.91 ± 3.57	9.00 ± 3.22	7.80 ± 2.78	<0.001	0.001	<0.001
Postprandial FINS (*μ*U/mL)	44.10 ± 22.95	65.26 ± 22.13	47.64 ± 16.13	<0.001	0.001	0.023
HbA1c (%)	5.55 ± 0.34	5.77 ± 0.38	7.33 ± 1.51	<0.001	<0.001	<0.001
HOMA-IR	2.20 ± 0.82	2.31 ± 0.89	2.70 ± 1.13	<0.001	0.221	<0.001
HOMA-*β*	139.89 ± 67.95	85.55 ± 40.11	43.11 ± 24.48	<0.001	<0.001	<0.001

Notes. *P*1, pre-DM vs. controls; *P*2, T2DM vs. controls.

**Table 2:** Genotypes and allele frequencies for the *SLC2A9* A/G (rs1014290) SNP in the control, prediabetic, and type 2 diabetic groups.

Group	AA *n* (%)	AG *n* (%)	GG *n* (%)	MAF (G allete) (%)	GG vs. (AG + AA)		GG vs. AA		(GG + AG) vs. AA		(AA + GG) vs. AG	
					*χ* ^2^	*P*	*χ* ^2^	*P*	*χ* ^2^	*P*	*χ* ^2^	*P*
Controls (*n* = 348)	104 (29.9)	173 (49.7)	71 (20.4)	45.2	—	—	—	—	—	—	—	—
Pre-DM (*n* = 358)	115 (32.1)	180 (50.3)	63 (17.6)	42.7	0.903	0.342	1.005	0.316	0.413	0.520	0.023	0.880
T2DM (*n* = 352)	128 (36.4)	172 (48.9)	52 (14.8)	39.2	3.829	0.050^*∗*^	5.348	0.021^*∗*^	1.290	0.256	0.050	0.822

Notes. Values are shown as *n* (%). All three groups were in Hardy–Weinberg equilibrium.

**Table 3:** Binary logistic regression analysis for T2DM risk.

rs1014290 Parameters	Unadjusted		Adjusted model 1		Adjusted model 2		Adjusted model 3	
	OR (95%CI)	*P*	OR (95%CI)	*P*	OR (95%CI)	*P*	OR (95%CI)	*P*
AA	1 (Reference)	—	1 (Reference)	—	1 (Reference)	—	1 (Reference)	—
GG	0.595 (0.383–0.925)	0.021^*∗*^	0.357 (0.197–0.646)	0.001^*∗*^	0.278 (0.078–0.990)	0.048^*∗*^	0.248 (0.063–0.966)	0.045^*∗*^
AG	0.808 (0.578–1.128)	0.210	0.484 (0.265–0.884)	0.018^*∗*^	0.437 (0.121–1.569)	0.204	0.465 (0.115–1.888)	0.284

Notes. Model 1, adjusted for age, BMI, and sex; model 2, adjusted for age, BMI, sex, and uric acid; model 3, adjusted for age, BMI, sex, uric acid, cholesterol, HDL, LDL, and triglyceride.

**Table 4:** Binary logistic regression analysis for pre-DM risk.

rs1014290 Parameters	Unadjusted		Adjusted model 1		Adjusted model 2		Adjusted model 3	
	OR [95%CI]	*P*	OR [95%CI]	*P*	OR [95%CI]	*P*	OR [95%CI]	*P*
AA	1 (Reference)	—	1 (Reference)	—	1 (Reference)	—	1 (Reference)	—
GG	0.802 (0.522–1.234)	0.316	0.794 (0.473–1.332)	0.382	0.593 (0.289–1.217)	0.154	0.683 (0.382–1.423)	0.309
AG	0.941 (0.671–1.319)	0.724	1.131 (0.660–1.938)	0.655	0.926 (0.442–1.941)	0.838	1.025 (0.476–2.211)	0.949

Notes. Model 1, adjusted for age, BMI, and sex; model 2, adjusted for age, BMI, sex, and uric acid; model 3, adjusted for age, BMI, sex, uric acid, cholesterol, HDL, LDL, and triglycerides.

**Table 5:** Comparisons of biochemical indices of the *SLC2A9* A/G (rs1014290) SNP among the different genotypes in the pre-DM group.

Variables	Genotype groups			Pairwise comparisons			
	AA (*n* = 115)	AG (*n* = 180)	GG (*n* = 63)	All *P*	*P*1	*P*2	*P*3
Age (years)	52.88 ± 11.77	54.26 ± 11.27	51.08 ± 11.51	0.153	0.313	0.318	0.059
Male/female (*n*)	53/62	84/96	28/35	0.955	0.922	0.833	0.761
BMI (kg/m^2^)	27.06 ± 3.70	27.09 ± 3.89	26.57 ± 3.98	0.636	0.958	0.414	0.359
SBP (mmHg)	127.5 ± 14.02	128.15 ± 12.90	123.52 ± 13.16	0.057	0.685	0.057	0.018
DBP (mmHg)	77.18 ± 9.23	76.96 ± 9.27	77.59 ± 9.54	0.898	0.842	0.782	0.646
UA (umol/L)	316.78 ± 26.76	320.08 ± 45.59	321.41 ± 23.79	0.667	0.457	0.426	0.806
TG (mmol/L)	1.63 ± 1.08	1.89 ± 1.74	1.65 ± 1.06	0.260	0.140	0.956	0.252
TC (mmol/L)	5.02 ± 0.94	5.02 ± 0.97	4.78 ± 0.98	0.202	0.972	0.110	0.092
HDL (mmol/L)	1.25 ± 0.23	1.30 ± 0.26	1.26 ± 0.23	0.223	0.094	0.698	0.341
LDL (mmol/L)	3.16 ± 0.81	3.05 ± 0.78	3.01 ± 0.73	0.415	0.275	0.241	0.714
FPG (mmol/L)	6.45 ± 0.30	5.61 ± 0.21	4.91 ± 0.22	<0.001	<0.001	<0.001	<0.001
PBG (mmol/L)	7.49 ± 1.73	8.47 ± 0.91	8.69 ± 0.95	<0.001	<0.001	<0.001	0.222
FINS (*μ*U/mL)	9.50 ± 3.29	8.63 ± 3.07	9.16 ± 3.44	0.071	0.024	0.492	0.265
Postprandial FINS (*μ*U/mL)	60.64 ± 21.67	67.29 ± 20.85	67.91 ± 25.35	0.024	0.012	0.036	0.848
HbA1c (%)	5.79 ± 0.43	5.78 ± 0.36	5.71 ± 0.37	0.350	0.763	0.163	0.212
HOMA-IR	2.73 ± 0.96	2.15 ± 0.78	2.00 ± 0.77	<0.001	<0.001	<0.001	0.521
HOMA-*β*	64.93 ± 23.00	82.50 ± 30.22	131.94 ± 51.47	<0.001	<0.001	<0.001	<0.001

Notes. *P*1, GA vs. AA; *P*2, GG vs. AA; *P*3, GG vs. GA.

**Table 6:** Comparison of biochemical indices of the *SLC2A9* A/G (rs1014290) SNP among the different genotypes in the T2DM group.

Variables	Genotype groups			Pairwise comparisons			
	AA (*n* = 128)	AG (*n* = 172)	GG (*n* = 52)	All *P*	*P*1	*P*2	*P*3
Age (years)	58.84 ± 10.48	59.41 ± 10.38	58.71 ± 9.20	0.857	0.638	0.938	0.668
Male/female (*n*)	66/62	71/101	22/30	0.187	0.095	0.174	0.850
BMI (kg/m^2^)	27.09 ± 3.47	26.88 ± 3.92	26.44 ± 3.60	0.575	0.637	0.294	0.457
SBP (mmHg)	132.79 ± 12.91	130.95 ± 12.96	129.62 ± 14.56	0.277	0.232	0.144	0.524
DBP (mmHg)	79.45 ± 8.62	77.47 ± 8.28	77.42 ± 10.91	0.127	0.055	0.163	0.973
UA (umol/L)	430.52 ± 45.67	441.06 ± 64.30	428.56 ± 58.97	<0.001	0.266	<0.001	<0.001
TG (mmol/L)	2.41 ± 2.98	1.74 ± 1.03	1.41 ± 0.69	0.002	0.004	0.002	0.275
TC (mmol/L)	5.22 ± 1.07	5.03 ± 1.02	4.81 ± 0.94	0.045	0.127	0.016	0.164
HDL (mmol/L)	1.30 ± 0.32	1.30 ± 0.30	1.30 ± 0.34	0.979	0.874	0.854	0.940
LDL (mmol/L)	3.06 ± 0.80	2.94 ± 0.87	2.84 ± 0.87	0.229	0.217	0.111	0.455
FPG (mmol/L)	9.85 ± 1.30	7.02 ± 0.57	5.53 ± 0.30	<0.001	<0.001	<0.001	<0.001
PBG (mmol/L)	14.42 ± 2.09	12.24 ± 2.42	10.03 ± 2.28	<0.001	<0.001	<0.001	<0.001
FINS (*μ*U/mL)	7.85 ± 2.89	7.88 ± 2.82	7.41 ± 2.34	0.558	0.934	0.342	0.295
Postprandial FINS (*μ*U/mL)	43.51 ± 16.41	49.40 ± 15.70	52.00 ± 14.92	0.001	0.002	0.001	0.307
HbA1c (%)	8.05 ± 1.67	7.12 ± 1.25	6.22 ± 0.92	<0.001	<0.001	<0.001	<0.001
HOMA-IR	3.40 ± 1.22	2.45 ± 0.88	1.82 ± 0.58	<0.001	<0.001	<0.001	<0.001
HOMA-*β*	26.14 ± 12.03	46.24 ± 19.40	74.53 ± 27.21	<0.001	<0.001	<0.001	<0.001

Notes. *P*1, GA vs. AA; *P* 2, GG vs. AA; *P* 3, GG vs. GA.

## Data Availability

The data can be obtained by e-mail request to the corresponding author.

## Abbreviations

UA: Uric acid
BMI: Body mass index
DBP: Diastolic blood pressure
FINS: Fasting insulin
FPG: Fasting plasma glucose
HbA1c: Glycosylated hemoglobin
HDL: High-density lipoprotein
HOMA-IR/*β*: Homeostasis model assessment-insulin resistance/*β*-cell function
LDL: Low-density lipoprotein
LPL: Lipoprotein lipase
PBG: Postprandial blood glucose
Pre-DM: Prediabetes
SBP: Systolic blood pressure
T2DM: Type 2 diabetes mellitus
TC: Total cholesterol
TG: Triglycerides
MAF: Minor allele frequency
OR: Odds ratio
CI: Confidence interval.

## Supplementary Materials

Supplementary Materials Original data-1. Collect general information such as age, gender, body mass index, and blood pressure. We use an automatic biochemical analyzer to detect blood glucose at each time of the glucose tolerance test, uric acid (UA), and blood lipids (including TC, TG, LDL, and HDL) (Hitachi 7600-110, Japan). Detection of glycated hemoglobin (HbA1c) by chromatographic analysis (ADAMS A1c HA-8180 analyzer, Japan). Determination of serum insulin levels by electrochemiluminescence (Roche Cobas e601, Germany). The degree of insulin resistance was assessed by calculating beta cell function (HOMA-*β*) and the insulin resistance index (HOMA-IR), using the HOMA2 calculator version 2.2.3 (Oxford University Diabetes Trial Unit). Original data-2. Genotyping and determination of gene polymorphisms were carried out with reference to the literature [20]. Genomic DNA extraction kit (Generay Biotechnology, Shanghai, China). Genotyping of patient SLC2A9 by DNA sequencing (ABI3730 genetic analyzer, Applied Biosystems, USA). The primers for gene amplification were as follows: 5'-GGATTCACAACTATCTTACTCAT-3' (forward); 5'-CAGGGTTATGTTCCATTTATCT-3' (reverse); designed using Primer Premier 5.0 (Premier Biosoft International, CA, USA). The samples were sequenced using GeneMarker v2.2.0 (PA, USA).
