# Supplementary material for: SLC2A9 rs1014290 Polymorphism is Associated with Prediabetes and Type 2 Diabetes
Source: Int J Endocrinol. 2022 Dec 12;2022:4947684. doi: 10.1155/2022/4947684 (PMC9763018; doi:10.1155/2022/4947684)
Supplement: Supplementary Materials — Original data-1. Collect general information such as age, gender, body mass index, and blood pressure. We use an automatic biochemical analyzer to detect blood glucose at each time of the glucose tolerance test, uric acid (UA), and blood lipids (including TC, TG, LDL, and HDL) (Hitachi 7600-110, Japan). Detection of glycated hemoglobin (HbA1c) by chromatographic analysis (ADAMS A1c HA-8180 analyzer, Japan). Determination of serum insulin levels by electrochemiluminescence (Roche Cobas e601, Germany). The degree of insulin resistance was assessed by calculating beta cell function (HOMA-β) and the insulin resistance index (HOMA-IR), using the HOMA2 calculator version 2.2.3 (Oxford University Diabetes Trial Unit). Original data-2. Genotyping and determination of gene polymorphisms were carried out with reference to the literature [20]. Genomic DNA extraction kit (Generay Biotechnology, Shanghai, China). Genotyping of patient SLC2A9 by DNA sequencing (ABI3730 genetic analyzer, Applied Biosystems, USA). The primers for gene amplification were as follows: 5'-GGATTCACAACTATCTTACTCAT-3' (forward); 5'-CAGGGTTATGTTCCATTTATCT-3' (reverse); designed using Primer Premier 5.0 (Premier Biosoft International, CA, USA). The samples were sequenced using GeneMarker v2.2.0 (PA, USA). [file 4947684.f1.zip › original data-1.docx]

Concise supplementary material description.

Collect general information such as age, gender, body mass index, blood pressure. We use an automatic biochemical analyzer to detect blood glucose at each time of the glucose tolerance test, uric acid (UA), and blood lipids (including TC, TG, LDL, HDL) (Hitachi 7600-110, Japan). Detection of Glycated Hemoglobin (HbA1c) by Chromatographic Analysis (ADAMS A1c HA-8180 analyzer, Japan). Determination of serum insulin levels by electrochemiluminescence (Roche Cobas e601, Germany). The degree of insulin resistance was assessed by calculating beta cell function (HOMA-β) and the insulin resistance index (HOMA-IR), using the HOMA2 calculator version 2.2.3 (Oxford University Diabetes Trial Unit).

| group | gender | age | SBP | DBP | BMI | TC | TG | HDL | LDL | FBG | PBG | FINS | PINS | HbA1C | HOME-IR | HOME-B | UA |
| --- | --- | --- | --- | --- | --- | --- | --- | --- | --- | --- | --- | --- | --- | --- | --- | --- | --- |
| 1 | 1 | 27 | 135 | 90 | 19.80 | 2.83 | 0.79 | 0.97 | 1.66 | 4.82 | 4.77 | 7.95 | 21.57 | 6.1 | 1.70 | 120.45 | 134 |
| 1 | 1 | 26 | 126 | 73 | 21.90 | 4.07 | 0.72 | 1.31 | 2.38 | 4.71 | 4.66 | 17.59 | 29.25 | 5.1 | 3.68 | 290.74 | 159 |
| 1 | 1 | 34 | 105 | 70 | 24.10 | 4.90 | 1.58 | 1.45 | 2.92 | 5.31 | 4.87 | 6.44 | 21.99 | 5.4 | 1.52 | 71.16 | 164 |
| 1 | 1 | 26 | 115 | 73 | 18.10 | 3.61 | 0.53 | 1.18 | 2.08 | 4.8 | 4.51 | 7.71 | 28.35 | 5.5 | 1.73 | 118.62 | 169 |
| 1 | 1 | 27 | 121 | 72 | 19.10 | 4.32 | 0.94 | 1.24 | 2.61 | 5 | 5.46 | 11.03 | 27.79 | 5.3 | 2.45 | 147.07 | 178 |
| 1 | 1 | 26 | 130 | 80 | 26.70 | 3.85 | 1.52 | 0.98 | 2.58 | 5.24 | 6.72 | 17.43 | 86 | 6.3 | 4.06 | 200.34 | 180 |
| 1 | 1 | 27 | 126 | 84 | 24.50 | 5.11 | 0.80 | 1.24 | 3.43 | 5.42 | 5.07 | 9.71 | 53.31 | 5.9 | 2.34 | 101.15 | 180 |
| 1 | 1 | 44 | 129 | 80 | 24.80 | 4.75 | 0.81 | 1.20 | 3.13 | 4.8 | 4.21 | 5.67 | 30.38 | 6.2 | 1.21 | 87.23 | 181 |
| 1 | 1 | 65 | 153 | 85 | 21.20 | 6.24 | 1.33 | 1.57 | 3.99 | 4.96 | 5.44 | 6.36 | 11.3 | 5.3 | 1.40 | 87.12 | 181 |
| 1 | 1 | 28 | 125 | 70 | 26.90 | 4.49 | 1.56 | 0.97 | 2.97 | 4.66 | 4.71 | 19.32 | 23.92 | 5.8 | 4.00 | 333.10 | 182 |
| 1 | 1 | 26 | 136 | 88 | 29.70 | 6.07 | 5.72 | 1.29 | 3.80 | 5.35 | 6.54 | 18.2 | 82.6 | 5.4 | 4.33 | 196.76 | 183 |
| 1 | 1 | 58 | 117 | 75 | 26.10 | 4.35 | 2.63 | 1.28 | 2.67 | 5.29 | 3.77 | 10.25 | 20.65 | 6.3 | 2.41 | 114.53 | 185 |
| 1 | 1 | 32 | 141 | 96 | 28.60 | 3.29 | 0.93 | 1.91 | 2.12 | 4.94 | 4.32 | 9.04 | 67.17 | 5.8 | 1.98 | 125.56 | 185 |
| 1 | 1 | 25 | 134 | 88 | 34.00 | 6.45 | 4.65 | 1.07 | 4.32 | 4.66 | 5.92 | 13.04 | 53.57 | 5.9 | 2.79 | 224.83 | 187 |
| 1 | 1 | 49 | 124 | 77 | 24.40 | 5.03 | 0.80 | 1.36 | 3.29 | 4.93 | 4.93 | 6.89 | 19.76 | 5.5 | 1.51 | 96.36 | 190 |
| 1 | 1 | 53 | 116 | 72 | 29.70 | 4.66 | 1.40 | 1.16 | 3.09 | 5.12 | 4.01 | 9.72 | 25.06 | 6.3 | 2.21 | 120.00 | 196 |
| 1 | 1 | 36 | 125 | 75 | 27.10 | 5.08 | 1.51 | 1.26 | 3.47 | 5.09 | 6.12 | 12.98 | 88.17 | 5.9 | 2.94 | 163.27 | 201 |
| 1 | 1 | 28 | 127 | 73 | 27.50 | 4.04 | 1.33 | 1.07 | 2.52 | 5.16 | 7.18 | 10.57 | 54.11 | 5.4 | 2.42 | 127.35 | 203 |
| 1 | 1 | 28 | 136 | 75 | 21.20 | 3.40 | 0.80 | 1.07 | 2.20 | 4.39 | 5.27 | 5.28 | 26.64 | 6.0 | 1.03 | 118.65 | 203 |
| 1 | 1 | 51 | 131 | 94 | 23.70 | 6.09 | 3.57 | 1.53 | 3.70 | 4.52 | 6.19 | 6.73 | 52.37 | 5.7 | 1.47 | 131.96 | 209 |
| 1 | 1 | 28 | 127 | 81 | 20.60 | 3.69 | 1.30 | 1.07 | 2.21 | 4.96 | 3.99 | 8.41 | 22.92 | 5.3 | 1.85 | 115.21 | 210 |
| 1 | 1 | 27 | 129 | 78 | 27.10 | 4.96 | 1.65 | 1.14 | 3.15 | 4.64 | 3.39 | 13.95 | 11.32 | 5.7 | 2.88 | 244.74 | 211 |
| 1 | 1 | 25 | 125 | 79 | 22.80 | 3.32 | 1.24 | 0.93 | 2.20 | 4.43 | 5.08 | 6.46 | 26.19 | 5.6 | 1.27 | 138.92 | 212 |
| 1 | 1 | 30 | 138 | 90 | 30.50 | 4.34 | 2.94 | 1.50 | 2.37 | 4.67 | 6.02 | 10.36 | 33.73 | 5.8 | 2.15 | 177.09 | 212 |
| 1 | 1 | 26 | 101 | 58 | 19.20 | 4.23 | 0.82 | 1.27 | 2.61 | 4.58 | 5.03 | 8.47 | 31.97 | 6.6 | 1.72 | 156.85 | 212 |
| 1 | 1 | 47 | 112 | 74 | 26.70 | 5.19 | 1.41 | 1.33 | 3.23 | 5.12 | 4.95 | 7.46 | 42.48 | 5.4 | 1.40 | 92.10 | 212 |
| 1 | 1 | 30 | 109 | 66 | 26.30 | 4.77 | 2.02 | 1.10 | 2.88 | 4.57 | 6.24 | 8.61 | 45.82 | 6.1 | 1.75 | 160.93 | 213 |
| 1 | 1 | 26 | 128 | 78 | 20.80 | 3.61 | 0.66 | 1.10 | 2.28 | 4.98 | 6.1 | 11.13 | 27.51 | 5.7 | 2.46 | 150.41 | 218 |
| 1 | 1 | 27 | 115 | 80 | 22.90 | 4.00 | 0.91 | 1.29 | 2.44 | 5.38 | 6.18 | 11.06 | 55.54 | 5.9 | 2.35 | 117.66 | 218 |
| 1 | 1 | 22 | 109 | 73 | 19.30 | 4.12 | 1.38 | 1.37 | 2.34 | 5.52 | 5.65 | 7.48 | 33.42 | 5.6 | 1.84 | 74.06 | 221 |
| 1 | 1 | 27 | 130 | 80 | 29.20 | 4.00 | 1.77 | 0.98 | 2.38 | 4.81 | 5.63 | 16.42 | 67.6 | 5.7 | 3.51 | 250.69 | 222 |
| 1 | 1 | 27 | 130 | 78 | 23.10 | 4.18 | 1.00 | 1.11 | 2.77 | 4.93 | 3.71 | 17.88 | 61.13 | 5.8 | 3.92 | 250.07 | 223 |
| 1 | 1 | 26 | 142 | 81 | 25.30 | 3.86 | 0.70 | 1.12 | 2.34 | 4.18 | 5.27 | 13.06 | 74.74 | 6.1 | 2.43 | 384.12 | 226 |
| 1 | 1 | 37 | 130 | 87 | 25.04 | 4.59 | 1.25 | 0.99 | 2.86 | 5.22 | 6.91 | 13.25 | 94.9 | 6.1 | 3.07 | 154.07 | 227 |
| 1 | 1 | 27 | 137 | 86 | 33.00 | 5.45 | 2.91 | 1.11 | 3.90 | 4.8 | 5.9 | 14.61 | 85.32 | 5.5 | 3.12 | 224.77 | 227 |
| 1 | 1 | 43 | 138 | 90 | 25.30 | 4.85 | 0.94 | 1.28 | 2.96 | 5.45 | 7 | 7.24 | 23.13 | 5.7 | 1.41 | 74.26 | 227 |
| 1 | 1 | 42 | 130 | 80 | 25.40 | 3.60 | 2.68 | 0.74 | 2.35 | 4.85 | 5.89 | 11.91 | 103.5 | 5.4 | 2.57 | 176.44 | 228 |
| 1 | 1 | 26 | 120 | 80 | 22.80 | 4.07 | 1.43 | 0.94 | 2.54 | 4.4 | 4.69 | 8.39 | 27.8 | 5.9 | 1.64 | 186.44 | 228 |
| 1 | 1 | 49 | 139 | 95 | 28.90 | 5.49 | 1.22 | 1.82 | 3.67 | 5.02 | 2.8 | 9.04 | 12.48 | 4.9 | 2.02 | 118.95 | 229 |
| 1 | 1 | 37 | 130 | 80 | 27.00 | 5.53 | 2.12 | 1.24 | 3.71 | 5.47 | 5.69 | 9.9 | 42.93 | 5.6 | 2.41 | 100.51 | 230 |
| 1 | 1 | 26 | 134 | 86 | 26.60 | 3.58 | 1.04 | 1.02 | 2.28 | 4.92 | 5.3 | 8.45 | 33.67 | 5.3 | 1.69 | 119.01 | 230 |
| 1 | 1 | 49 | 156 | 100 | 32.70 | 4.72 | 2.24 | 0.91 | 3.05 | 4.81 | 6.9 | 13.04 | 82.6 | 5.2 | 2.70 | 199.08 | 231 |
| 1 | 1 | 28 | 125 | 84 | 25.40 | 3.40 | 0.86 | 0.92 | 2.09 | 4.61 | 5.24 | 8.41 | 21.89 | 4.9 | 1.72 | 151.53 | 231 |
| 1 | 1 | 56 | 100 | 70 | 21.70 | 3.35 | 0.38 | 1.31 | 1.97 | 5.54 | 4.59 | 8.32 | 18.34 | 5.4 | 2.05 | 81.57 | 233 |
| 1 | 1 | 49 | 133 | 81 | 18.50 | 4.96 | 1.63 | 1.06 | 3.11 | 5.26 | 4.75 | 6.31 | 29.74 | 5.2 | 1.48 | 71.70 | 234 |
| 1 | 1 | 40 | 124 | 73 | 25.20 | 5.19 | 1.06 | 1.07 | 3.55 | 4.88 | 6.34 | 18.69 | 20 | 5.3 | 4.11 | 270.87 | 234 |
| 1 | 1 | 47 | 123 | 70 | 34.40 | 4.06 | 1.21 | 1.04 | 2.59 | 4.9 | 6.13 | 7.35 | 58.98 | 5.1 | 1.60 | 105.00 | 234 |
| 1 | 1 | 28 | 115 | 66 | 23.60 | 2.91 | 0.95 | 0.89 | 1.78 | 4.92 | 5.89 | 8.19 | 49 | 5.2 | 1.79 | 115.35 | 234 |
| 1 | 1 | 30 | 149 | 87 | 32.90 | 4.19 | 3.93 | 0.92 | 2.65 | 5.01 | 6.98 | 18.5 | 83.49 | 5.6 | 4.12 | 245.03 | 235 |
| 1 | 1 | 52 | 129 | 89 | 30.20 | 4.12 | 3.07 | 0.97 | 2.58 | 5.31 | 5.42 | 12.7 | 50.76 | 5.2 | 3.00 | 140.33 | 235 |
| 1 | 1 | 59 | 123 | 62 | 24.40 | 4.05 | 0.94 | 1.76 | 2.18 | 5.13 | 6.33 | 8.31 | 59.68 | 5.2 | 1.92 | 101.96 | 235 |
| 1 | 1 | 28 | 115 | 80 | 29.40 | 4.99 | 0.95 | 1.40 | 3.07 | 5.02 | 5.06 | 15.91 | 73.45 | 5.1 | 3.55 | 209.34 | 235 |
| 1 | 1 | 50 | 134 | 95 | 29.30 | 4.10 | 1.23 | 1.11 | 2.45 | 4.72 | 5.07 | 6.89 | 17.54 | 5.5 | 1.45 | 112.95 | 238 |
| 1 | 1 | 44 | 119 | 69 | 30.10 | 5.07 | 2.07 | 0.92 | 3.59 | 4.99 | 4.24 | 13.85 | 47.07 | 5.2 | 3.07 | 185.91 | 242 |
| 1 | 1 | 51 | 129 | 80 | 25.50 | 4.55 | 1.45 | 1.87 | 3.17 | 5.19 | 7.23 | 6.95 | 34.75 | 4.9 | 1.54 | 82.25 | 242 |
| 1 | 1 | 37 | 131 | 80 | 30.00 | 5.60 | 4.21 | 1.12 | 3.80 | 4.52 | 6.25 | 17.47 | 71.88 | 5.1 | 3.51 | 342.55 | 242 |
| 1 | 1 | 63 | 145 | 77 | 27.70 | 5.02 | 0.65 | 1.42 | 3.32 | 5.03 | 6.69 | 5.09 | 68.69 | 5.3 | 1.14 | 66.54 | 242 |
| 1 | 1 | 26 | 137 | 91 | 32.80 | 5.94 | 2.07 | 1.25 | 4.12 | 5.44 | 6.85 | 17.83 | 84.25 | 5.5 | 4.31 | 183.81 | 242 |
| 1 | 1 | 25 | 134 | 75 | 28.60 | 3.62 | 2.07 | 0.92 | 2.33 | 5.19 | 6.4 | 9.87 | 61.62 | 5.8 | 2.28 | 116.80 | 342 |
| 1 | 1 | 26 | 162 | 86 | 32.00 | 3.20 | 1.06 | 1.15 | 1.99 | 4.51 | 4.64 | 10.46 | 26.72 | 5.9 | 2.38 | 207.13 | 243 |
| 1 | 1 | 30 | 133 | 80 | 25.10 | 3.32 | 0.63 | 1.16 | 2.07 | 4.85 | 5.98 | 8.8 | 45.38 | 5.3 | 1.90 | 130.37 | 244 |
| 1 | 1 | 34 | 128 | 78 | 24.10 | 3.66 | 0.74 | 1.24 | 2.24 | 5.14 | 6.62 | 16.03 | 54.36 | 5.2 | 3.66 | 195.49 | 244 |
| 1 | 1 | 25 | 132 | 78 | 23.80 | 4.53 | 1.29 | 1.23 | 2.95 | 5.13 | 5.83 | 10.46 | 43.08 | 5.9 | 2.10 | 128.34 | 245 |
| 1 | 1 | 66 | 140 | 88 | 24.60 | 6.12 | 1.69 | 1.45 | 4.05 | 5.02 | 5.47 | 6.4 | 24.62 | 5.5 | 1.35 | 84.21 | 245 |
| 1 | 1 | 27 | 132 | 75 | 23.50 | 4.62 | 2.59 | 1.07 | 2.92 | 4.46 | 6.08 | 13.31 | 59.6 | 5.7 | 2.64 | 277.29 | 245 |
| 1 | 1 | 54 | 104 | 62 | 28.20 | 5.52 | 1.42 | 1.06 | 3.87 | 5.42 | 5.5 | 10.94 | 55.64 | 6.1 | 2.64 | 113.96 | 245 |
| 1 | 1 | 45 | 150 | 100 | 29.60 | 4.41 | 3.21 | 0.93 | 2.91 | 5.16 | 5.8 | 13.74 | 61.6 | 6.0 | 3.15 | 165.54 | 245 |
| 1 | 1 | 41 | 130 | 97 | 28.30 | 5.48 | 1.94 | 1.29 | 3.65 | 4.95 | 5.76 | 8.14 | 94.3 | 5.8 | 1.79 | 112.28 | 245 |
| 1 | 1 | 29 | 148 | 84 | 34.00 | 4.29 | 4.88 | 0.82 | 2.73 | 5.55 | 5.72 | 17.46 | 45.7 | 5.9 | 4.31 | 170.34 | 246 |
| 1 | 1 | 39 | 110 | 70 | 24.80 | 4.06 | 1.77 | 0.94 | 2.67 | 5.14 | 5.85 | 9.04 | 44 | 5.5 | 2.07 | 110.24 | 346 |
| 1 | 1 | 49 | 150 | 87 | 25.70 | 3.60 | 1.00 | 0.69 | 2.67 | 4.99 | 6.64 | 6.75 | 81.77 | 5.5 | 1.50 | 90.60 | 246 |
| 1 | 1 | 52 | 125 | 77 | 30.00 | 3.32 | 1.03 | 1.12 | 2.03 | 5.18 | 5.3 | 11.46 | 54.52 | 6.0 | 2.64 | 136.43 | 246 |
| 1 | 1 | 68 | 137 | 91 | 29.00 | 4.35 | 0.73 | 1.18 | 2.76 | 5.48 | 5.1 | 10.24 | 31.44 | 5.3 | 2.49 | 103.43 | 247 |
| 1 | 1 | 37 | 139 | 97 | 23.10 | 4.11 | 0.98 | 1.27 | 2.65 | 5.33 | 6.05 | 7.92 | 94.35 | 5.5 | 1.88 | 86.56 | 248 |
| 1 | 1 | 25 | 120 | 80 | 22.10 | 4.05 | 0.94 | 1.66 | 2.19 | 4.56 | 3.01 | 14.93 | 50.69 | 5.5 | 3.03 | 281.70 | 248 |
| 1 | 1 | 36 | 109 | 61 | 25.10 | 4.21 | 1.48 | 0.84 | 3.01 | 5.29 | 6.74 | 12.66 | 87.33 | 6.4 | 2.98 | 141.45 | 248 |
| 1 | 1 | 31 | 128 | 69 | 20.70 | 4.54 | 0.54 | 1.51 | 2.89 | 4.11 | 5.92 | 12.55 | 44.61 | 5.4 | 2.22 | 411.48 | 249 |
| 1 | 1 | 34 | 123 | 76 | 27.90 | 4.54 | 2.03 | 1.07 | 3.02 | 5.29 | 6.64 | 17.04 | 63.5 | 5.8 | 4.01 | 190.39 | 249 |
| 1 | 1 | 31 | 136 | 88 | 29.70 | 4.33 | 1.18 | 1.07 | 2.85 | 5.3 | 7.28 | 13.88 | 61.36 | 5.8 | 3.27 | 154.22 | 249 |
| 1 | 1 | 52 | 158 | 95 | 26.20 | 2.62 | 2.31 | 0.81 | 1.50 | 5.39 | 6.19 | 9.55 | 45.95 | 6.0 | 2.29 | 101.06 | 249 |
| 1 | 1 | 31 | 131 | 68 | 23.50 | 4.52 | 1.34 | 1.24 | 2.87 | 5.15 | 5.35 | 12.56 | 62.18 | 5.5 | 2.87 | 152.24 | 249 |
| 1 | 1 | 39 | 141 | 90 | 26.80 | 5.05 | 2.09 | 1.10 | 3.42 | 5.55 | 3.42 | 11.43 | 15.87 | 5.5 | 2.82 | 111.51 | 249 |
| 1 | 1 | 55 | 119 | 84 | 25.70 | 5.40 | 1.88 | 1.26 | 3.52 | 5.58 | 5.02 | 10.81 | 13.14 | 5.6 | 2.68 | 103.94 | 250 |
| 1 | 1 | 62 | 137 | 88 | 27.00 | 6.18 | 6.54 | 1.34 | 3.56 | 5.25 | 5.47 | 7.44 | 40.37 | 5.4 | 1.79 | 85.03 | 250 |
| 1 | 1 | 40 | 122 | 74 | 27.00 | 4.48 | 1.96 | 0.97 | 2.97 | 5.04 | 5.96 | 7.71 | 52.06 | 5.5 | 1.64 | 100.13 | 250 |
| 1 | 1 | 48 | 129 | 82 | 25.70 | 4.92 | 0.90 | 1.49 | 3.05 | 5.09 | 6.51 | 6.75 | 33.19 | 5.4 | 1.53 | 84.91 | 250 |
| 1 | 1 | 50 | 116 | 68 | 23.60 | 3.86 | 1.32 | 1.05 | 2.51 | 4.44 | 4.69 | 12.78 | 18.23 | 5.5 | 2.52 | 271.91 | 251 |
| 1 | 1 | 38 | 147 | 82 | 25.20 | 3.39 | 0.84 | 1.17 | 2.07 | 5.07 | 4.92 | 6.8 | 13.1 | 5.6 | 1.53 | 86.62 | 251 |
| 1 | 1 | 24 | 122 | 68 | 34.00 | 2.79 | 0.97 | 1.23 | 1.59 | 4.55 | 4.99 | 18.57 | 76.97 | 5.1 | 3.76 | 353.71 | 251 |
| 1 | 1 | 55 | 176 | 98 | 35.60 | 4.83 | 1.82 | 0.88 | 3.25 | 5.28 | 3.9 | 17.51 | 31.9 | 6.3 | 4.11 | 196.74 | 252 |
| 1 | 1 | 42 | 158 | 92 | 30.10 | 3.45 | 1.80 | 1.84 | 2.26 | 5.32 | 3.86 | 10.48 | 23.57 | 5.4 | 2.48 | 115.16 | 253 |
| 1 | 1 | 38 | 125 | 78 | 21.20 | 5.41 | 1.53 | 1.57 | 3.34 | 4.74 | 5.78 | 12.55 | 11.85 | 5.0 | 2.79 | 202.42 | 253 |
| 1 | 1 | 42 | 136 | 86 | 22.50 | 3.27 | 1.11 | 0.87 | 2.16 | 5.19 | 5.54 | 9.77 | 36.78 | 5.6 | 2.25 | 115.62 | 253 |
| 1 | 1 | 56 | 143 | 89 | 24.60 | 4.77 | 2.80 | 1.85 | 3.39 | 5.03 | 4.55 | 7.33 | 15.13 | 5.4 | 1.64 | 95.82 | 253 |
| 1 | 1 | 48 | 125 | 81 | 28.30 | 4.27 | 2.80 | 0.90 | 2.79 | 5.08 | 5.18 | 11.37 | 54.49 | 5.2 | 2.57 | 143.92 | 254 |
| 1 | 1 | 42 | 162 | 100 | 29.60 | 4.09 | 1.85 | 1.36 | 2.36 | 5.24 | 3.94 | 13.66 | 24.67 | 5.4 | 3.18 | 157.01 | 254 |
| 1 | 1 | 37 | 128 | 77 | 23.40 | 5.95 | 7.26 | 1.11 | 3.52 | 4.63 | 4.3 | 8.59 | 58.5 | 5.4 | 1.77 | 152.04 | 255 |
| 1 | 1 | 49 | 152 | 76 | 28.80 | 4.96 | 2.42 | 1.63 | 2.98 | 5.19 | 5.17 | 6.39 | 20.12 | 5.5 | 1.47 | 75.62 | 255 |
| 1 | 1 | 35 | 108 | 64 | 21.21 | 5.01 | 1.13 | 1.40 | 3.22 | 5.03 | 4.73 | 17.58 | 20.21 | 5.9 | 3.93 | 229.80 | 255 |
| 1 | 1 | 25 | 110 | 63 | 18.40 | 2.95 | 0.60 | 1.29 | 1.71 | 4.5 | 4.34 | 6.17 | 17.94 | 5.9 | 1.23 | 123.40 | 255 |
| 1 | 1 | 34 | 147 | 85 | 24.60 | 5.36 | 1.69 | 1.15 | 3.58 | 5.55 | 5.72 | 12.82 | 77.61 | 5.3 | 2.56 | 125.07 | 255 |
| 1 | 1 | 48 | 131 | 77 | 24.60 | 3.94 | 1.95 | 1.54 | 2.54 | 4.56 | 3.52 | 6.9 | 26.46 | 5.1 | 1.40 | 130.19 | 255 |
| 1 | 1 | 41 | 135 | 80 | 26.60 | 5.03 | 1.07 | 1.02 | 3.37 | 4.89 | 3.38 | 9.54 | 56.7 | 5.8 | 2.07 | 137.27 | 256 |
| 1 | 1 | 41 | 146 | 95 | 29.40 | 5.68 | 3.81 | 1.19 | 3.75 | 5.21 | 4.02 | 9.11 | 68.96 | 5.6 | 2.11 | 106.55 | 256 |
| 1 | 1 | 44 | 136 | 82 | 25.50 | 4.59 | 0.91 | 1.28 | 3.05 | 5.24 | 5.93 | 10.37 | 51.88 | 5.7 | 2.42 | 119.20 | 256 |
| 1 | 1 | 52 | 113 | 76 | 25.60 | 3.30 | 0.61 | 1.20 | 2.01 | 4.75 | 5.11 | 6.54 | 27.89 | 6.4 | 1.38 | 104.64 | 257 |
| 1 | 1 | 30 | 137 | 78 | 26.00 | 3.89 | 3.13 | 1.86 | 2.46 | 4.58 | 5.24 | 6.43 | 29.67 | 5.5 | 1.31 | 119.07 | 257 |
| 1 | 1 | 28 | 143 | 82 | 31.90 | 4.99 | 1.47 | 1.06 | 3.42 | 4.85 | 2.88 | 14.01 | 28.16 | 5.7 | 3.02 | 207.56 | 257 |
| 1 | 1 | 43 | 142 | 96 | 25.43 | 5.70 | 1.46 | 1.57 | 3.38 | 5.13 | 3.42 | 7.76 | 11.95 | 5.4 | 1.77 | 95.21 | 258 |
| 1 | 1 | 30 | 138 | 91 | 25.72 | 3.52 | 1.03 | 1.25 | 2.00 | 4.58 | 2.82 | 10.54 | 16.92 | 5.4 | 2.15 | 195.19 | 258 |
| 1 | 1 | 30 | 145 | 79 | 27.20 | 2.97 | 1.04 | 1.26 | 1.60 | 5.32 | 5.26 | 8.34 | 36.07 | 5.3 | 1.97 | 91.65 | 258 |
| 1 | 1 | 30 | 139 | 73 | 29.76 | 4.36 | 1.97 | 0.91 | 2.77 | 4.84 | 5.91 | 10.34 | 47.21 | 5.6 | 2.22 | 154.33 | 258 |
| 1 | 1 | 40 | 141 | 91 | 20.25 | 4.38 | 1.07 | 1.70 | 2.32 | 4.5 | 3.9 | 8.3 | 26.2 | 6.0 | 1.66 | 166.00 | 259 |
| 1 | 1 | 68 | 148 | 67 | 29.00 | 4.63 | 2.10 | 1.85 | 3.02 | 2.3 | 4.49 | 13.39 | 15.78 | 5.5 | 1.37 | (223.17) | 259 |
| 1 | 1 | 60 | 119 | 79 | 26.06 | 4.82 | 0.68 | 1.02 | 3.22 | 5 | 6.47 | 6.7 | 31.64 | 5.8 | 1.49 | 89.33 | 261 |
| 1 | 1 | 60 | 120 | 73 | 23.85 | 5.30 | 1.20 | 1.36 | 3.38 | 5.02 | 5.14 | 7.31 | 14.96 | 5.4 | 1.50 | 96.18 | 261 |
| 1 | 1 | 50 | 123 | 76 | 24.38 | 5.01 | 1.01 | 1.03 | 3.33 | 5.07 | 5.89 | 7.28 | 24.13 | 5.6 | 1.64 | 92.74 | 262 |
| 1 | 1 | 38 | 121 | 77 | 21.18 | 4.49 | 0.62 | 1.99 | 2.28 | 4.98 | 5.74 | 8.3 | 58.76 | 5.4 | 1.84 | 112.16 | 264 |
| 1 | 1 | 32 | 126 | 79 | 20.36 | 4.18 | 0.50 | 1.84 | 2.13 | 4.8 | 5.19 | 6.72 | 27.8 | 5.2 | 1.43 | 103.38 | 266 |
| 1 | 1 | 61 | 114 | 75 | 21.13 | 5.09 | 1.53 | 1.48 | 2.96 | 5.31 | 6.5 | 7.39 | 81.73 | 6.0 | 1.74 | 81.66 | 266 |
| 1 | 1 | 52 | 124 | 79 | 22.92 | 4.14 | 0.80 | 1.16 | 2.38 | 5 | 7.18 | 5.93 | 49.95 | 5.1 | 1.32 | 79.07 | 266 |
| 1 | 1 | 35 | 136 | 84 | 25.25 | 6.29 | 1.24 | 1.17 | 4.11 | 5.17 | 5.81 | 6.46 | 16.22 | 5.1 | 1.48 | 77.37 | 266 |
| 1 | 1 | 58 | 130 | 68 | 30.07 | 5.68 | 1.14 | 1.57 | 3.44 | 5.43 | 5.63 | 4.67 | 37.18 | 6.0 | 1.13 | 48.39 | 269 |
| 1 | 1 | 62 | 135 | 88 | 23.40 | 5.62 | 0.87 | 1.91 | 2.82 | 5.06 | 5.92 | 12.43 | 50.00 | 5.5 | 2.65 | 159.36 | 270 |
| 1 | 1 | 57 | 116 | 72 | 25.70 | 4.94 | 1.24 | 1.67 | 2.42 | 5.57 | 5.83 | 4.73 | 15.33 | 5.7 | 1.17 | 45.70 | 270 |
| 1 | 1 | 57 | 127 | 87 | 22.20 | 4.49 | 0.9 | 1.35 | 2.79 | 4.93 | 6.68 | 5.77 | 27.41 | 5.4 | 1.26 | 80.70 | 271 |
| 1 | 1 | 38 | 103 | 57 | 30.10 | 4.56 | 0.91 | 1.28 | 2.62 | 4.89 | 6.57 | 6.35 | 38.14 | 5.7 | 1.38 | 91.37 | 271 |
| 1 | 1 | 25 | 125 | 65 | 21.23 | 4.42 | 0.9 | 2.25 | 1.6 | 5.49 | 6.25 | 5.73 | 22.14 | 5.8 | 1.30 | 57.59 | 272 |
| 1 | 1 | 32 | 140 | 90 | 25.44 | 3.85 | 1.33 | 1.38 | 1.87 | 5.53 | 6.1 | 7.43 | 54.56 | 5.5 | 1.83 | 73.20 | 273 |
| 1 | 1 | 63 | 137 | 81 | 29.40 | 3.86 | 1.21 | 0.95 | 2.36 | 5.22 | 2.84 | 11.63 | 19.66 | 6.2 | 2.70 | 135.23 | 274 |
| 1 | 1 | 62 | 128 | 86 | 34.10 | 4.17 | 1.13 | 1.34 | 2.42 | 5.01 | 5.74 | 7.27 | 27.82 | 5.9 | 1.62 | 96.29 | 274 |
| 1 | 1 | 77 | 130 | 65 | 23.40 | 3.13 | 0.39 | 1.26 | 1.54 | 5.22 | 6.24 | 6.10 | 35.18 | 5.4 | 1.42 | 70.93 | 274 |
| 1 | 1 | 45 | 113 | 75 | 26.10 | 4.19 | 0.53 | 1.34 | 2.83 | 4.95 | 6.52 | 4.95 | 18.68 | 5.7 | 1.09 | 68.28 | 275 |
| 1 | 1 | 50 | 134 | 78 | 22.50 | 2.88 | 0.71 | 1.18 | 1.02 | 5.41 | 5.72 | 8.40 | 38.90 | 5.5 | 2.02 | 87.96 | 276 |
| 1 | 1 | 65 | 145 | 95 | 20.20 | 4.81 | 0.6 | 1.78 | 2.52 | 4.84 | 7.06 | 7.22 | 89.08 | 5.2 | 1.55 | 107.76 | 398 |
| 1 | 1 | 60 | 130 | 80 | 27.40 | 4 | 0.98 | 1.22 | 2.19 | 5.31 | 5.06 | 6.59 | 27.51 | 5.5 | 1.56 | 72.82 | 277 |
| 1 | 1 | 70 | 125 | 70 | 23.40 | 5.33 | 1.4 | 1.13 | 3.55 | 4.49 | 6.24 | 5.63 | 39.33 | 5.4 | 1.12 | 113.74 | 278 |
| 1 | 1 | 30 | 127 | 70 | 30.90 | 3.57 | 0.92 | 0.96 | 2.51 | 5.44 | 6.38 | 11.50 | 32.64 | 6.0 | 2.48 | 118.56 | 279 |
| 1 | 1 | 66 | 140 | 90 | 25.70 | 5.4 | 2.11 | 0.99 | 3.32 | 5.25 | 7.12 | 10.74 | 50.44 | 5.8 | 2.51 | 122.74 | 279 |
| 1 | 1 | 40 | 130 | 75 | 36.70 | 4.47 | 2.38 | 0.88 | 2.73 | 5.09 | 3.53 | 18.29 | 39.82 | 5.1 | 4.14 | 230.06 | 281 |
| 1 | 1 | 62 | 125 | 80 | 30.20 | 3.79 | 1.34 | 1.26 | 2.24 | 4.91 | 4.35 | 8.97 | 12.91 | 5.6 | 1.96 | 127.23 | 282 |
| 1 | 1 | 26 | 135 | 80 | 27.80 | 4.27 | 2.18 | 1.18 | 2.87 | 5.24 | 5.92 | 16.38 | 55.75 | 5.6 | 3.81 | 188.28 | 283 |
| 1 | 1 | 37 | 147 | 102 | 31.10 | 6.09 | 1.23 | 1.43 | 4.07 | 5.58 | 3.83 | 7.45 | 32.26 | 6.0 | 1.85 | 71.63 | 284 |
| 1 | 1 | 56 | 131 | 90 | 24.90 | 4.17 | 0.98 | 1.23 | 2.4 | 5.19 | 4.67 | 7.35 | 69.30 | 5.7 | 1.70 | 86.98 | 285 |
| 1 | 1 | 53 | 110 | 60 | 33.20 | 3.46 | 2.03 | 1.18 | 1.56 | 5.57 | 5.96 | 12.35 | 43.19 | 5.7 | 3.06 | 119.32 | 267 |
| 1 | 2 | 27 | 120 | 80 | 24.30 | 4.11 | 1.24 | 1.17 | 2.46 | 4.24 | 7.24 | 15.26 | 25.6 | 5.1 | 2.88 | 412.43 | 134 |
| 1 | 2 | 27 | 115 | 75 | 28.30 | 4.72 | 2.48 | 1.34 | 3.07 | 4.59 | 6.43 | 12.19 | 73.9 | 5.8 | 2.49 | 223.67 | 147 |
| 1 | 2 | 25 | 100 | 60 | 20.00 | 3.32 | 0.65 | 1.12 | 1.95 | 4.76 | 3.67 | 16.2 | 52.2 | 5.5 | 3.43 | 257.14 | 147 |
| 1 | 2 | 26 | 110 | 70 | 20.70 | 3.70 | 0.93 | 1.51 | 1.98 | 4.72 | 5.27 | 7.94 | 40.08 | 5.4 | 1.67 | 130.16 | 148 |
| 1 | 2 | 28 | 112 | 69 | 19.80 | 3.92 | 1.42 | 1.12 | 2.40 | 4.07 | 3.89 | 6.85 | 23.86 | 5.8 | 1.24 | 240.35 | 156 |
| 1 | 2 | 26 | 120 | 80 | 27.30 | 4.17 | 1.07 | 1.15 | 2.46 | 5.35 | 5.78 | 18.96 | 60.96 | 5.4 | 4.51 | 204.97 | 157 |
| 1 | 2 | 26 | 110 | 80 | 18.40 | 3.83 | 1.37 | 1.40 | 2.18 | 4.22 | 4.9 | 6.17 | 45.89 | 5.7 | 1.16 | 171.39 | 159 |
| 1 | 2 | 28 | 104 | 75 | 20.50 | 3.83 | 0.51 | 1.49 | 2.06 | 5.25 | 4.96 | 16.46 | 20.55 | 5.2 | 3.84 | 188.11 | 169 |
| 1 | 2 | 27 | 120 | 80 | 19.50 | 4.45 | 0.86 | 1.76 | 2.54 | 4.6 | 5.13 | 6.23 | 54.55 | 5.4 | 1.27 | 113.27 | 169 |
| 1 | 2 | 42 | 120 | 80 | 26.50 | 4.14 | 1.28 | 1.16 | 2.66 | 5.38 | 6.03 | 19.44 | 106.7 | 6.0 | 4.65 | 206.81 | 172 |
| 1 | 2 | 26 | 116 | 78 | 20.90 | 4.49 | 1.39 | 1.50 | 2.57 | 5.05 | 5.66 | 8.43 | 24.2 | 5.9 | 1.79 | 108.77 | 172 |
| 1 | 2 | 28 | 107 | 71 | 21.50 | 4.49 | 0.95 | 1.67 | 2.53 | 5.5 | 6.67 | 12.59 | 58.9 | 4.9 | 3.08 | 125.90 | 174 |
| 1 | 2 | 57 | 97 | 76 | 20.20 | 7.65 | 1.20 | 1.62 | 5.15 | 4.86 | 6.83 | 6.53 | 87.6 | 6.0 | 1.41 | 96.03 | 174 |
| 1 | 2 | 59 | 112 | 65 | 22.30 | 5.52 | 0.96 | 1.47 | 3.49 | 5.33 | 5.03 | 7.49 | 31.07 | 6.2 | 1.77 | 81.86 | 178 |
| 1 | 2 | 26 | 115 | 74 | 23.50 | 3.45 | 0.92 | 0.95 | 2.15 | 4.4 | 5.15 | 15.36 | 39.75 | 5.4 | 3.00 | 341.33 | 181 |
| 1 | 2 | 25 | 130 | 80 | 22.90 | 4.81 | 1.33 | 1.21 | 2.97 | 4.69 | 6.5 | 10.58 | 21.5 | 5.6 | 2.21 | 177.82 | 181 |
| 1 | 2 | 26 | 100 | 68 | 22.40 | 4.77 | 1.27 | 1.76 | 2.65 | 5.09 | 5.28 | 9.3 | 30.19 | 5.5 | 2.10 | 116.98 | 182 |
| 1 | 2 | 26 | 115 | 58 | 21.50 | 3.98 | 0.61 | 1.35 | 2.33 | 5.18 | 4.11 | 6.23 | 21.69 | 5.4 | 1.43 | 74.17 | 183 |
| 1 | 2 | 28 | 108 | 65 | 21.90 | 3.37 | 0.85 | 1.35 | 1.89 | 4.78 | 4.9 | 11.06 | 26.11 | 5.4 | 2.64 | 172.81 | 184 |
| 1 | 2 | 27 | 111 | 74 | 20.50 | 4.41 | 0.82 | 1.57 | 2.65 | 4.98 | 5.52 | 13 | 52.9 | 6.0 | 2.98 | 175.68 | 184 |
| 1 | 2 | 58 | 108 | 70 | 21.80 | 6.21 | 2.00 | 1.29 | 4.05 | 5.19 | 5.69 | 6.47 | 33.75 | 5.7 | 1.25 | 76.57 | 184 |
| 1 | 2 | 27 | 110 | 80 | 20.50 | 3.61 | 0.53 | 1.71 | 1.82 | 4.77 | 2.77 | 8.62 | 13.73 | 5.7 | 1.83 | 135.75 | 185 |
| 1 | 2 | 27 | 125 | 80 | 25.50 | 3.20 | 1.27 | 0.99 | 1.98 | 3.98 | 4.13 | 12.55 | 53.24 | 5.7 | 2.64 | 522.92 | 185 |
| 1 | 2 | 48 | 106 | 62 | 24.00 | 3.92 | 1.36 | 0.83 | 2.72 | 4.98 | 6.61 | 6.51 | 47.7 | 5.4 | 1.44 | 87.97 | 187 |
| 1 | 2 | 53 | 124 | 78 | 24.00 | 5.42 | 1.70 | 1.30 | 3.60 | 4.77 | 6.13 | 6.99 | 39.31 | 5.9 | 1.48 | 110.08 | 187 |
| 1 | 2 | 25 | 117 | 78 | 27.10 | 4.28 | 0.65 | 1.52 | 2.49 | 4.84 | 6 | 10.27 | 49.06 | 5.8 | 2.21 | 153.28 | 188 |
| 1 | 2 | 30 | 107 | 66 | 29.30 | 3.32 | 0.76 | 0.98 | 2.08 | 5.54 | 6.74 | 14.25 | 44.89 | 5.4 | 3.51 | 139.71 | 188 |
| 1 | 2 | 47 | 99 | 60 | 21.50 | 4.83 | 1.19 | 1.43 | 3.03 | 4.6 | 5.36 | 10.32 | 26.85 | 5.9 | 2.11 | 187.64 | 189 |
| 1 | 2 | 47 | 116 | 74 | 27.90 | 4.72 | 0.61 | 1.38 | 3.01 | 4.84 | 4.67 | 8.74 | 27.86 | 5.5 | 2.04 | 130.45 | 189 |
| 1 | 2 | 40 | 113 | 76 | 22.90 | 4.67 | 1.74 | 1.11 | 3.15 | 4.84 | 4.54 | 5.1 | 47.68 | 5.6 | 1.10 | 76.12 | 189 |
| 1 | 2 | 52 | 116 | 79 | 29.70 | 4.89 | 3.00 | 1.40 | 2.97 | 5.43 | 6.7 | 11.62 | 43.2 | 5.6 | 2.47 | 120.41 | 190 |
| 1 | 2 | 52 | 135 | 87 | 23.90 | 5.91 | 1.17 | 1.72 | 3.73 | 4.77 | 4.98 | 8.43 | 37.8 | 5.4 | 1.89 | 132.76 | 193 |
| 1 | 2 | 25 | 91 | 60 | 23.50 | 3.56 | 0.73 | 1.18 | 2.17 | 4.62 | 5.04 | 7.61 | 33.39 | 5.6 | 1.62 | 135.89 | 193 |
| 1 | 2 | 62 | 143 | 84 | 23.50 | 5.34 | 1.66 | 1.47 | 3.32 | 4.99 | 5.59 | 5.9 | 35.1 | 5.7 | 1.25 | 79.19 | 196 |
| 1 | 2 | 25 | 124 | 88 | 23.90 | 5.43 | 1.14 | 2.00 | 2.90 | 5.36 | 5.97 | 15.14 | 76.79 | 5.5 | 3.61 | 162.80 | 196 |
| 1 | 2 | 58 | 143 | 78 | 26.00 | 5.87 | 3.41 | 1.38 | 3.61 | 5.31 | 5.74 | 17.05 | 76.65 | 5.9 | 4.02 | 188.40 | 196 |
| 1 | 2 | 52 | 126 | 73 | 31.60 | 4.16 | 1.23 | 0.94 | 2.84 | 5.09 | 6.63 | 13.27 | 42.81 | 5.4 | 3.00 | 166.92 | 198 |
| 1 | 2 | 33 | 90 | 60 | 17.00 | 3.47 | 0.53 | 1.52 | 1.85 | 4.94 | 4.85 | 9.63 | 32.97 | 5.8 | 2.11 | 133.75 | 200 |
| 1 | 2 | 37 | 108 | 75 | 35.00 | 4.34 | 2.16 | 0.90 | 2.86 | 5.43 | 6.11 | 17.03 | 56.69 | 6.3 | 4.11 | 176.48 | 202 |
| 1 | 2 | 67 | 110 | 71 | 25.50 | 4.16 | 1.31 | 1.33 | 2.55 | 5.26 | 3.78 | 6.68 | 12.78 | 5.5 | 1.56 | 75.91 | 504 |
| 1 | 2 | 30 | 120 | 69 | 21.10 | 4.08 | 0.57 | 1.27 | 2.47 | 4.73 | 5.99 | 9.33 | 55.05 | 5.4 | 1.96 | 151.71 | 207 |
| 1 | 2 | 31 | 138 | 77 | 28.60 | 3.49 | 1.04 | 1.00 | 2.17 | 5.43 | 6.81 | 17.79 | 86.6 | 5.7 | 4.29 | 184.35 | 207 |
| 1 | 2 | 50 | 115 | 66 | 28.10 | 5.47 | 2.32 | 1.35 | 3.41 | 4.63 | 6.73 | 13.45 | 62.26 | 5.4 | 2.77 | 238.05 | 209 |
| 1 | 2 | 26 | 120 | 80 | 21.30 | 3.65 | 2.58 | 1.91 | 2.31 | 4.98 | 6.08 | 9.72 | 73.36 | 5.4 | 2.15 | 131.35 | 210 |
| 1 | 2 | 25 | 120 | 76 | 19.20 | 3.65 | 0.67 | 1.43 | 1.87 | 4.69 | 5.27 | 10.29 | 25.98 | 5.8 | 2.05 | 172.94 | 211 |
| 1 | 2 | 26 | 109 | 70 | 22.70 | 3.37 | 0.48 | 1.50 | 1.74 | 4.88 | 4.65 | 18.34 | 30.93 | 5.7 | 3.98 | 265.80 | 211 |
| 1 | 2 | 27 | 120 | 69 | 19.10 | 4.13 | 0.70 | 1.41 | 2.44 | 4.53 | 5.19 | 11.04 | 61.39 | 6.0 | 2.22 | 214.37 | 211 |
| 1 | 2 | 27 | 111 | 75 | 19.70 | 3.61 | 0.86 | 1.22 | 2.14 | 4.38 | 3.27 | 12.29 | 59.54 | 5.9 | 2.39 | 279.32 | 212 |
| 1 | 2 | 26 | 112 | 63 | 19.20 | 3.80 | 0.49 | 1.54 | 2.01 | 5.21 | 5.6 | 7.8 | 31.08 | 5.7 | 1.81 | 91.23 | 212 |
| 1 | 2 | 51 | 99 | 59 | 23.30 | 4.31 | 1.09 | 1.14 | 2.71 | 4.65 | 5.55 | 8.79 | 32.67 | 5.9 | 1.82 | 152.87 | 213 |
| 1 | 2 | 24 | 110 | 60 | 23.30 | 4.30 | 0.84 | 1.10 | 2.75 | 4.82 | 6.42 | 9.42 | 46.68 | 5.3 | 2.34 | 142.73 | 215 |
| 1 | 2 | 27 | 102 | 64 | 21.70 | 4.70 | 0.79 | 1.30 | 3.07 | 4.95 | 4.27 | 16.08 | 28.85 | 5.7 | 3.54 | 221.79 | 215 |
| 1 | 2 | 27 | 106 | 85 | 18.40 | 4.29 | 0.93 | 1.33 | 2.42 | 4.97 | 7.22 | 16.17 | 83.38 | 5.8 | 3.33 | 220.00 | 217 |
| 1 | 2 | 53 | 124 | 82 | 23.50 | 6.06 | 1.35 | 1.48 | 3.67 | 5.43 | 6.28 | 8.93 | 67.26 | 5.9 | 2.16 | 92.54 | 217 |
| 1 | 2 | 26 | 110 | 70 | 22.90 | 4.11 | 0.60 | 1.71 | 2.26 | 5.12 | 4.31 | 9.88 | 25 | 4.9 | 2.25 | 121.98 | 219 |
| 1 | 2 | 24 | 117 | 72 | 20.90 | 5.37 | 1.19 | 1.76 | 3.21 | 4.83 | 4.4 | 7.01 | 21.99 | 5.1 | 1.53 | 105.41 | 219 |
| 1 | 2 | 26 | 110 | 69 | 19.10 | 3.83 | 0.82 | 1.40 | 1.96 | 5.09 | 3.4 | 5.73 | 16.13 | 5.4 | 1.40 | 72.08 | 219 |
| 1 | 2 | 58 | 147 | 89 | 43.10 | 6.20 | 2.98 | 1.14 | 4.15 | 5.14 | 4.41 | 17 | 32.29 | 6.1 | 3.88 | 207.32 | 221 |
| 1 | 2 | 27 | 120 | 75 | 19.80 | 3.70 | 0.58 | 1.62 | 1.81 | 4.82 | 5.87 | 8.09 | 39.34 | 5.7 | 1.73 | 122.58 | 222 |
| 1 | 2 | 54 | 130 | 80 | 21.60 | 5.58 | 1.82 | 1.30 | 3.64 | 5.28 | 4.85 | 7.2 | 16.81 | 5.3 | 1.69 | 80.90 | 223 |
| 1 | 2 | 56 | 106 | 69 | 23.80 | 6.55 | 1.26 | 1.62 | 3.94 | 4.58 | 6.35 | 5.03 | 35.65 | 5.8 | 1.02 | 93.15 | 224 |
| 1 | 2 | 27 | 110 | 70 | 20.80 | 5.79 | 0.90 | 2.00 | 3.34 | 4.54 | 5.18 | 7.62 | 66.71 | 5.7 | 1.54 | 146.54 | 224 |
| 1 | 2 | 23 | 107 | 66 | 16.90 | 3.43 | 0.55 | 1.30 | 1.91 | 4.06 | 3.73 | 7.42 | 59.61 | 5.8 | 1.67 | 265.00 | 224 |
| 1 | 2 | 42 | 120 | 80 | 27.80 | 3.86 | 1.06 | 0.81 | 2.68 | 5.12 | 6.36 | 13.11 | 96.64 | 5.7 | 2.98 | 161.85 | 224 |
| 1 | 2 | 32 | 97 | 58 | 27.40 | 4.92 | 5.05 | 0.79 | 3.06 | 4.47 | 4.01 | 7.82 | 37.97 | 5.6 | 1.60 | 161.24 | 226 |
| 1 | 2 | 24 | 108 | 68 | 18.20 | 4.10 | 0.88 | 1.92 | 1.79 | 4.2 | 4.08 | 8.26 | 23.91 | 5.2 | 1.54 | 236.00 | 227 |
| 1 | 2 | 33 | 118 | 76 | 25.13 | 4.33 | 1.02 | 1.42 | 2.43 | 5.19 | 6.96 | 9.13 | 65.11 | 5.9 | 2.11 | 108.05 | 227 |
| 1 | 2 | 41 | 108 | 70 | 26.80 | 4.55 | 1.08 | 1.17 | 2.96 | 4.93 | 4.11 | 12.76 | 45.49 | 5.5 | 2.80 | 178.46 | 227 |
| 1 | 2 | 54 | 130 | 80 | 26.20 | 5.45 | 1.06 | 1.63 | 3.59 | 4.43 | 4.12 | 7.46 | 59.53 | 6.3 | 1.70 | 160.43 | 228 |
| 1 | 2 | 52 | 110 | 69 | 24.50 | 6.86 | 3.03 | 1.38 | 4.87 | 4.83 | 4.19 | 7.06 | 55.94 | 5.8 | 1.52 | 106.17 | 228 |
| 1 | 2 | 49 | 98 | 64 | 25.50 | 4.10 | 0.72 | 1.15 | 2.66 | 4.76 | 6.05 | 8.55 | 63.51 | 5.6 | 1.81 | 135.71 | 229 |
| 1 | 2 | 48 | 139 | 89 | 30.60 | 3.66 | 1.16 | 1.28 | 2.03 | 4.69 | 4.25 | 12.4 | 35.87 | 5.5 | 2.58 | 208.40 | 229 |
| 1 | 2 | 58 | 119 | 88 | 26.30 | 5.87 | 0.65 | 2.13 | 3.52 | 4.65 | 4.23 | 17.91 | 60.23 | 4.8 | 3.70 | 311.48 | 229 |
| 1 | 2 | 44 | 89 | 55 | 24.50 | 4.17 | 2.25 | 0.87 | 2.80 | 4.91 | 6.85 | 7.6 | 70.34 | 5.9 | 1.66 | 107.80 | 230 |
| 1 | 2 | 61 | 138 | 79 | 24.50 | 4.45 | 1.09 | 1.26 | 2.88 | 4.81 | 4.6 | 6.56 | 20.49 | 5.1 | 1.49 | 100.15 | 230 |
| 1 | 2 | 54 | 133 | 83 | 30.10 | 4.29 | 1.30 | 1.28 | 2.72 | 5.27 | 4.4 | 17.31 | 55.57 | 5.6 | 4.05 | 195.59 | 230 |
| 1 | 2 | 42 | 120 | 80 | 22.60 | 4.59 | 0.58 | 1.07 | 2.89 | 5.46 | 6.24 | 6.12 | 48.72 | 5.3 | 1.44 | 62.45 | 231 |
| 1 | 2 | 27 | 109 | 66 | 23.50 | 4.10 | 0.60 | 1.24 | 2.67 | 5.18 | 6.79 | 6.07 | 56.87 | 5.6 | 1.40 | 72.26 | 231 |
| 1 | 2 | 50 | 101 | 55 | 22.10 | 5.03 | 0.69 | 1.72 | 2.83 | 5.45 | 5.55 | 8.53 | 15.17 | 5.3 | 2.07 | 87.49 | 232 |
| 1 | 2 | 39 | 111 | 87 | 21.20 | 4.87 | 1.28 | 1.42 | 2.96 | 4.54 | 4.96 | 10.01 | 29.18 | 5.4 | 2.02 | 192.50 | 232 |
| 1 | 2 | 53 | 129 | 65 | 23.10 | 4.62 | 1.38 | 1.22 | 3.02 | 5.29 | 6.04 | 7.53 | 29.8 | 5.4 | 1.77 | 84.13 | 233 |
| 1 | 2 | 45 | 107 | 68 | 24.70 | 5.03 | 1.03 | 1.32 | 3.16 | 5.28 | 4.01 | 6.77 | 40.02 | 4.7 | 1.59 | 76.07 | 234 |
| 1 | 2 | 26 | 110 | 70 | 21.90 | 4.01 | 0.84 | 1.17 | 2.56 | 4.97 | 4.21 | 6.95 | 17.27 | 5.2 | 1.60 | 94.56 | 234 |
| 1 | 2 | 27 | 125 | 78 | 25.20 | 4.73 | 0.94 | 1.18 | 3.11 | 4.51 | 5.5 | 10.28 | 87.57 | 5.5 | 2.06 | 203.56 | 234 |
| 1 | 2 | 57 | 117 | 76 | 25.80 | 4.49 | 2.20 | 1.09 | 2.91 | 5.1 | 6.34 | 11.02 | 79.97 | 5.3 | 2.50 | 137.75 | 234 |
| 1 | 2 | 55 | 130 | 80 | 27.20 | 4.72 | 2.19 | 1.15 | 2.97 | 5.35 | 5.39 | 15.4 | 78.39 | 5.4 | 3.66 | 166.49 | 235 |
| 1 | 2 | 50 | 120 | 82 | 29.50 | 6.79 | 1.50 | 1.26 | 4.70 | 5.37 | 5.81 | 11.75 | 23 | 5.6 | 2.80 | 125.67 | 235 |
| 1 | 2 | 57 | 127 | 81 | 24.20 | 6.40 | 0.66 | 1.94 | 4.01 | 4.69 | 5.14 | 6.13 | 26.62 | 5.4 | 1.28 | 103.03 | 236 |
| 1 | 2 | 52 | 128 | 78 | 22.20 | 5.89 | 0.91 | 1.53 | 3.82 | 4.35 | 3.76 | 5.78 | 37.01 | 5.7 | 1.12 | 136.00 | 236 |
| 1 | 2 | 27 | 104 | 65 | 22.30 | 4.53 | 1.37 | 1.60 | 2.39 | 4.55 | 4.15 | 7.14 | 27.19 | 5.1 | 1.44 | 136.00 | 236 |
| 1 | 2 | 26 | 92 | 67 | 19.40 | 3.59 | 0.96 | 1.09 | 2.20 | 4.37 | 3.75 | 7.24 | 26.15 | 5.0 | 1.75 | 166.44 | 236 |
| 1 | 2 | 54 | 111 | 62 | 18.90 | 4.98 | 0.62 | 2.03 | 2.68 | 5.17 | 5.59 | 6.65 | 14.07 | 5.4 | 1.53 | 79.64 | 237 |
| 1 | 2 | 51 | 112 | 71 | 26.80 | 3.74 | 0.85 | 0.97 | 2.35 | 5.13 | 6.94 | 9.24 | 87.2 | 4.8 | 2.11 | 113.37 | 237 |
| 1 | 2 | 54 | 109 | 74 | 21.80 | 5.48 | 2.24 | 1.44 | 3.30 | 4.95 | 5.12 | 11.25 | 39.89 | 5.1 | 2.48 | 155.17 | 237 |
| 1 | 2 | 24 | 108 | 70 | 18.20 | 6.54 | 1.05 | 1.69 | 4.15 | 4.52 | 4.45 | 18.05 | 33.01 | 5.1 | 3.63 | 353.92 | 237 |
| 1 | 2 | 50 | 121 | 74 | 24.10 | 5.33 | 2.79 | 1.19 | 3.33 | 5.58 | 5.81 | 9.42 | 104.5 | 5.1 | 2.02 | 90.58 | 238 |
| 1 | 2 | 52 | 120 | 68 | 22.70 | 6.26 | 0.80 | 1.99 | 3.94 | 4.85 | 4.8 | 8.9 | 36.14 | 5.0 | 2.09 | 131.85 | 238 |
| 1 | 2 | 48 | 131 | 80 | 24.40 | 5.31 | 0.85 | 1.74 | 3.24 | 4.67 | 5.08 | 6.02 | 28.89 | 5.3 | 1.25 | 102.91 | 238 |
| 1 | 2 | 62 | 120 | 67 | 22.00 | 6.79 | 1.12 | 1.79 | 4.44 | 4.62 | 6.24 | 7.15 | 38.86 | 5.2 | 1.47 | 127.68 | 238 |
| 1 | 2 | 57 | 117 | 68 | 26.90 | 7.47 | 1.66 | 1.46 | 5.13 | 4.77 | 6.89 | 10.19 | 50.64 | 5.1 | 2.16 | 160.47 | 238 |
| 1 | 2 | 52 | 123 | 72 | 26.00 | 4.56 | 0.74 | 1.23 | 2.79 | 5.33 | 7.19 | 19.08 | 102.9 | 4.8 | 4.52 | 208.52 | 238 |
| 1 | 2 | 52 | 130 | 80 | 20.80 | 5.77 | 1.29 | 1.63 | 3.57 | 5.24 | 5.08 | 6.58 | 11.8 | 4.9 | 1.53 | 75.63 | 238 |
| 1 | 2 | 48 | 127 | 77 | 29.10 | 6.03 | 2.23 | 1.42 | 3.76 | 4.78 | 4.48 | 8.49 | 31.66 | 5.2 | 1.94 | 132.66 | 238 |
| 1 | 2 | 33 | 128 | 78 | 20.70 | 3.50 | 0.61 | 1.29 | 2.01 | 4.96 | 5.24 | 9.22 | 45.56 | 4.9 | 2.25 | 126.30 | 238 |
| 1 | 2 | 53 | 111 | 67 | 27.50 | 4.50 | 1.21 | 1.36 | 2.61 | 4.99 | 5.6 | 8.76 | 18.97 | 5.0 | 1.94 | 117.58 | 238 |
| 1 | 2 | 62 | 121 | 74 | 28.30 | 6.61 | 1.02 | 1.57 | 4.22 | 4.76 | 3.35 | 5.9 | 44.25 | 5.0 | 1.31 | 93.65 | 238 |
| 1 | 2 | 42 | 118 | 76 | 27.70 | 4.22 | 0.80 | 1.21 | 2.54 | 5.11 | 5.08 | 8.75 | 23.26 | 5.6 | 1.99 | 108.70 | 239 |
| 1 | 2 | 52 | 149 | 70 | 27.90 | 5.69 | 0.94 | 1.31 | 3.82 | 5.28 | 7.13 | 12.78 | 47.82 | 4.7 | 3.00 | 143.60 | 239 |
| 1 | 2 | 65 | 159 | 83 | 26.00 | 6.94 | 1.29 | 1.65 | 4.71 | 5.25 | 5.81 | 6.5 | 25.45 | 5.1 | 1.52 | 74.29 | 240 |
| 1 | 2 | 57 | 123 | 77 | 23.30 | 3.79 | 0.43 | 1.80 | 2.04 | 4.97 | 7.08 | 5.87 | 19.68 | 4.8 | 1.30 | 79.86 | 240 |
| 1 | 2 | 26 | 112 | 71 | 27.30 | 4.11 | 0.67 | 1.51 | 2.25 | 4.98 | 5.91 | 8.69 | 104.4 | 5.3 | 1.92 | 117.43 | 242 |
| 1 | 2 | 47 | 115 | 64 | 26.60 | 4.61 | 0.99 | 1.29 | 2.94 | 5.13 | 5.29 | 17.6 | 73.72 | 5.3 | 4.01 | 215.95 | 242 |
| 1 | 2 | 39 | 102 | 64 | 24.30 | 3.67 | 0.87 | 1.20 | 2.23 | 4.64 | 5.56 | 16.17 | 27.61 | 5.2 | 3.57 | 283.68 | 242 |
| 1 | 2 | 49 | 105 | 71 | 24.50 | 4.62 | 1.07 | 1.21 | 3.17 | 5.11 | 5.27 | 5.82 | 25.78 | 5.8 | 1.32 | 72.30 | 242 |
| 1 | 2 | 57 | 132 | 67 | 24.30 | 4.24 | 0.99 | 1.08 | 2.87 | 5.3 | 4.86 | 8.46 | 20.16 | 5.4 | 1.99 | 94.00 | 243 |
| 1 | 2 | 31 | 132 | 76 | 30.10 | 3.43 | 0.53 | 1.65 | 1.80 | 5.55 | 6.57 | 12.32 | 76.99 | 6.0 | 3.04 | 120.20 | 244 |
| 1 | 2 | 50 | 123 | 76 | 25.20 | 5.17 | 1.17 | 1.55 | 3.41 | 4.62 | 5.03 | 5.69 | 12.2 | 5.1 | 1.17 | 101.61 | 244 |
| 1 | 2 | 46 | 127 | 73 | 25.70 | 4.12 | 1.58 | 1.08 | 2.68 | 5.06 | 5.98 | 7.3 | 40.36 | 5.8 | 1.76 | 93.59 | 245 |
| 1 | 2 | 48 | 146 | 65 | 21.70 | 4.30 | 0.55 | 1.92 | 2.32 | 5.39 | 3.97 | 8.84 | 15.12 | 5.8 | 2.12 | 93.54 | 245 |
| 1 | 2 | 39 | 126 | 82 | 22.30 | 4.66 | 0.78 | 1.68 | 2.84 | 4.98 | 5.91 | 8.13 | 66.13 | 5.3 | 1.80 | 109.86 | 245 |
| 1 | 2 | 53 | 130 | 72 | 26.70 | 4.38 | 0.59 | 1.64 | 2.71 | 4.92 | 2.95 | 7.63 | 47.54 | 6.1 | 1.67 | 107.46 | 246 |
| 1 | 2 | 50 | 129 | 89 | 26.40 | 6.04 | 0.73 | 1.68 | 3.89 | 5.03 | 5.92 | 7.9 | 109.3 | 4.7 | 1.77 | 103.27 | 246 |
| 1 | 2 | 52 | 132 | 79 | 22.80 | 6.16 | 2.74 | 1.45 | 3.91 | 4.92 | 6.38 | 10.82 | 97.8 | 5.9 | 2.37 | 152.39 | 246 |
| 1 | 2 | 57 | 113 | 63 | 19.80 | 4.75 | 0.64 | 2.08 | 2.56 | 4.73 | 5.56 | 6.54 | 37.75 | 5.3 | 1.37 | 106.34 | 247 |
| 1 | 2 | 27 | 110 | 72 | 20.50 | 3.13 | 0.69 | 1.28 | 1.66 | 4.29 | 5.34 | 9.95 | 42.59 | 6.0 | 1.90 | 251.90 | 248 |
| 1 | 2 | 49 | 130 | 80 | 24.30 | 3.36 | 0.74 | 1.78 | 2.16 | 5.09 | 5.85 | 14.4 | 101.7 | 5.4 | 3.26 | 181.13 | 249 |
| 1 | 2 | 58 | 117 | 72 | 24.50 | 4.54 | 0.83 | 1.53 | 2.67 | 5 | 5.05 | 5.02 | 17.87 | 5.4 | 1.12 | 66.93 | 249 |
| 1 | 2 | 37 | 107 | 62 | 21.20 | 3.20 | 0.58 | 1.55 | 1.60 | 4.78 | 4.92 | 7.61 | 26.44 | 5.4 | 1.56 | 118.91 | 250 |
| 1 | 2 | 54 | 143 | 93 | 25.50 | 5.22 | 0.98 | 1.32 | 3.43 | 5.18 | 6.78 | 6.12 | 61.92 | 5.5 | 1.41 | 72.86 | 250 |
| 1 | 2 | 48 | 131 | 74 | 34.00 | 3.42 | 1.22 | 1.03 | 2.24 | 4.78 | 5.37 | 11.62 | 50.31 | 5.4 | 2.80 | 181.56 | 251 |
| 1 | 2 | 53 | 150 | 90 | 30.90 | 3.50 | 0.56 | 1.10 | 2.15 | 4.58 | 5.38 | 6.5 | 41.87 | 5.8 | 1.32 | 120.37 | 253 |
| 1 | 2 | 35 | 136 | 87 | 26.40 | 3.10 | 0.60 | 1.10 | 1.84 | 5.22 | 5.88 | 10.71 | 82.9 | 6.1 | 2.48 | 124.53 | 253 |
| 1 | 2 | 48 | 124 | 70 | 25.60 | 4.38 | 0.69 | 1.45 | 2.64 | 5.13 | 4.48 | 9.46 | 28.06 | 5.7 | 2.16 | 116.07 | 254 |
| 1 | 2 | 46 | 108 | 64 | 26.70 | 4.20 | 1.17 | 1.19 | 2.70 | 4.9 | 6.73 | 10.71 | 67.31 | 5.7 | 2.33 | 153.00 | 255 |
| 1 | 2 | 48 | 102 | 64 | 26.10 | 7.19 | 1.45 | 1.65 | 4.95 | 5.05 | 6.87 | 12.15 | 59.1 | 5.3 | 2.73 | 156.77 | 255 |
| 1 | 2 | 34 | 110 | 76 | 28.90 | 4.47 | 1.11 | 1.21 | 2.95 | 5.09 | 7 | 9.3 | 90.58 | 5.5 | 2.10 | 116.98 | 255 |
| 1 | 2 | 41 | 129 | 92 | 29.20 | 3.85 | 1.10 | 1.02 | 2.35 | 4.93 | 6.1 | 11.05 | 57.9 | 5.6 | 2.42 | 154.55 | 256 |
| 1 | 2 | 43 | 118 | 76 | 26.70 | 5.43 | 0.95 | 1.27 | 3.67 | 5.19 | 6.07 | 8.31 | 43.91 | 5.6 | 2.02 | 98.34 | 256 |
| 1 | 2 | 50 | 115 | 71 | 26.60 | 5.16 | 0.47 | 1.75 | 3.06 | 4.74 | 4.66 | 6.4 | 24.63 | 5.9 | 1.43 | 103.23 | 257 |
| 1 | 2 | 35 | 117 | 83 | 24.77 | 3.72 | 0.52 | 1.64 | 2.11 | 4.72 | 5.09 | 7.32 | 53.23 | 5.5 | 1.54 | 120.00 | 257 |
| 1 | 2 | 64 | 140 | 85 | 25.48 | 6.23 | 1.72 | 1.33 | 4.08 | 5.38 | 5.18 | 10.53 | 22.07 | 5.1 | 2.05 | 112.02 | 257 |
| 1 | 2 | 41 | 120 | 71 | 24.65 | 5.38 | 1.12 | 1.43 | 3.19 | 5.01 | 5.07 | 13.51 | 86.73 | 5.5 | 3.01 | 178.94 | 257 |
| 1 | 2 | 50 | 119 | 69 | 24.32 | 5.36 | 0.79 | 1.60 | 3.26 | 4.86 | 6.2 | 9.51 | 74.64 | 5.7 | 2.05 | 139.85 | 261 |
| 1 | 2 | 42 | 112 | 75 | 23.66 | 5.12 | 0.92 | 1.53 | 3.13 | 4.69 | 3.88 | 10.01 | 25.43 | 5.7 | 2.09 | 168.24 | 361 |
| 1 | 2 | 63 | 132 | 86 | 23.81 | 7.23 | 2.24 | 1.70 | 4.59 | 5.06 | 5.67 | 7.5 | 45.79 | 6.1 | 1.69 | 96.15 | 261 |
| 1 | 2 | 47 | 109 | 68 | 23.52 | 4.93 | 1.24 | 1.52 | 2.96 | 5.5 | 7.06 | 6.75 | 27.32 | 5.8 | 1.65 | 67.50 | 261 |
| 1 | 2 | 50 | 107 | 62 | 24.09 | 3.49 | 2.65 | 1.79 | 2.74 | 4.65 | 5.97 | 10.83 | 85.49 | 5.4 | 2.24 | 188.35 | 262 |
| 1 | 2 | 42 | 104 | 68 | 19.81 | 4.67 | 2.40 | 1.24 | 2.68 | 5.3 | 7.12 | 13.38 | 63.67 | 5.3 | 3.15 | 148.67 | 262 |
| 1 | 2 | 47 | 112 | 70 | 21.08 | 5.13 | 1.00 | 1.34 | 3.06 | 4.37 | 4.63 | 6.18 | 34.88 | 5.8 | 1.20 | 142.07 | 263 |
| 1 | 2 | 46 | 150 | 98 | 21.50 | 4.61 | 0.56 | 2.16 | 2.26 | 4.75 | 6.81 | 6.15 | 38.89 | 5.8 | 1.30 | 98.40 | 263 |
| 1 | 2 | 30 | 118 | 78 | 23.91 | 5.36 | 1.16 | 1.56 | 3.08 | 4.88 | 6.43 | 7.62 | 34.46 | 5.7 | 1.65 | 110.43 | 263 |
| 1 | 2 | 40 | 116 | 72 | 20.99 | 4.27 | 1.52 | 1.57 | 2.25 | 4.88 | 5.72 | 5.02 | 59.44 | 5.8 | 1.09 | 72.75 | 263 |
| 1 | 2 | 39 | 141 | 84 | 24.33 | 4.34 | 0.65 | 1.48 | 2.37 | 4.59 | 6.23 | 7.02 | 95.31 | 5.6 | 1.48 | 128.81 | 265 |
| 1 | 2 | 35 | 111 | 72 | 25.63 | 4.43 | 1.00 | 1.17 | 2.58 | 4.62 | 4.36 | 11.19 | 88.3 | 5.4 | 2.30 | 199.82 | 266 |
| 1 | 2 | 32 | 132 | 76 | 22.97 | 4.78 | 0.90 | 1.31 | 2.86 | 4.75 | 5.76 | 7.02 | 29.69 | 5.7 | 1.43 | 112.32 | 266 |
| 1 | 2 | 58 | 130 | 70 | 20.50 | 5.56 | 2.62 | 1.37 | 2.65 | 5.14 | 6.54 | 4.84 | 38.68 | 5.5 | 1.10 | 59.02 | 267 |
| 1 | 2 | 57 | 114 | 65 | 25.10 | 5.16 | 1.17 | 1.43 | 2.81 | 4.88 | 4.04 | 8.73 | 11.26 | 5.4 | 1.89 | 126.52 | 267 |
| 1 | 2 | 28 | 126 | 80 | 26.80 | 4.04 | 0.33 | 1.7 | 1.71 | 4.9 | 5.85 | 8.54 | 19.77 | 5.2 | 1.86 | 122.00 | 267 |
| 1 | 2 | 54 | 130 | 85 | 29.20 | 4.46 | 3.23 | 1.11 | 1.92 | 5.16 | 7.07 | 11.22 | 37.97 | 5.6 | 2.57 | 135.18 | 268 |
| 1 | 2 | 68 | 125 | 75 | 21.30 | 5.42 | 1.37 | 1.37 | 2.76 | 5.25 | 6.6 | 11.94 | 55.54 | 5.4 | 2.79 | 136.46 | 268 |
| 1 | 2 | 49 | 130 | 80 | 23.40 | 5.17 | 0.48 | 2.13 | 2.22 | 5.53 | 7.16 | 5.02 | 15.68 | 5.4 | 1.23 | 49.46 | 268 |
| 1 | 2 | 61 | 154 | 82 | 23.34 | 6.09 | 0.96 | 1.76 | 3.04 | 5.11 | 5.39 | 11.68 | 68.90 | 5.5 | 2.65 | 145.09 | 268 |
| 1 | 2 | 47 | 132 | 79 | 27.06 | 3.53 | 0.62 | 1.83 | 1.54 | 4.65 | 4.54 | 8.71 | 11.09 | 5.3 | 1.85 | 151.48 | 268 |
| 1 | 2 | 59 | 121 | 77 | 21.48 | 4.76 | 1.91 | 1.97 | 2.14 | 4.88 | 5.9 | 6.21 | 34.28 | 5.4 | 1.35 | 90.00 | 268 |
| 1 | 2 | 52 | 128 | 85 | 28.80 | 4.17 | 0.93 | 1.33 | 2.26 | 4.96 | 4.71 | 7.08 | 25.43 | 5.7 | 1.66 | 96.99 | 269 |
| 1 | 2 | 24 | 105 | 64 | 24.86 | 5.27 | 0.64 | 2 | 2.85 | 4.93 | 5.55 | 6.62 | 19.98 | 4.8 | 1.45 | 92.59 | 269 |
| 1 | 2 | 58 | 121 | 80 | 30.10 | 4.15 | 0.77 | 2.7 | 1.12 | 5.55 | 5.2 | 7.13 | 20.02 | 5.4 | 1.76 | 69.56 | 270 |
| 1 | 2 | 74 | 140 | 91 | 31.30 | 5.54 | 0.81 | 1.55 | 3.46 | 5.47 | 4.59 | 8.31 | 34.39 | 5.9 | 1.89 | 84.37 | 270 |
| 1 | 2 | 51 | 130 | 75 | 26.23 | 4.76 | 0.74 | 2.15 | 2.16 | 5.26 | 5.83 | 6.22 | 36.50 | 5.3 | 1.45 | 70.68 | 270 |
| 1 | 2 | 60 | 119 | 79 | 25.40 | 4.32 | 0.74 | 1.94 | 1.81 | 4.59 | 6.4 | 8.33 | 39.68 | 5.4 | 1.70 | 152.84 | 470 |
| 1 | 2 | 53 | 113 | 75 | 18.70 | 4.16 | 0.4 | 2.41 | 1.45 | 4.68 | 4.87 | 7.31 | 11.50 | 5.4 | 1.63 | 123.90 | 272 |
| 1 | 2 | 27 | 115 | 80 | 20.55 | 4.15 | 0.76 | 1.82 | 1.95 | 5.06 | 4.51 | 11.34 | 55.75 | 5.7 | 2.70 | 145.38 | 272 |
| 1 | 2 | 44 | 125 | 80 | 25.60 | 4.06 | 0.56 | 1.96 | 1.68 | 5.06 | 6.46 | 7.04 | 23.98 | 5.6 | 1.58 | 90.26 | 273 |
| 1 | 2 | 52 | 134 | 75 | 24.80 | 5.52 | 1.42 | 1.55 | 3.3 | 4.74 | 7.07 | 13.09 | 65.22 | 6.1 | 2.76 | 211.13 | 603 |
| 1 | 2 | 56 | 134 | 92 | 26.40 | 4.41 | 0.72 | 1.79 | 1.79 | 5.13 | 6.33 | 8.93 | 45.83 | 4.8 | 2.04 | 109.57 | 273 |
| 1 | 2 | 55 | 133 | 77 | 27.60 | 5.39 | 1.73 | 1.37 | 3.17 | 5.14 | 5.26 | 11.67 | 30.72 | 5.4 | 2.67 | 142.32 | 273 |
| 1 | 2 | 50 | 127 | 81 | 21.10 | 5.09 | 1.38 | 1.56 | 2.69 | 4.87 | 6.22 | 8.38 | 56.74 | 5.5 | 1.81 | 122.34 | 273 |
| 1 | 2 | 41 | 137 | 91 | 27.50 | 3.32 | 0.36 | 1.15 | 1.82 | 5.01 | 4.6 | 12.55 | 67.07 | 5.4 | 2.29 | 166.23 | 274 |
| 1 | 2 | 46 | 140 | 90 | 30.20 | 4.15 | 1.32 | 1.4 | 1.47 | 5.03 | 4.86 | 11.23 | 60.68 | 5.4 | 2.51 | 146.80 | 274 |
| 1 | 2 | 49 | 123 | 75 | 19.50 | 5.01 | 0.65 | 1.87 | 2.66 | 4.46 | 4.82 | 6.22 | 25.72 | 5.4 | 1.23 | 129.58 | 274 |
| 1 | 2 | 71 | 116 | 59 | 24.00 | 5.11 | 1.03 | 1.81 | 2.76 | 5.04 | 7.24 | 5.11 | 29.38 | 5.6 | 1.14 | 66.36 | 274 |
| 1 | 2 | 47 | 110 | 70 | 26.40 | 5.36 | 1.14 | 1.59 | 3.93 | 5.29 | 5.45 | 11.08 | 50.60 | 5.9 | 2.61 | 123.80 | 274 |
| 1 | 2 | 60 | 123 | 67 | 27.50 | 4.86 | 0.8 | 1.49 | 3.56 | 5.56 | 6.24 | 13.27 | 50.08 | 5.7 | 3.23 | 128.83 | 474 |
| 1 | 2 | 56 | 134 | 86 | 24.50 | 4.56 | 1.2 | 1.58 | 2.49 | 4.95 | 6.72 | 7.14 | 32.93 | 5.8 | 1.57 | 98.48 | 275 |
| 1 | 2 | 62 | 115 | 80 | 26.00 | 5.12 | 1.22 | 1.58 | 3.89 | 4.66 | 4.7 | 8.72 | 48.07 | 5.7 | 1.81 | 150.34 | 275 |
| 1 | 2 | 51 | 130 | 75 | 23.20 | 4.78 | 1.3 | 1.45 | 3.36 | 5.18 | 7.07 | 6.24 | 45.57 | 5.3 | 1.44 | 74.29 | 275 |
| 1 | 2 | 59 | 140 | 80 | 22.60 | 5.25 | 3.12 | 1.14 | 3.67 | 5.26 | 7 | 12.65 | 21.84 | 5.5 | 2.96 | 143.75 | 276 |
| 1 | 2 | 49 | 135 | 67 | 26.20 | 5.24 | 7.98 | 1 | 3.24 | 5.39 | 6.58 | 17.29 | 56.83 | 6.1 | 4.14 | 182.96 | 277 |
| 1 | 2 | 51 | 115 | 60 | 22.50 | 5.61 | 2.99 | 1.41 | 3.46 | 4.96 | 6.67 | 9.03 | 35.20 | 5.4 | 1.99 | 123.70 | 277 |
| 1 | 2 | 53 | 160 | 90 | 33.80 | 4.54 | 1.01 | 1.15 | 2.93 | 5.5 | 5.82 | 9.86 | 22.48 | 5.6 | 2.41 | 98.60 | 278 |
| 1 | 2 | 44 | 108 | 70 | 27.60 | 4.27 | 3.26 | 1.03 | 2.51 | 4.4 | 5.98 | 10.79 | 56.74 | 5.2 | 2.11 | 239.78 | 280 |
| 1 | 2 | 40 | 104 | 60 | 20.70 | 3.88 | 0.31 | 1.88 | 1.8 | 5.34 | 5.58 | 9.58 | 28.54 | 5.6 | 2.27 | 104.13 | 281 |
| 1 | 2 | 36 | 115 | 60 | 25.00 | 4.11 | 0.68 | 1.17 | 2.47 | 5.02 | 5.73 | 10.00 | 15.38 | 5.5 | 2.23 | 131.58 | 281 |
| 1 | 2 | 60 | 120 | 65 | 23.40 | 3.33 | 0.5 | 1.22 | 1.46 | 5.27 | 5.88 | 9.61 | 35.39 | 5.8 | 2.25 | 108.59 | 281 |
| 1 | 2 | 57 | 100 | 50 | 26.70 | 5.28 | 0.92 | 1.35 | 3.1 | 5.31 | 4.53 | 9.47 | 20.18 | 5.7 | 2.23 | 104.64 | 281 |
| 1 | 2 | 43 | 130 | 80 | 24.20 | 3.82 | 0.78 | 1.55 | 2.17 | 5.15 | 6.52 | 6.14 | 53.01 | 6.0 | 1.41 | 74.42 | 281 |
| 1 | 2 | 49 | 120 | 70 | 27.90 | 4.44 | 2.7 | 1.3 | 2.27 | 5.23 | 5.04 | 12.58 | 31.54 | 5.6 | 2.92 | 145.43 | 282 |
| 1 | 2 | 52 | 112 | 60 | 25.70 | 5.2 | 1.66 | 1.39 | 3.32 | 5.54 | 4.22 | 9.29 | 28.48 | 5.4 | 2.29 | 91.08 | 282 |
| 1 | 2 | 53 | 124 | 74 | 24.80 | 5.12 | 0.9 | 1.84 | 2.24 | 5.05 | 6.55 | 9.43 | 45.11 | 5.3 | 2.12 | 121.68 | 282 |
| 1 | 2 | 52 | 130 | 74 | 20.70 | 4.11 | 0.64 | 1.45 | 2.18 | 5.46 | 6.04 | 5.00 | 18.31 | 5.8 | 1.21 | 51.02 | 282 |
| 1 | 2 | 53 | 105 | 60 | 24.50 | 5.4 | 0.92 | 2.01 | 2.81 | 4.85 | 6.38 | 7.64 | 74.71 | 5.9 | 1.65 | 113.19 | 282 |
| 1 | 2 | 67 | 130 | 70 | 21.10 | 5.21 | 1.07 | 1.78 | 2.72 | 4.92 | 6.74 | 8.34 | 13.44 | 5.4 | 1.82 | 117.46 | 283 |
| 1 | 2 | 58 | 120 | 75 | 24.40 | 5.59 | 1 | 1.92 | 2.57 | 5.11 | 7.1 | 12.38 | 64.51 | 5.7 | 2.81 | 153.79 | 284 |
| 2 | 1 | 26 | 138 | 97 | 26.40 | 4.74 | 2.11 | 1.07 | 3.08 | 4.62 | 10.29 | 9.25 | 63.15 | 5.4 | 1.90 | 165.18 | 285 |
| 2 | 1 | 27 | 128 | 82 | 29.70 | 4.52 | 7.61 | 0.80 | 2.71 | 5.57 | 8.7 | 4.75 | 59.6 | 5.6 | 1.18 | 45.89 | 285 |
| 2 | 1 | 70 | 132 | 83 | 23.60 | 4.70 | 0.98 | 1.51 | 3.03 | 5.75 | 7.06 | 5.29 | 70.31 | 6.2 | 1.35 | 47.02 | 286 |
| 2 | 1 | 25 | 128 | 85 | 24.70 | 4.56 | 0.90 | 1.20 | 2.93 | 4.85 | 8.33 | 7.38 | 30.36 | 5.4 | 1.59 | 109.33 | 286 |
| 2 | 1 | 51 | 128 | 73 | 28.70 | 4.68 | 1.14 | 1.26 | 3.01 | 5.85 | 10.35 | 6.53 | 56.37 | 5.4 | 1.70 | 55.57 | 286 |
| 2 | 1 | 49 | 135 | 91 | 27.30 | 3.97 | 1.48 | 0.90 | 2.73 | 6.18 | 5.36 | 7.34 | 35 | 5.6 | 2.02 | 54.78 | 286 |
| 2 | 1 | 67 | 138 | 70 | 27.50 | 3.83 | 0.90 | 1.00 | 2.57 | 5.61 | 9.24 | 7.3 | 76.92 | 5.8 | 1.82 | 69.19 | 286 |
| 2 | 1 | 42 | 121 | 86 | 26.40 | 4.60 | 5.45 | 1.06 | 2.60 | 5.21 | 8.96 | 6.27 | 97.1 | 5.6 | 1.45 | 73.33 | 286 |
| 2 | 1 | 65 | 138 | 97 | 24.80 | 4.15 | 1.30 | 0.80 | 2.73 | 5.39 | 8.17 | 8.28 | 80.7 | 5.7 | 1.98 | 87.62 | 286 |
| 2 | 1 | 34 | 155 | 109 | 27.60 | 5.67 | 2.79 | 1.37 | 3.53 | 5.1 | 9.25 | 4.82 | 100.4 | 5.6 | 1.09 | 60.25 | 287 |
| 2 | 1 | 58 | 119 | 71 | 27.70 | 5.19 | 1.43 | 1.07 | 3.63 | 5.3 | 9.13 | 8.25 | 39.44 | 5.5 | 1.94 | 91.67 | 287 |
| 2 | 1 | 53 | 133 | 90 | 27.00 | 5.96 | 0.83 | 1.33 | 4.27 | 5.58 | 8.69 | 7.41 | 69.2 | 5.8 | 1.84 | 71.25 | 287 |
| 2 | 1 | 67 | 110 | 70 | 26.60 | 4.30 | 0.98 | 1.33 | 2.82 | 5.81 | 8.68 | 6.23 | 102.4 | 5.6 | 1.61 | 53.94 | 288 |
| 2 | 1 | 54 | 141 | 80 | 28.10 | 6.35 | 3.48 | 1.21 | 3.99 | 5.7 | 8.84 | 9.01 | 48.64 | 5.6 | 2.28 | 81.91 | 288 |
| 2 | 1 | 58 | 130 | 74 | 30.40 | 4.41 | 2.16 | 1.16 | 2.60 | 5.4 | 8.11 | 3.31 | 53.19 | 5.6 | 0.79 | 34.84 | 288 |
| 2 | 1 | 52 | 125 | 82 | 24.50 | 3.23 | 1.68 | 1.07 | 1.82 | 4.94 | 9.52 | 14.27 | 93.8 | 5.4 | 3.13 | 198.19 | 288 |
| 2 | 1 | 35 | 121 | 81 | 28.30 | 4.05 | 1.47 | 1.51 | 2.61 | 6.84 | 5.93 | 17.56 | 86.9 | 5.0 | 5.34 | 105.15 | 289 |
| 2 | 1 | 59 | 131 | 81 | 25.70 | 4.25 | 0.64 | 1.04 | 2.64 | 5.92 | 7.78 | 4.56 | 56.87 | 5.4 | 1.20 | 37.69 | 289 |
| 2 | 1 | 53 | 136 | 84 | 23.60 | 4.81 | 1.90 | 1.32 | 2.95 | 4.98 | 8.44 | 9.21 | 66.3 | 5.8 | 2.04 | 124.46 | 289 |
| 2 | 1 | 52 | 130 | 78 | 26.90 | 6.24 | 4.29 | 1.12 | 4.10 | 5.73 | 9.13 | 7.21 | 84.6 | 5.7 | 1.84 | 64.66 | 489 |
| 2 | 1 | 54 | 131 | 74 | 27.40 | 6.30 | 3.65 | 1.24 | 4.18 | 6.11 | 7.5 | 10.48 | 37.64 | 5.8 | 2.85 | 80.31 | 289 |
| 2 | 1 | 56 | 121 | 79 | 24.34 | 4.62 | 1.54 | 1.08 | 2.83 | 4.78 | 8.68 | 10.76 | 104.2 | 5.6 | 2.29 | 168.13 | 290 |
| 2 | 1 | 27 | 165 | 80 | 25.40 | 4.53 | 1.69 | 1.14 | 3.31 | 6.4 | 4.32 | 12.33 | 33.76 | 5.3 | 3.51 | 85.03 | 290 |
| 2 | 1 | 35 | 130 | 78 | 24.20 | 3.92 | 3.70 | 1.12 | 2.38 | 5.45 | 7 | 8.24 | 23.13 | 5.8 | 2.00 | 84.51 | 290 |
| 2 | 1 | 35 | 140 | 90 | 25.00 | 5.78 | 4.31 | 1.14 | 3.60 | 5.62 | 8.62 | 5.48 | 72.1 | **5.4** | 1.37 | 51.70 | 290 |
| 2 | 1 | 37 | 140 | 90 | 25.30 | 5.54 | 1.88 | 1.49 | 3.54 | 4.5 | 9.48 | 8.72 | 84.4 | 5.5 | 1.74 | 174.40 | 290 |
| 2 | 1 | 54 | 133 | 88 | 32.50 | 5.17 | 2.38 | 0.92 | 3.60 | 6.03 | 8.05 | 9.79 | 44.03 | 6.2 | 2.62 | 77.39 | 290 |
| 2 | 1 | 29 | 129 | 74 | 32.10 | 4.40 | 1.00 | 0.92 | 3.13 | 5.03 | 8.44 | 7.13 | 90.6 | 5.7 | 1.59 | 93.20 | 291 |
| 2 | 1 | 36 | 132 | 82 | 28.10 | 4.18 | 2.31 | 0.95 | 2.64 | 5.24 | 7.94 | 11.78 | 90.09 | 5.2 | 2.74 | 135.40 | 291 |
| 2 | 1 | 55 | 147 | 94 | 28.60 | 3.89 | 1.85 | 0.80 | 2.54 | 6.76 | 6.66 | 15.02 | 98.2 | 5.8 | 4.51 | 92.15 | 291 |
| 2 | 1 | 53 | 100 | 57 | 27.80 | 2.53 | 0.77 | 0.71 | 1.55 | 5.19 | 10.92 | 12.64 | 87.5 | 5.5 | 2.92 | 149.59 | 291 |
| 2 | 1 | 29 | 129 | 87 | 29.00 | 3.60 | 2.30 | 1.14 | 2.03 | 4.83 | 7.97 | 16.25 | 91.7 | 5.4 | 3.49 | 244.36 | 391 |
| 2 | 1 | 54 | 159 | 87 | 26.20 | 5.67 | 1.83 | 1.51 | 4.41 | 6.5 | 6.88 | 9.18 | 49.69 | 6.4 | 2.65 | 61.20 | 292 |
| 2 | 1 | 52 | 125 | 71 | 30.80 | 4.50 | 2.17 | 0.88 | 2.89 | 6.4 | 10.38 | 11.27 | 67.7 | 5.5 | 3.21 | 77.72 | 292 |
| 2 | 1 | 59 | 158 | 105 | 25.30 | 4.30 | 0.81 | 1.16 | 2.63 | 5.78 | 7.82 | 4.87 | 26.52 | 6.3 | 1.25 | 42.72 | 293 |
| 2 | 1 | 69 | 128 | 77 | 29.50 | 4.94 | 1.27 | 1.48 | 2.93 | 5.03 | 8.89 | 7.91 | 74.22 | 5.4 | 1.77 | 103.40 | 294 |
| 2 | 1 | 51 | 160 | 93 | 24.50 | 4.79 | 11.04 | 0.64 | 2.26 | 5.69 | 9.56 | 11.43 | 87.11 | 5.9 | 2.89 | 104.38 | 294 |
| 2 | 1 | 52 | 152 | 98 | 39.70 | 5.00 | 2.18 | 1.09 | 4.22 | 5.53 | 10.22 | 7.64 | 98.08 | 6.0 | 1.88 | 75.27 | 295 |
| 2 | 1 | 58 | 152 | 81 | 40.00 | 4.27 | 0.97 | 1.11 | 2.77 | 5.21 | 9.28 | 4.41 | 65.8 | 5.5 | 1.02 | 51.58 | 295 |
| 2 | 1 | 64 | 136 | 74 | 24.00 | 3.98 | 0.79 | 1.37 | 1.95 | 5.32 | 7.56 | 5.23 | 36.94 | 5.8 | 1.24 | 57.47 | 295 |
| 2 | 1 | 55 | 146 | 80 | 18.50 | 4.00 | 2.06 | 0.94 | 2.59 | 5.21 | 10.81 | 9.75 | 100 | 5.0 | 2.26 | 114.04 | 295 |
| 2 | 1 | 62 | 140 | 74 | 32.30 | 3.30 | 1.44 | 1.15 | 2.00 | 5.25 | 7.97 | 12.48 | 71.4 | 6.5 | 2.91 | 142.63 | 295 |
| 2 | 1 | 50 | 116 | 74 | 26.60 | 5.70 | 1.98 | 0.93 | 4.21 | 5.19 | 6.56 | 9.34 | 58.07 | 5.4 | 2.15 | 110.53 | 297 |
| 2 | 1 | 53 | 130 | 78 | 28.30 | 5.37 | 1.24 | 1.29 | 3.55 | 5.77 | 7.64 | 4.8 | 57.18 | **5.4** | 1.23 | 42.29 | 297 |
| 2 | 1 | 41 | 147 | 90 | 32.40 | 4.38 | 1.77 | 1.06 | 2.94 | 5.94 | 7.93 | 9.07 | 86.6 | 5.8 | 2.39 | 74.34 | 297 |
| 2 | 1 | 40 | 110 | 71 | 24.40 | 6.15 | 1.62 | 1.44 | 4.23 | 4.82 | 8.13 | 6.77 | 96.84 | 5.5 | 1.45 | 102.58 | 297 |
| 2 | 1 | 56 | 161 | 91 | 28.10 | 3.23 | 1.72 | 0.76 | 2.14 | 5.54 | 8.7 | 6.67 | 46.58 | 5.5 | 1.64 | 65.39 | 298 |
| 2 | 1 | 52 | 119 | 76 | 23.70 | 5.70 | 1.22 | 1.73 | 3.56 | 4.81 | 8.41 | 4.99 | 80.1 | **5.4** | 1.07 | 76.18 | 508 |
| 2 | 1 | 45 | 142 | 95 | 27.10 | 4.58 | 2.36 | 0.92 | 3.13 | 5.39 | 6.62 | 9.21 | 102.9 | 6.7 | 2.21 | 97.46 | 298 |
| 2 | 1 | 60 | 110 | 63 | 25.10 | 4.58 | 1.93 | 0.92 | 2.37 | 6.51 | 5 | 7.55 | 26.24 | 5.6 | 2.18 | 50.17 | 298 |
| 2 | 1 | 52 | 126 | 89 | 24.50 | 5.53 | 2.15 | 1.12 | 3.86 | 6.76 | 7.63 | 9.22 | 97.2 | 5.6 | 2.77 | 56.56 | 299 |
| 2 | 1 | 29 | 130 | 85 | 33.90 | 3.99 | 0.98 | 1.16 | 2.63 | 6.81 | 7.58 | 5.49 | 97.2 | 5.4 | 1.66 | 33.17 | 299 |
| 2 | 1 | 60 | 133 | 73 | 25.10 | 3.58 | 0.55 | 1.25 | 2.19 | 5.33 | 8.26 | 8.96 | 67.45 | 6.1 | 2.12 | 97.92 | 299 |
| 2 | 1 | 54 | 144 | 85 | 28.10 | 4.36 | 0.68 | 1.10 | 3.01 | 5.9 | 8.71 | 7.35 | 79.76 | 6.2 | 1.93 | 61.25 | 299 |
| 2 | 1 | 49 | 128 | 85 | 33.00 | 4.67 | 2.50 | 0.86 | 3.17 | 5.72 | 7.74 | 5.11 | 82.81 | 6.0 | 1.30 | 46.04 | 299 |
| 2 | 1 | 55 | 119 | 76 | 28.60 | 5.10 | 3.69 | 0.97 | 3.42 | 5.65 | 7.85 | 11.29 | 54.96 | 6.1 | 2.84 | 105.02 | 499 |
| 2 | 1 | 29 | 139 | 85 | 28.70 | 5.15 | 3.21 | 1.06 | 3.54 | 5.88 | 8.86 | 12.45 | 81.39 | 6.1 | 3.25 | 104.62 | 300 |
| 2 | 1 | 37 | 135 | 85 | 31.10 | 6.64 | 2.04 | 1.28 | 4.08 | 6.72 | 7.3 | 6.11 | 24.51 | 6.0 | 1.82 | 37.95 | 300 |
| 2 | 1 | 44 | 119 | 74 | 29.40 | 6.70 | 8.72 | 1.18 | 3.41 | 6.66 | 10.76 | 8.77 | 36.73 | 6.3 | 2.60 | 55.51 | 301 |
| 2 | 1 | 38 | 130 | 80 | 23.98 | 6.55 | 4.37 | 1.50 | 4.05 | 5.11 | 8.29 | 13.71 | 41.99 | 5.9 | 3.11 | 170.31 | 301 |
| 2 | 1 | 55 | 131 | 75 | 22.90 | 4.83 | 0.72 | 1.21 | 2.33 | 5.36 | 9.75 | 8.64 | 16.79 | 5.2 | 2.06 | 92.90 | 302 |
| 2 | 1 | 52 | 130 | 76 | 27.20 | 4.45 | 0.74 | 1.28 | 2.73 | 4.58 | 7.81 | 7.18 | 47.15 | 6.2 | 1.46 | 132.96 | 302 |
| 2 | 1 | 38 | 163 | 98 | 34.90 | 4.68 | 3.42 | 0.93 | 3.06 | 5.8 | 9.19 | 6.81 | 85 | **5.4** | 1.76 | 59.22 | 302 |
| 2 | 1 | 65 | 145 | 76 | 25.10 | 4.95 | 1.26 | 1.11 | 4.35 | 6.52 | 10.8 | 6.68 | 39.11 | 6.1 | 1.94 | 44.24 | 302 |
| 2 | 1 | 65 | 150 | 80 | 27.60 | 3.59 | 0.97 | 0.96 | 2.29 | 4.97 | 9.55 | 9.17 | 94.35 | 5.6 | 2.03 | 124.76 | 302 |
| 2 | 1 | 46 | 149 | 80 | 26.20 | 5.35 | 2.72 | 1.34 | 3.36 | 5.76 | 8.71 | 7.24 | 56.07 | 5.8 | 1.85 | 64.07 | 302 |
| 2 | 1 | 54 | 130 | 82 | 26.40 | 4.39 | 3.24 | 1.58 | 2.47 | 5.74 | 7.56 | 7.71 | 84.89 | 6.6 | 1.97 | 68.84 | 303 |
| 2 | 1 | 64 | 121 | 67 | 29.70 | 4.42 | 1.59 | 0.88 | 2.98 | 6.89 | 11.03 | 10.28 | 80.09 | 5.7 | 3.15 | 60.65 | 303 |
| 2 | 1 | 62 | 119 | 77 | 24.20 | 3.86 | 0.91 | 1.17 | 2.36 | 5.88 | 9.23 | 12.6 | 58.01 | 5.8 | 3.29 | 105.88 | 303 |
| 2 | 1 | 38 | 136 | 84 | 30.80 | 4.00 | 1.05 | 1.15 | 2.57 | 5.63 | 9 | 6.59 | 56.66 | 5.4 | 1.65 | 61.88 | 304 |
| 2 | 1 | 55 | 127 | 75 | 23.70 | 5.01 | 0.70 | 1.34 | 3.35 | 6.35 | 7.87 | 4.64 | 51.02 | 6.1 | 1.31 | 32.56 | 304 |
| 2 | 1 | 27 | 123 | 79 | 25.00 | 4.05 | 1.06 | 1.12 | 2.63 | 6.57 | 5.26 | 11.2 | 89.19 | 5.8 | 3.27 | 72.96 | 304 |
| 2 | 1 | 54 | 129 | 76 | 23.30 | 4.57 | 1.99 | 1.09 | 2.94 | 6.43 | 10.96 | 6.19 | 40.3 | 5.4 | 1.77 | 42.25 | 304 |
| 2 | 1 | 51 | 145 | 83 | 28.60 | 5.25 | 2.20 | 0.94 | 3.60 | 6.11 | 6.35 | 13.83 | 73.03 | 5.6 | 3.76 | 105.98 | 305 |
| 2 | 1 | 41 | 134 | 93 | 29.10 | 4.93 | 1.26 | 1.24 | 3.20 | 5.94 | 7.41 | 12.16 | 69.82 | 5.6 | 3.21 | 99.67 | 305 |
| 2 | 1 | 53 | 162 | 98 | 30.10 | 5.49 | 1.52 | 1.11 | 3.80 | 6.14 | 6.07 | 9.54 | 51.68 | 6.1 | 2.60 | 72.27 | 405 |
| 2 | 1 | 38 | 151 | 99 | 25.40 | 5.85 | 2.33 | 1.30 | 3.05 | 6.54 | 4.86 | 10.5 | 51.05 | 5.0 | 3.05 | 69.08 | 605 |
| 2 | 1 | 50 | 124 | 85 | 28.90 | 5.40 | 2.88 | 1.20 | 3.51 | 5.61 | 7.53 | 5.02 | 53.44 | **5.6** | 1.25 | 47.58 | 307 |
| 2 | 1 | 32 | 119 | 69 | 24.80 | 3.51 | 0.74 | 0.98 | 2.27 | 5.58 | 9.29 | 9.56 | 96.13 | 5.8 | 2.37 | 91.92 | 307 |
| 2 | 1 | 50 | 126 | 82 | 29.40 | 5.95 | 5.98 | 1.03 | 3.63 | 5.07 | 9.48 | 5.25 | 104.4 | 5.3 | 1.18 | 66.88 | 308 |
| 2 | 1 | 66 | 149 | 85 | 34.40 | 5.46 | 1.49 | 0.93 | 3.78 | 5.68 | 10.43 | 8.93 | 91.7 | 5.4 | 2.25 | 81.93 | 308 |
| 2 | 1 | 44 | 130 | 86 | 26.70 | 4.13 | 1.24 | 1.17 | 2.68 | 5.09 | 8.35 | 6.02 | 52.48 | 5.7 | 1.36 | 75.72 | 308 |
| 2 | 1 | 44 | 144 | 101 | 30.27 | 6.58 | 9.42 | 1.09 | 3.28 | 5.7 | 7.32 | 9.91 | 47.41 | 5.7 | 2.51 | 90.09 | 308 |
| 2 | 1 | 61 | 128 | 84 | 23.92 | 5.33 | 1.89 | 0.96 | 3.67 | 6.18 | 7.42 | 11.71 | 57.46 | 6.1 | 3.22 | 87.39 | 309 |
| 2 | 1 | 60 | 151 | 92 | 26.91 | 5.18 | 1.37 | 1.11 | 4.37 | 5.62 | 8.94 | 8.47 | 70.28 | 6.2 | 2.12 | 79.91 | 309 |
| 2 | 1 | 60 | 121 | 68 | 21.30 | 6.02 | 1.07 | 1.73 | 3.64 | 6 | 9.42 | 5.83 | 27.39 | 5.2 | 1.55 | 46.64 | 309 |
| 2 | 1 | 61 | 147 | 92 | 26.26 | 3.36 | 1.20 | 1.05 | 1.97 | 5.68 | 8.46 | 12.92 | 105.4 | 6.5 | 3.26 | 118.53 | 309 |
| 2 | 1 | 65 | 118 | 80 | 28.44 | 4.11 | 1.22 | 1.02 | 2.55 | 6.57 | 7.31 | 8.58 | 61.48 | 5.7 | 2.51 | 55.90 | 310 |
| 2 | 1 | 62 | 134 | 82 | 27.79 | 4.00 | 0.98 | 1.48 | 2.18 | 5.91 | 7.79 | 9.49 | 51.97 | 5.8 | 2.49 | 78.76 | 311 |
| 2 | 1 | 40 | 114 | 78 | 26.14 | 4.80 | 2.03 | 1.00 | 3.01 | 6.45 | 6.84 | 14.84 | 67.06 | 5.8 | 4.25 | 100.61 | 311 |
| 2 | 1 | 38 | 144 | 101 | 36.28 | 3.85 | 2.80 | 0.82 | 2.31 | 6.15 | 8.9 | 10.61 | 90.01 | 5.2 | 2.90 | 80.08 | 311 |
| 2 | 1 | 58 | 125 | 68 | 25.50 | 4.14 | 0.87 | 1.14 | 2.27 | 5.25 | 7.3 | 5.28 | 32.53 | 6.1 | 1.23 | 60.34 | 311 |
| 2 | 1 | 49 | 115 | 60 | 40.80 | 4.8 | 1.27 | 1.58 | 2.29 | 6.17 | 9.12 | 15.18 | 72.60 | 7.2 | 4.16 | 113.71 | 311 |
| 2 | 1 | 60 | 130 | 75 | 24.00 | 5.6 | 1.62 | 1.84 | 2.74 | 6.74 | 5.38 | 6.76 | 13.66 | 5.4 | 2.02 | 41.73 | 311 |
| 2 | 1 | 65 | 125 | 75 | 26.80 | 5.07 | 1.85 | 1.46 | 2.71 | 6.01 | 10.98 | 4.40 | 69.96 | 6.8 | 1.18 | 35.06 | 312 |
| 2 | 1 | 66 | 142 | 84 | 26.81 | 3.61 | 2.54 | 1.32 | 1.38 | 5.75 | 8.89 | 11.58 | 62.74 | 5.8 | 2.96 | 102.93 | 312 |
| 2 | 1 | 75 | 118 | 78 | 25.64 | 3.5 | 0.82 | 1.29 | 1.85 | 5.78 | 8.75 | 4.96 | 58.17 | 5.8 | 1.27 | 43.51 | 312 |
| 2 | 1 | 34 | 120 | 65 | 19.80 | 4.52 | 0.35 | 1.7 | 2.39 | 5.66 | 8.42 | 5.66 | 60.39 | 6.1 | 1.42 | 52.41 | 312 |
| 2 | 1 | 72 | 160 | 100 | 30.10 | 4.8 | 1.12 | 1.71 | 2.45 | 6.26 | 8.95 | 8.76 | 65.93 | 6.0 | 2.44 | 63.48 | 313 |
| 2 | 1 | 60 | 120 | 75 | 25.20 | 4.11 | 0.75 | 1.7 | 1.86 | 5.69 | 8.06 | 9.44 | 79.09 | 5.5 | 2.39 | 86.21 | 314 |
| 2 | 1 | 41 | 130 | 75 | 29.40 | 6.56 | 1.89 | 1.22 | 3.74 | 6.01 | 5.7 | 10.43 | 55.24 | 5.6 | 2.79 | 83.11 | 316 |
| 2 | 1 | 63 | 128 | 78 | 24.30 | 4.8 | 0.77 | 1.27 | 3.9 | 5.89 | 9.91 | 11.12 | 48.75 | 6.4 | 2.91 | 93.05 | 317 |
| 2 | 1 | 50 | 120 | 70 | 29.10 | 6.67 | 0.9 | 1.32 | 3.35 | 5.43 | 8.67 | 7.91 | 38.83 | 6.1 | 1.91 | 81.97 | 320 |
| 2 | 1 | 35 | 115 | 65 | 24.30 | 4.27 | 0.86 | 1.59 | 2.36 | 5.71 | 7.89 | 7.50 | 68.16 | 6.0 | 1.90 | 67.87 | 320 |
| 2 | 1 | 38 | 120 | 70 | 26.30 | 4.49 | 3.66 | 1.2 | 2.57 | 5.93 | 7.98 | 16.34 | 25.33 | 6.3 | 4.31 | 134.49 | 320 |
| 2 | 1 | 72 | 120 | 60 | 27.48 | 4.35 | 1.46 | 1.14 | 2.7 | 5.31 | 8.99 | 9.35 | 71.30 | 5.6 | 2.21 | 103.31 | 320 |
| 2 | 1 | 57 | 127 | 70 | 28.40 | 3.95 | 0.69 | 0.92 | 3.05 | 6.78 | 10.4 | 10.42 | 48.21 | 5.5 | 3.14 | 63.54 | 321 |
| 2 | 1 | 38 | 125 | 80 | 32.30 | 5.81 | 2.89 | 1.45 | 3.54 | 5.74 | 7.96 | 8.55 | 58.01 | 5.5 | 2.18 | 76.34 | 321 |
| 2 | 1 | 48 | 126 | 80 | 28.40 | 4.69 | 0.83 | 1.17 | 2.9 | 6.37 | 6.08 | 6.01 | 36.38 | 5.8 | 1.70 | 41.88 | 322 |
| 2 | 1 | 62 | 125 | 75 | 27.87 | 6.11 | 2 | 1.27 | 4.05 | 5.47 | 8.86 | 17.19 | 83.41 | 6.0 | 4.18 | 174.52 | 322 |
| 2 | 1 | 44 | 115 | 74 | 25.20 | 4.13 | 0.74 | 1.24 | 2.29 | 6.93 | 7.34 | 10.67 | 61.44 | 5.7 | 3.29 | 62.22 | 322 |
| 2 | 1 | 63 | 135 | 80 | 26.70 | 5.56 | 1.86 | 1.17 | 3.79 | 6.24 | 7.32 | 7.25 | 61.05 | 5.7 | 2.01 | 52.92 | 322 |
| 2 | 1 | 58 | 123 | 78 | 27.30 | 4.13 | 1.73 | 1.03 | 2.6 | 6.68 | 5.55 | 9.73 | 48.96 | 5.6 | 2.89 | 61.19 | 322 |
| 2 | 1 | 62 | 126 | 68 | 27.50 | 6.09 | 10.83 | 1.06 | 2.73 | 5.67 | 9.23 | 7.74 | 105.70 | 6.5 | 1.95 | 71.34 | 323 |
| 2 | 1 | 27 | 120 | 70 | 27.10 | 5.82 | 5.21 | 1.38 | 3.47 | 4.54 | 10.5 | 12.54 | 89.90 | 5.7 | 2.53 | 241.15 | 324 |
| 2 | 1 | 28 | 124 | 73 | 23.00 | 4.17 | 0.39 | 1.35 | 3.39 | 4.59 | 8.81 | 4.73 | 25.88 | 5.2 | 0.96 | 86.79 | 324 |
| 2 | 1 | 44 | 125 | 88 | 26.60 | 4.21 | 1.65 | 1.31 | 3.69 | 5.18 | 7.16 | 16.66 | 94.44 | 5.6 | 3.84 | 198.33 | 325 |
| 2 | 1 | 55 | 115 | 70 | 25.60 | 2.64 | 0.54 | 0.98 | 1.3 | 5.73 | 7.4 | 7.28 | 66.68 | 5.8 | 1.85 | 65.29 | 325 |
| 2 | 1 | 39 | 131 | 82 | 26.70 | 4.37 | 2.34 | 1.07 | 2.62 | 5.8 | 8.81 | 14.48 | 61.50 | 5.5 | 3.73 | 125.91 | 325 |
| 2 | 1 | 50 | 140 | 95 | 30.50 | 5.39 | 1.65 | 0.97 | 4.02 | 6.74 | 7.28 | 8.78 | 61.93 | 6.1 | 2.63 | 54.20 | 326 |
| 2 | 1 | 64 | 130 | 85 | 26.50 | 4.87 | 0.93 | 1.61 | 3.18 | 4.34 | 9.34 | 11.82 | 45.93 | 5.6 | 2.28 | 281.43 | 328 |
| 2 | 1 | 51 | 120 | 75 | 27.80 | 6.21 | 2.95 | 1.31 | 3.82 | 5.12 | 6.85 | 7.96 | 73.64 | 5.6 | 1.81 | 98.27 | 328 |
| 2 | 1 | 60 | 125 | 70 | 24.50 | 6.23 | 1.46 | 1.18 | 4.39 | 5.93 | 8.68 | 13.25 | 69.36 | 5.9 | 3.49 | 109.05 | 329 |
| 2 | 1 | 52 | 120 | 45 | 27.00 | 5.72 | 1.65 | 1.28 | 3.98 | 5.78 | 7.04 | 10.96 | 58.70 | 5.5 | 2.82 | 96.14 | 329 |
| 2 | 1 | 58 | 168 | 90 | 25.70 | 4.87 | 1.1 | 1.41 | 3.17 | 5.74 | 9.3 | 9.05 | 94.92 | 6.4 | 2.31 | 80.80 | 330 |
| 2 | 1 | 50 | 132 | 91 | 24.20 | 5.27 | 0.67 | 1.42 | 3.39 | 6.21 | 7.49 | 7.19 | 41.33 | 5.6 | 1.98 | 53.06 | 330 |
| 2 | 1 | 51 | 120 | 69 | 26.00 | 4.5 | 0.64 | 1.33 | 2.93 | 5.68 | 8.25 | 5.80 | 39.05 | 6.1 | 1.46 | 53.21 | 331 |
| 2 | 1 | 40 | 120 | 78 | 26.10 | 4.5 | 0.68 | 1.23 | 2.91 | 6.21 | 8.64 | 11.45 | 51.31 | 5.8 | 3.16 | 84.50 | 331 |
| 2 | 1 | 61 | 124 | 80 | 25.40 | 3.45 | 1.23 | 1.14 | 1.07 | 5.86 | 7.31 | 7.48 | 54.01 | **5.6** | 1.95 | 63.39 | 332 |
| 2 | 1 | 37 | 133 | 75 | 29.06 | 4.22 | 0.8 | 1.18 | 2.68 | 5.85 | 7.89 | 7.05 | 55.01 | 6.1 | 1.83 | 60.00 | 334 |
| 2 | 1 | 51 | 124 | 68 | 26.12 | 4.89 | 1.42 | 1.24 | 3.38 | 5.74 | 9.51 | 7.52 | 66.55 | 5.8 | 1.92 | 67.14 | 335 |
| 2 | 1 | 69 | 118 | 68 | 25.10 | 4.36 | 0.91 | 1.26 | 2.6 | 6.28 | 7.58 | 4.35 | 48.24 | 6.4 | 1.21 | 31.29 | 336 |
| 2 | 1 | 63 | 134 | 76 | 29.07 | 6.46 | 1.03 | 1.75 | 4.03 | 5.76 | 7 | 11.00 | 58.71 | 5.6 | 2.82 | 97.35 | 336 |
| 2 | 1 | 61 | 120 | 70 | 32.99 | 4.8 | 1.06 | 1.17 | 3.03 | 6.07 | 8.82 | 10.71 | 51.02 | 5.9 | 2.89 | 83.35 | 338 |
| 2 | 1 | 56 | 132 | 72 | 27.73 | 6.04 | 2.01 | 1.46 | 3.57 | 5.96 | 8.37 | 7.15 | 93.90 | 5.9 | 1.89 | 58.13 | 338 |
| 2 | 1 | 75 | 115 | 79 | 31.55 | 5.1 | 2.09 | 1.36 | 3.59 | 5.4 | 8.73 | 7.76 | 69.53 | 6.0 | 1.86 | 81.68 | 339 |
| 2 | 1 | 31 | 127 | 70 | 31.46 | 5.84 | 1.32 | 1.39 | 3.9 | 6.24 | 6.48 | 13.64 | 60.20 | 5.7 | 3.78 | 99.56 | 340 |
| 2 | 1 | 40 | 128 | 84 | 29.41 | 3.62 | 0.76 | 1.46 | 1.82 | 6.16 | 6.71 | 6.66 | 59.42 | 6.0 | 1.82 | 50.08 | 340 |
| 2 | 1 | 66 | 123 | 70 | 21.38 | 6.44 | 1.9 | 1.28 | 4.74 | 6.13 | 7.89 | 4.73 | 57.14 | 5.4 | 1.29 | 35.97 | 340 |
| 2 | 1 | 63 | 122 | 86 | 27.18 | 4.91 | 1.16 | 1.57 | 3.09 | 6.65 | 5.75 | 6.30 | 60.08 | **5.4** | 1.86 | 40.00 | 340 |
| 2 | 1 | 37 | 134 | 82 | 37.33 | 3.81 | 2.62 | 0.92 | 2.66 | 5.21 | 8.38 | 8.91 | 56.10 | 5.4 | 2.06 | 104.21 | 341 |
| 2 | 1 | 60 | 128 | 85 | 28.98 | 3.81 | 1.16 | 1.11 | 2.61 | 4.75 | 8.65 | 7.81 | 80.82 | 6.1 | 1.65 | 124.96 | 341 |
| 2 | 1 | 69 | 121 | 75 | 31.14 | 4.21 | 1.55 | 0.96 | 2.79 | 6.48 | 6.63 | 11.30 | 55.54 | **5.1** | 3.25 | 75.84 | 342 |
| 2 | 1 | 65 | 118 | 77 | 22.77 | 5.16 | 0.79 | 1.57 | 3.51 | 5.52 | 9.11 | 5.79 | 70.13 | 5.8 | 1.42 | 57.33 | 343 |
| 2 | 1 | 60 | 122 | 72 | 31.23 | 4.93 | 1.53 | 1.04 | 3.63 | 5.1 | 10.23 | 5.74 | 84.70 | 5.9 | 1.30 | 71.75 | 343 |
| 2 | 1 | 63 | 125 | 66 | 22.06 | 5.64 | 1.37 | 1.66 | 3.91 | 5.21 | 7.8 | 5.53 | 41.88 | 5.5 | 1.28 | 64.68 | 344 |
| 2 | 1 | 75 | 111 | 73 | 28.88 | 4.45 | 0.74 | 1.39 | 2.99 | 6.97 | 11.04 | 6.62 | 41.81 | 5.1 | 2.05 | 38.16 | 346 |
| 2 | 1 | 66 | 122 | 72 | 26.85 | 5.3 | 0.92 | 1.14 | 3.68 | 6.26 | 7.26 | 9.46 | 46.34 | 5.5 | 2.63 | 68.55 | 346 |
| 2 | 1 | 31 | 130 | 70 | 27.68 | 4.67 | 1.1 | 1.56 | 3.1 | 5.69 | 7.24 | 9.73 | 50.99 | 6.1 | 2.46 | 88.86 | 347 |
| 2 | 1 | 28 | 128 | 80 | 30.09 | 5.23 | 3.13 | 1.13 | 4.03 | 5.93 | 8.14 | 16.47 | 100.90 | 5.3 | 4.34 | 135.56 | 349 |
| 2 | 1 | 53 | 129 | 65 | 23.41 | 3.96 | 0.31 | 1.67 | 2.28 | 5.89 | 7.57 | 7.22 | 28.37 | 5.2 | 1.89 | 60.42 | 349 |
| 2 | 1 | 79 | 113 | 73 | 29.66 | 5.03 | 0.85 | 1.22 | 3.57 | 5.55 | 8.73 | 6.73 | 53.16 | 5.1 | 1.66 | 65.66 | 549 |
| 2 | 1 | 76 | 126 | 77 | 25.95 | 3.87 | 0.61 | 1.44 | 2.26 | 5.37 | 10.17 | 7.47 | 85.70 | 5.1 | 1.78 | 79.89 | 349 |
| 2 | 1 | 52 | 123 | 65 | 27.06 | 4.6 | 2.77 | 1.59 | 2.71 | 5.46 | 9.07 | 7.44 | 94.00 | 5.9 | 1.81 | 75.92 | 349 |
| 2 | 1 | 72 | 126 | 80 | 30.04 | 4.58 | 2.17 | 1.12 | 2.81 | 6.48 | 8.79 | 10.27 | 91.90 | 6.5 | 2.96 | 68.93 | 350 |
| 2 | 1 | 60 | 125 | 75 | 33.52 | 4.91 | 0.91 | 1.11 | 2.44 | 6.79 | 9.12 | 13.69 | 97.80 | 5.4 | 4.13 | 83.22 | 351 |
| 2 | 1 | 51 | 124 | 74 | 30.86 | 4.4 | 1.7 | 1.37 | 2.88 | 5.85 | 8.96 | 10.88 | 34.56 | 6.1 | 2.83 | 92.60 | 352 |
| 2 | 1 | 82 | 110 | 70 | 29.55 | 4.68 | 1.39 | 1.26 | 3.1 | 5.34 | 8.28 | 8.52 | 68.32 | 5.8 | 2.02 | 92.61 | 354 |
| 2 | 1 | 58 | 128 | 74 | 30.85 | 4.07 | 1.96 | 1.43 | 2.1 | 5.73 | 9.52 | 5.91 | 55.29 | 5.7 | 1.51 | 53.00 | 354 |
| 2 | 1 | 62 | 118 | 80 | 23.66 | 4.77 | 1.13 | 1.36 | 3.42 | 6.57 | 5.98 | 4.79 | 32.62 | 6.1 | 1.40 | 31.21 | 354 |
| 2 | 1 | 60 |  |  | 31.64 | 6.49 | 2.38 | 1.4 | 4.93 | 5.8 | 8.11 | 5.93 | 84.90 | 5.6 | 1.53 | 51.57 | 355 |
| 2 | 1 | 53 | 122 | 72 | 30.86 | 6.86 | 4.15 | 1.22 | 2.67 | 6.51 | 6.41 | 12.95 | 49.78 | 5.5 | 3.75 | 86.05 | 356 |
| 2 | 1 | 47 | 112 | 62 | 29.88 | 5.57 | 0.9 | 1.45 | 3.71 | 6.04 | 7.14 | 7.70 | 50.26 | 5.5 | 2.07 | 60.63 | 360 |
| 2 | 1 | 50 | 111 | 61 | 29.75 | 5.02 | 1.42 | 1.63 | 2.98 | 5.63 | 8.93 | 4.92 | 58.61 | 5.6 | 1.23 | 46.20 | 363 |
| 2 | 1 | 69 | 133 | 83 | 28.15 | 5.75 | 2.38 | 1.24 | 3.43 | 5.51 | 8.67 | 8.54 | 105.50 | 6.5 | 2.09 | 84.98 | 363 |
| 2 | 2 | 26 | 120 | 80 | 19.50 | 4.96 | 0.93 | 1.37 | 3.17 | 5.12 | 7.79 | 8.03 | 56.24 | 5.8 | 1.83 | 99.14 | 385 |
| 2 | 2 | 55 | 141 | 87 | 25.30 | 4.59 | 3.11 | 0.99 | 2.97 | 5.11 | 10.05 | 16.7 | 96.83 | 6.0 | 3.79 | 207.45 | 285 |
| 2 | 2 | 28 | 107 | 71 | 20.10 | 4.95 | 0.66 | 1.23 | 3.34 | 4.55 | 8.63 | 4.55 | 63.53 | 5.4 | 0.92 | 86.67 | 285 |
| 2 | 2 | 27 | 101 | 60 | 27.90 | 5.09 | 0.97 | 1.46 | 2.36 | 6.65 | 5.6 | 8.65 | 33.1 | 5.4 | 2.56 | 54.92 | 285 |
| 2 | 2 | 26 | 111 | 75 | 29.50 | 3.87 | 0.82 | 1.28 | 2.28 | 4.94 | 9.4 | 10.56 | 68.36 | 5.4 | 2.32 | 146.67 | 285 |
| 2 | 2 | 57 | 134 | 82 | 30.00 | 5.18 | 3.93 | 1.13 | 3.24 | 5.28 | 7.44 | 9.48 | 87.37 | 5.9 | 2.22 | 106.52 | 286 |
| 2 | 2 | 62 | 126 | 74 | 25.70 | 5.74 | 2.17 | 1.24 | 3.96 | 5.02 | 8.27 | 9.59 | 83.4 | 6.0 | 2.14 | 126.18 | 286 |
| 2 | 2 | 64 | 124 | 65 | 22.00 | 6.84 | 10.26 | 0.88 | 3.60 | 5.73 | 10.76 | 12.19 | 101.3 | 5.4 | 3.10 | 109.33 | 286 |
| 2 | 2 | 49 | 111 | 66 | 24.80 | 3.52 | 0.89 | 1.42 | 2.98 | 6.1 | 6.04 | 8.62 | 35.33 | 6.1 | 2.34 | 66.31 | 286 |
| 2 | 2 | 51 | 140 | 90 | 30.00 | 4.76 | 1.18 | 1.11 | 3.21 | 5.17 | 7.79 | 10.44 | 61.84 | 5.8 | 2.40 | 125.03 | 287 |
| 2 | 2 | 62 | 159 | 90 | 23.70 | 4.77 | 0.79 | 1.18 | 3.16 | 6.26 | 5.37 | 14.56 | 44.31 | 6.2 | 4.05 | 105.51 | 287 |
| 2 | 2 | 61 | 132 | 84 | 27.20 | 6.21 | 1.62 | 1.31 | 4.18 | 5.45 | 9.12 | 6.64 | 56.23 | 5.8 | 1.61 | 68.10 | 287 |
| 2 | 2 | 25 | 130 | 90 | 41.70 | 6.65 | 2.37 | 1.11 | 3.05 | 4.98 | 7.9 | 5.82 | 93.1 | 5.9 | 1.29 | 78.65 | 287 |
| 2 | 2 | 53 | 119 | 81 | 27.20 | 5.05 | 0.91 | 1.15 | 3.63 | 5.33 | 10.38 | 6.4 | 38.13 | 5.7 | 1.52 | 69.95 | 287 |
| 2 | 2 | 27 | 120 | 70 | 30.50 | 4.17 | 1.38 | 1.38 | 2.48 | 4.98 | 8.54 | 10.67 | 33.46 | 6.0 | 2.36 | 144.19 | 288 |
| 2 | 2 | 57 | 130 | 82 | 28.10 | 5.42 | 1.31 | 1.25 | 3.46 | 4.96 | 10.89 | 7.73 | 54.63 | 5.3 | 1.70 | 105.89 | 288 |
| 2 | 2 | 60 | 129 | 81 | 28.30 | 4.41 | 1.48 | 1.01 | 2.76 | 5.01 | 8.62 | 7.38 | 87 | 5.6 | 1.64 | 97.75 | 288 |
| 2 | 2 | 60 | 100 | 65 | 22.10 | 5.74 | 1.10 | 1.46 | 3.59 | 5.53 | 9.67 | 5.79 | 71.42 | **5.6** | 1.42 | 57.04 | 288 |
| 2 | 2 | 26 | 120 | 80 | 26.60 | 3.24 | 1.62 | 1.39 | 2.72 | 4.47 | 8.48 | 8.09 | 33.72 | 5.9 | 1.61 | 166.80 | 289 |
| 2 | 2 | 60 | 107 | 69 | 28.80 | 6.20 | 1.77 | 1.40 | 3.94 | 6.02 | 10.89 | 13.85 | 69.9 | 5.8 | 3.71 | 109.92 | 289 |
| 2 | 2 | 30 | 114 | 65 | 29.80 | 4.62 | 1.81 | 0.93 | 1.41 | 6.42 | 6.7 | 11.81 | 64.15 | 5.6 | 3.37 | 80.89 | 289 |
| 2 | 2 | 38 | 127 | 85 | 26.90 | 4.45 | 1.02 | 1.17 | 1.85 | 5.22 | 10.24 | 10.14 | 43.83 | 5.6 | 2.35 | 117.91 | 289 |
| 2 | 2 | 56 | 110 | 84 | 26.40 | 6.12 | 1.28 | 1.49 | 3.76 | 6.34 | 5.58 | 16.27 | 32.36 | 5.8 | 4.58 | 114.58 | 289 |
| 2 | 2 | 54 | 120 | 84 | 27.10 | 5.51 | 3.01 | 1.16 | 3.06 | 6.03 | 10.97 | 10.41 | 87.16 | 5.9 | 2.79 | 82.29 | 289 |
| 2 | 2 | 36 | 112 | 72 | 34.30 | 5.12 | 1.64 | 0.94 | 3.61 | 6.11 | 6.1 | 5.05 | 96.28 | 5.8 | 1.37 | 38.70 | 290 |
| 2 | 2 | 23 | 91 | 65 | 18.80 | 4.17 | 1.08 | 1.43 | 2.39 | 4.81 | 9.82 | 8.81 | 101.1 | 5.4 | 1.88 | 134.50 | 290 |
| 2 | 2 | 29 | 113 | 81 | 28.80 | 3.65 | 0.71 | 1.19 | 2.29 | 4.76 | 8.04 | 7.52 | 59.23 | 5.1 | 1.59 | 119.37 | 291 |
| 2 | 2 | 45 | 116 | 75 | 22.40 | 3.83 | 1.85 | 0.88 | 3.47 | 6.03 | 6.12 | 13.14 | 41.96 | 5.3 | 3.52 | 103.87 | 291 |
| 2 | 2 | 41 | 127 | 74 | 26.00 | 4.60 | 2.23 | 0.82 | 2.29 | 5.56 | 7.12 | 11.66 | 83.35 | 5.6 | 2.88 | 113.20 | 291 |
| 2 | 2 | 53 | 151 | 86 | 30.20 | 4.38 | 1.06 | 1.07 | 2.89 | 5.4 | 7.4 | 14.86 | 97.4 | 6.0 | 3.57 | 156.42 | 291 |
| 2 | 2 | 39 | 136 | 78 | 24.30 | 6.06 | 2.70 | 1.15 | 3.96 | 6.53 | 6.25 | 9 | 65.17 | 6.1 | 2.61 | 59.41 | 291 |
| 2 | 2 | 51 | 133 | 81 | 37.20 | 4.35 | 2.77 | 0.98 | 2.67 | 5.74 | 8.75 | 6.22 | 51.11 | 5.5 | 1.59 | 55.54 | 292 |
| 2 | 2 | 35 | 113 | 78 | 23.50 | 4.86 | 1.18 | 1.19 | 3.25 | 6.59 | 8.83 | 15.38 | 104.3 | 5.1 | 4.50 | 99.55 | 293 |
| 2 | 2 | 54 | 107 | 71 | 21.20 | 5.55 | 0.63 | 0.94 | 3.44 | 5.84 | 8.39 | 7.41 | 65.75 | 5.9 | 1.92 | 63.33 | 293 |
| 2 | 2 | 45 | 135 | 89 | 25.80 | 3.94 | 1.19 | 1.02 | 2.47 | 5.71 | 8.63 | 7.19 | 67.76 | 6.1 | 1.82 | 65.07 | 293 |
| 2 | 2 | 25 | 110 | 76 | 27.70 | 3.54 | 1.86 | 1.26 | 2.05 | 4.95 | 8.36 | 9.04 | 70.61 | 5.2 | 1.99 | 124.69 | 294 |
| 2 | 2 | 69 | 128 | 71 | 25.70 | 4.89 | 1.06 | 1.47 | 3.00 | 5.7 | 8.39 | 5.6 | 50.13 | 5.5 | 1.42 | 50.91 | 594 |
| 2 | 2 | 43 | 88 | 56 | 21.10 | 4.05 | 1.00 | 1.42 | 2.25 | 5.27 | 7.36 | 6.21 | 55.03 | 6.0 | 1.45 | 70.17 | 294 |
| 2 | 2 | 54 | 147 | 92 | 28.50 | 4.63 | 1.12 | 1.13 | 3.09 | 6.58 | 5.71 | 16.71 | 62.72 | 4.9 | 4.89 | 108.51 | 294 |
| 2 | 2 | 57 | 114 | 62 | 21.40 | 5.59 | 1.23 | 1.55 | 3.58 | 5.39 | 7.63 | 8.24 | 92.34 | 5.4 | 1.97 | 87.20 | 294 |
| 2 | 2 | 30 | 120 | 73 | 23.80 | 4.45 | 0.57 | 1.11 | 2.93 | 5.64 | 10.93 | 14.36 | 94.5 | 5.6 | 3.60 | 134.21 | 295 |
| 2 | 2 | 53 | 140 | 72 | 26.50 | 5.74 | 1.64 | 1.66 | 3.48 | 6.14 | 8.72 | 7.79 | 65.3 | 6.6 | 2.13 | 59.02 | 295 |
| 2 | 2 | 60 | 115 | 77 | 26.10 | 5.16 | 1.61 | 1.06 | 3.38 | 5.48 | 8.97 | 13.24 | 59.2 | 6.0 | 3.22 | 133.74 | 295 |
| 2 | 2 | 50 | 147 | 80 | 26.60 | 8.28 | 4.72 | 1.47 | 5.10 | 6.72 | 6.29 | 8.57 | 93.77 | 5.6 | 2.56 | 53.23 | 295 |
| 2 | 2 | 65 | 128 | 84 | 28.00 | 4.48 | 1.75 | 1.31 | 2.74 | 5.57 | 7.52 | 11.57 | 64.22 | 6.0 | 2.86 | 111.79 | 295 |
| 2 | 2 | 62 | 135 | 84 | 28.20 | 7.47 | 3.13 | 1.56 | 5.11 | 4.83 | 8.01 | 7.99 | 86.53 | 5.4 | 1.72 | 120.15 | 296 |
| 2 | 2 | 61 | 137 | 82 | 31.20 | 4.40 | 1.43 | 1.38 | 2.33 | 5.85 | 8.08 | 7.42 | 91.28 | 5.7 | 1.93 | 63.15 | 296 |
| 2 | 2 | 51 | 111 | 75 | 25.20 | 6.15 | 2.29 | 1.47 | 4.10 | 5.62 | 8.65 | 13.32 | 87.39 | 5.9 | 3.33 | 125.66 | 296 |
| 2 | 2 | 36 | 121 | 78 | 24.60 | 4.85 | 1.84 | 1.44 | 3.12 | 5.36 | 8.49 | 10.91 | 87.6 | 5.6 | 2.60 | 117.31 | 296 |
| 2 | 2 | 53 | 107 | 69 | 25.60 | 6.32 | 1.30 | 1.43 | 4.40 | 6.73 | 6.25 | 11.05 | 54.47 | 6.1 | 3.31 | 68.42 | 296 |
| 2 | 2 | 55 | 129 | 81 | 37.50 | 5.40 | 1.89 | 1.29 | 3.57 | 6.91 | 5.64 | 12.06 | 30.12 | 5.6 | 3.70 | 70.73 | 296 |
| 2 | 2 | 36 | 110 | 66 | 31.60 | 5.20 | 1.86 | 1.40 | 3.43 | 6.63 | 7.6 | 8.53 | 86.3 | 6.5 | 2.51 | 54.50 | 296 |
| 2 | 2 | 62 | 130 | 81 | 32.20 | 4.10 | 1.82 | 1.17 | 2.61 | 5.21 | 8.48 | 13.59 | 74.2 | 5.4 | 3.15 | 158.95 | 296 |
| 2 | 2 | 40 | 122 | 76 | 28.20 | 3.98 | 0.85 | 1.22 | 2.56 | 6.87 | 6.33 | 11.42 | 56.82 | 6.4 | 3.49 | 67.77 | 296 |
| 2 | 2 | 56 | 136 | 86 | 27.30 | 5.14 | 1.30 | 1.39 | 3.37 | 6.47 | 8.94 | 8.01 | 60.46 | 6.1 | 2.30 | 53.94 | 296 |
| 2 | 2 | 61 | 159 | 95 | 26.50 | 5.16 | 1.04 | 1.18 | 3.56 | 6.56 | 5.68 | 10.56 | 43.95 | 5.4 | 3.08 | 69.02 | 297 |
| 2 | 2 | 51 | 107 | 66 | 25.20 | 4.24 | 1.22 | 1.07 | 2.79 | 5.64 | 7.76 | 4.87 | 39.5 | 5.4 | 1.22 | 45.51 | 297 |
| 2 | 2 | 54 | 134 | 83 | 34.90 | 5.49 | 1.21 | 1.17 | 3.50 | 5.37 | 9.9 | 15.23 | 74.5 | 5.8 | 3.63 | 162.89 | 297 |
| 2 | 2 | 27 | 120 | 70 | 25.80 | 4.88 | 1.76 | 1.17 | 2.49 | 5.02 | 8.19 | 18.61 | 18.96 | 5.7 | 4.15 | 244.87 | 297 |
| 2 | 2 | 61 | 106 | 72 | 20.90 | 5.74 | 1.25 | 1.75 | 3.58 | 4.8 | 8.55 | 15.59 | 90.2 | 6.5 | 3.33 | 239.85 | 298 |
| 2 | 2 | 29 | 130 | 75 | 26.10 | 5.19 | 1.78 | 1.42 | 3.31 | 5.79 | 7.75 | 13.3 | 86.24 | 5.4 | 3.42 | 116.16 | 299 |
| 2 | 2 | 54 | 137 | 81 | 26.30 | 4.17 | 1.35 | 1.10 | 2.68 | 5.69 | 9.78 | 3.81 | 41.85 | 5.5 | 0.96 | 34.79 | 299 |
| 2 | 2 | 40 | 125 | 76 | 25.90 | 5.14 | 1.06 | 1.39 | 3.31 | 6.73 | 5.9 | 5.38 | 48.95 | 6.0 | 1.61 | 33.31 | 300 |
| 2 | 2 | 61 | 137 | 83 | 27.00 | 7.90 | 2.45 | 1.56 | 5.33 | 5.61 | 7.89 | 14.43 | 82.4 | **5.4** | 3.60 | 136.78 | 300 |
| 2 | 2 | 55 | 108 | 70 | 21.10 | 5.77 | 1.91 | 1.40 | 3.87 | 4.52 | 8.69 | 5.77 | 93.47 | 5.8 | 1.16 | 113.14 | 301 |
| 2 | 2 | 50 | 130 | 78 | 27.60 | 4.58 | 1.08 | 1.51 | 2.72 | 6.38 | 9.87 | 6.63 | 75 | 5.8 | 1.88 | 46.04 | 301 |
| 2 | 2 | 53 | 140 | 80 | 23.00 | 5.43 | 1.29 | 1.76 | 3.39 | 6.88 | 5.99 | 9.57 | 56.4 | 5.4 | 2.93 | 56.63 | 302 |
| 2 | 2 | 30 | 111 | 85 | 31.30 | 4.06 | 0.75 | 0.90 | 2.74 | 5.83 | 8.55 | 13.84 | 84.8 | 5.6 | 3.59 | 118.80 | 303 |
| 2 | 2 | 45 | 138 | 88 | 35.20 | 4.34 | 1.70 | 0.86 | 3.00 | 6.41 | 7.78 | 9.55 | 91 | 6.4 | 2.72 | 65.64 | 305 |
| 2 | 2 | 53 | 137 | 85 | 27.30 | 5.90 | 0.84 | 1.92 | 3.65 | 5.66 | 8.9 | 12.01 | 60.59 | 6.1 | 3.02 | 111.20 | 305 |
| 2 | 2 | 56 | 124 | 73 | 28.00 | 6.14 | 1.33 | 1.15 | 4.29 | 5.85 | 10.94 | 12.56 | 103.6 | 5.6 | 3.27 | 106.89 | 305 |
| 2 | 2 | 33 | 102 | 70 | 27.30 | 4.96 | 1.49 | 1.77 | 3.89 | 6.8 | 5.21 | 11.54 | 103 | 5.2 | 3.49 | 69.94 | 306 |
| 2 | 2 | 55 | 118 | 74 | 23.90 | 5.23 | 1.35 | 1.39 | 3.23 | 5.49 | 8.05 | 6.09 | 36.75 | 5.8 | 1.49 | 61.21 | 306 |
| 2 | 2 | 46 | 138 | 94 | 23.80 | 6.52 | 2.49 | 1.39 | 4.41 | 6.71 | 7.72 | 17.06 | 75.22 | 5.5 | 5.09 | 106.29 | 306 |
| 2 | 2 | 64 | 123 | 71 | 26.90 | 6.28 | 1.79 | 1.53 | 4.23 | 6.27 | 7.93 | 14.2 | 100.1 | 5.0 | 3.96 | 102.53 | 306 |
| 2 | 2 | 51 | 125 | 71 | 26.90 | 3.45 | 0.87 | 1.04 | 2.08 | 5.11 | 8.59 | 9.86 | 91.6 | 5.4 | 2.24 | 122.48 | 307 |
| 2 | 2 | 48 | 111 | 68 | 24.60 | 5.71 | 1.05 | 1.57 | 3.58 | 5.57 | 9.71 | 5.85 | 73.5 | 5.6 | 1.45 | 56.52 | 307 |
| 2 | 2 | 38 | 142 | 84 | 25.80 | 5.36 | 1.10 | 1.45 | 3.37 | 6.92 | 8.87 | 7.26 | 93.27 | 5.8 | 2.23 | 42.46 | 307 |
| 2 | 2 | 45 | 110 | 70 | 21.80 | 5.13 | 1.32 | 1.21 | 3.49 | 5.24 | 7.63 | 8.71 | 77.87 | 5.5 | 2.03 | 100.11 | 307 |
| 2 | 2 | 56 | 145 | 83 | 31.70 | 4.87 | 2.88 | 1.44 | 2.93 | 5.8 | 7.38 | 4.11 | 81.9 | 5.9 | 1.06 | 35.74 | 308 |
| 2 | 2 | 60 | 135 | 84 | 24.03 | 5.18 | 1.56 | 1.23 | 3.23 | 5.91 | 7.89 | 10.54 | 47.37 | 5.7 | 2.77 | 87.47 | 308 |
| 2 | 2 | 67 | 148 | 73 | 24.89 | 7.04 | 0.85 | 1.45 | 4.36 | 5.77 | 7.96 | 12.44 | 85.6 | 5.8 | 3.19 | 109.60 | 308 |
| 2 | 2 | 39 | 166 | 97 | 28.20 | 6.02 | 2.85 | 1.39 | 3.58 | 5.66 | 8.6 | 7.67 | 89.6 | **5.4** | 1.93 | 71.02 | 308 |
| 2 | 2 | 63 | 117 | 79 | 23.65 | 6.41 | 1.62 | 1.50 | 4.19 | 5.84 | 8.43 | 7.46 | 40.06 | 5.4 | 1.94 | 63.76 | 309 |
| 2 | 2 | 37 | 112 | 71 | 22.46 | 3.54 | 0.60 | 1.47 | 1.87 | 4.64 | 8.19 | 5.47 | 94.85 | 6.4 | 1.13 | 95.96 | 310 |
| 2 | 2 | 39 | 93 | 67 | 18.37 | 6.48 | 0.59 | 1.62 | 2.47 | 6.03 | 7.25 | 7.61 | 52.78 | 5.7 | 2.04 | 60.16 | 311 |
| 2 | 2 | 54 | 104 | 60 | 25.30 | 5.81 | 0.99 | 1.45 | 3.11 | 5.1 | 10.21 | 7.38 | 13.79 | 6.3 | 1.67 | 92.25 | 311 |
| 2 | 2 | 64 | 140 | 95 | 24.20 | 3.42 | 0.72 | 1.4 | 1.22 | 5.4 | 7.38 | 5.02 | 23.18 | 6.1 | 1.20 | 52.84 | 312 |
| 2 | 2 | 52 | 130 | 75 | 21.76 | 5.2 | 0.5 | 1.21 | 2.2 | 5.55 | 8.68 | 3.41 | 32.08 | 5.6 | 0.84 | 33.27 | 312 |
| 2 | 2 | 70 | 115 | 65 | 22.23 | 5.07 | 3.18 | 1.38 | 2.71 | 6.56 | 6.46 | 4.49 | 23.52 | 6.1 | 1.31 | 29.35 | 312 |
| 2 | 2 | 58 | 110 | 75 | 26.00 | 5.11 | 2.17 | 1.48 | 2.62 | 4.92 | 8.09 | 17.12 | 100.90 | 6.9 | 3.74 | 241.13 | 312 |
| 2 | 2 | 62 | 140 | 74 | 23.30 | 5.29 | 1.51 | 1.98 | 2.35 | 5.39 | 7.78 | 10.28 | 51.78 | 6.4 | 2.46 | 108.78 | 313 |
| 2 | 2 | 48 | 108 | 70 | 27.30 | 7.75 | 3.65 | 1.92 | 2.03 | 5.81 | 7.29 | 6.73 | 41.94 | 6.1 | 1.74 | 58.27 | 313 |
| 2 | 2 | 51 | 114 | 70 | 24.20 | 4.76 | 0.99 | 1.46 | 2.11 | 6.07 | 6.68 | 4.87 | 40.77 | 6.6 | 1.31 | 37.90 | 313 |
| 2 | 2 | 65 | 128 | 77 | 21.50 | 5.69 | 1.07 | 0.94 | 2.68 | 5.64 | 10.07 | 8.00 | 48.01 | 5.7 | 2.01 | 74.77 | 314 |
| 2 | 2 | 58 | 122 | 65 | 25.00 | 6.48 | 0.63 | 1.67 | 2.17 | 5.89 | 7.94 | 4.22 | 41.93 | 5.9 | 1.10 | 35.31 | 315 |
| 2 | 2 | 62 | 144 | 77 | 30.50 | 4.02 | 1.17 | 1.21 | 1.89 | 5.78 | 8.24 | 6.68 | 91.56 | 5.7 | 1.72 | 58.60 | 315 |
| 2 | 2 | 41 | 120 | 70 | 26.87 | 3.58 | 1.27 | 1.25 | 1.61 | 6.84 | 6.66 | 4.15 | 60.01 | 6.1 | 1.26 | 24.85 | 315 |
| 2 | 2 | 46 | 115 | 65 | 25.00 | 4.1 | 2.5 | 1.11 | 2.2 | 6.94 | 10.13 | 13.89 | 72.80 | 5.6 | 4.28 | 80.76 | 316 |
| 2 | 2 | 64 | 131 | 71 | 23.12 | 3.68 | 1.03 | 1.04 | 1.83 | 6.01 | 5.51 | 5.04 | 62.19 | 5.7 | 1.35 | 40.16 | 316 |
| 2 | 2 | 34 | 120 | 80 | 21.60 | 4.22 | 1 | 1.56 | 2.77 | 5.72 | 7.86 | 8.92 | 55.02 | 6.1 | 2.27 | 80.36 | 316 |
| 2 | 2 | 61 | 130 | 86 | 23.70 | 5.77 | 5.47 | 1.36 | 3.16 | 5.96 | 8.68 | 7.84 | 56.90 | 6.2 | 2.08 | 63.74 | 316 |
| 2 | 2 | 56 | 140 | 75 | 24.40 | 5.26 | 0.61 | 1.22 | 2.61 | 6.93 | 8.07 | 3.34 | 35.49 | 5.7 | 1.03 | 19.48 | 316 |
| 2 | 2 | 44 | 109 | 75 | 28.10 | 5.69 | 1.64 | 1.36 | 3.58 | 5.76 | 8.2 | 13.37 | 82.10 | **5.4** | 3.42 | 118.32 | 317 |
| 2 | 2 | 58 | 114 | 78 | 31.30 | 3.95 | 1.8 | 1.04 | 2.84 | 6.85 | 10.71 | 9.91 | 89.80 | 6.0 | 3.02 | 59.16 | 317 |
| 2 | 2 | 57 | 125 | 65 | 30.40 | 5.31 | 2.18 | 1.37 | 3.74 | 5.17 | 8.02 | 11.46 | 43.60 | 5.7 | 2.63 | 137.25 | 317 |
| 2 | 2 | 38 | 110 | 60 | 29.70 | 4.2 | 1.3 | 1.26 | 3 | 5.69 | 8.67 | 7.32 | 99.90 | 5.8 | 1.85 | 66.85 | 319 |
| 2 | 2 | 69 | 114 | 65 | 28.30 | 5.59 | 1.84 | 1.27 | 3.48 | 5.49 | 9.97 | 4.57 | 49.68 | 6.0 | 1.12 | 45.93 | 319 |
| 2 | 2 | 33 | 118 | 70 | 21.50 | 4.68 | 0.6 | 1.28 | 2.59 | 6.05 | 7.74 | 7.07 | 51.46 | 6.1 | 1.90 | 55.45 | 320 |
| 2 | 2 | 68 | 130 | 60 | 26.30 | 4.61 | 0.99 | 1.28 | 3.85 | 6.87 | 7.44 | 10.91 | 73.80 | 5.6 | 3.33 | 64.75 | 320 |
| 2 | 2 | 43 | 105 | 60 | 31.20 | 3.9 | 0.68 | 1.23 | 2.39 | 5.6 | 7.56 | 7.77 | 37.34 | 6.9 | 1.93 | 74.00 | 320 |
| 2 | 2 | 65 | 170 | 80 | 28.90 | 5.07 | 3.48 | 1.07 | 5.5 | 6.88 | 11.04 | 12.40 | 69.24 | 6.7 | 3.79 | 73.37 | 320 |
| 2 | 2 | 36 | 100 | 60 | 25.10 | 3.8 | 0.36 | 0.59 | 1.88 | 4.7 | 8.9 | 6.57 | 23.13 | 5.8 | 1.37 | 109.50 | 320 |
| 2 | 2 | 66 | 120 | 80 | 21.50 | 4.55 | 0.66 | 1.24 | 2.13 | 4.76 | 7.43 | 3.64 | 38.80 | 6.1 | 0.77 | 57.78 | 320 |
| 2 | 2 | 52 | 160 | 100 | 32.90 | 4.76 | 1.35 | 1.48 | 2.84 | 5.12 | 7.44 | 8.01 | 44.11 | 6.2 | 1.82 | 98.89 | 321 |
| 2 | 2 | 56 | 140 | 85 | 28.00 | 4.26 | 0.45 | 1.32 | 2.57 | 4.86 | 8.72 | 9.77 | 71.08 | 6.2 | 2.11 | 143.68 | 321 |
| 2 | 2 | 56 | 155 | 90 | 23.90 | 4.53 | 0.98 | 1.64 | 2.49 | 5.64 | 8.79 | 7.45 | 36.58 | 5.8 | 1.87 | 69.63 | 322 |
| 2 | 2 | 50 | 138 | 80 | 30.00 | 6.49 | 2.83 | 1.14 | 3.1 | 5.29 | 8.08 | 7.87 | 88.06 | 5.9 | 1.85 | 87.93 | 322 |
| 2 | 2 | 54 | 125 | 66 | 24.20 | 4.11 | 0.71 | 1.26 | 2.49 | 5.7 | 7.8 | 8.32 | 37.56 | 5.2 | 2.11 | 75.64 | 322 |
| 2 | 2 | 60 | 130 | 73 | 29.40 | 4.86 | 1.49 | 1.25 | 3.27 | 6.61 | 6.59 | 7.90 | 96.60 | 6.6 | 2.32 | 50.80 | 323 |
| 2 | 2 | 63 | 140 | 74 | 33.10 | 7.38 | 3.85 | 1.36 | 4.51 | 6.27 | 5.57 | 5.42 | 57.85 | 5.8 | 1.51 | 39.13 | 324 |
| 2 | 2 | 60 | 120 | 75 | 27.30 | 7.37 | 2.13 | 1.32 | 4.69 | 5.59 | 9.41 | 3.73 | 72.10 | 6.1 | 0.93 | 35.69 | 324 |
| 2 | 2 | 50 | 130 | 70 | 23.30 | 5.48 | 1.93 | 1.5 | 3.03 | 5.84 | 7.85 | 10.35 | 44.20 | 6.2 | 2.69 | 88.46 | 324 |
| 2 | 2 | 62 | 130 | 70 | 24.40 | 4.95 | 1.11 | 1.11 | 2.42 | 5.75 | 8.11 | 5.57 | 37.35 | 6.9 | 1.42 | 49.51 | 325 |
| 2 | 2 | 63 | 123 | 72 | 20.20 | 5.7 | 1.55 | 1.38 | 3.84 | 5.27 | 8.86 | 8.31 | 74.31 | 5.4 | 1.95 | 93.90 | 325 |
| 2 | 2 | 53 | 120 | 70 | 22.00 | 4.37 | 1 | 1.35 | 2.73 | 6.79 | 5.97 | 10.45 | 64.92 | 6.1 | 3.15 | 63.53 | 325 |
| 2 | 2 | 64 | 145 | 90 | 23.90 | 6.78 | 0.94 | 1.11 | 4.28 | 6.89 | 7.33 | 10.58 | 40.77 | 6.1 | 3.24 | 62.42 | 325 |
| 2 | 2 | 41 | 123 | 63 | 19.70 | 4.33 | 0.39 | 1.16 | 2.07 | 6.63 | 5.39 | 7.02 | 32.02 | 5.6 | 2.07 | 44.86 | 325 |
| 2 | 2 | 55 | 117 | 62 | 28.70 | 4.89 | 1.43 | 1.25 | 3.33 | 5.38 | 7.38 | 8.45 | 61.75 | 5.8 | 2.02 | 89.89 | 325 |
| 2 | 2 | 60 | 110 | 68 | 24.20 | 5.55 | 3.94 | 1.13 | 3.44 | 6.01 | 7.34 | 8.40 | 45.25 | 6.1 | 2.24 | 66.93 | 325 |
| 2 | 2 | 63 | 104 | 65 | 21.70 | 5.09 | 1.7 | 1.32 | 3.48 | 4.74 | 8.23 | 9.11 | 69.04 | 6.7 | 1.92 | 146.94 | 326 |
| 2 | 2 | 48 | 130 | 80 | 22.30 | 4.58 | 1.6 | 1.22 | 2.91 | 4.8 | 8.16 | 9.50 | 94.40 | 5.7 | 2.03 | 146.15 | 328 |
| 2 | 2 | 50 | 115 | 58 | 26.40 | 6.7 | 1.42 | 1.6 | 4.27 | 5.27 | 8.67 | 6.45 | 47.27 | 6.2 | 1.51 | 72.88 | 328 |
| 2 | 2 | 61 | 140 | 80 | 26.30 | 4.79 | 1.19 | 1.21 | 3.25 | 5.22 | 7.31 | 8.66 | 84.31 | 5.7 | 2.01 | 100.70 | 328 |
| 2 | 2 | 34 | 135 | 74 | 30.00 | 4.49 | 1.98 | 1.13 | 3.08 | 5.77 | 7.85 | 12.59 | 68.20 | 6.5 | 3.23 | 110.93 | 329 |
| 2 | 2 | 65 | 107 | 63 | 22.20 | 4.51 | 1.54 | 1.04 | 2.62 | 4.72 | 8.68 | 8.19 | 42.28 | 5.6 | 1.72 | 134.26 | 329 |
| 2 | 2 | 60 | 152 | 80 | 22.30 | 5.49 | 0.98 | 1.52 | 3.51 | 6.34 | 8.69 | 6.36 | 73.79 | 5.6 | 1.79 | 44.79 | 329 |
| 2 | 2 | 50 | 116 | 70 | 32.00 | 6.11 | 0.6 | 1.15 | 3.37 | 5.66 | 7.77 | 5.07 | 75.07 | 6.1 | 1.28 | 46.94 | 329 |
| 2 | 2 | 46 | 125 | 78 | 23.50 | 4.01 | 0.86 | 1.36 | 2.26 | 5.97 | 9.53 | 7.84 | 50.80 | 6.3 | 2.08 | 63.48 | 331 |
| 2 | 2 | 59 | 108 | 69 | 22.30 | 5.85 | 0.74 | 1.63 | 3.55 | 5.66 | 7.74 | 7.25 | 79.00 | 5.5 | 1.82 | 67.13 | 331 |
| 2 | 2 | 51 | 127 | 84 | 28.90 | 3.94 | 7.17 | 0.84 | 1.81 | 5.48 | 7.35 | 5.62 | 91.90 | 6.5 | 1.37 | 56.77 | 331 |
| 2 | 2 | 60 | 132 | 84 | 24.80 | 5 | 1.73 | 1.53 | 3 | 5.51 | 6.98 | 8.00 | 49.71 | 5.7 | 1.96 | 79.60 | 332 |
| 2 | 2 | 50 | 125 | 75 | 24.03 | 6.02 | 1.84 | 1.56 | 3.93 | 5.47 | 9.42 | 6.74 | 98.10 | 5.9 | 1.64 | 68.43 | 332 |
| 2 | 2 | 50 | 132 | 80 | 22.38 | 3.36 | 1.13 | 1.21 | 1.8 | 5.73 | 8.28 | 9.91 | 65.87 | 5.9 | 2.52 | 88.88 | 332 |
| 2 | 2 | 62 | 126 | 75 | 20.83 | 5.76 | 1.02 | 1.55 | 3.19 | 5.78 | 8.9 | 8.88 | 45.81 | 5.9 | 2.28 | 77.89 | 333 |
| 2 | 2 | 50 | 121 | 71 | 26.56 | 3.89 | 1.26 | 1.18 | 2.04 | 5.61 | 7.74 | 11.81 | 73.10 | 5.6 | 2.94 | 111.94 | 333 |
| 2 | 2 | 78 | 127 | 77 | 33.76 | 4.98 | 1.92 | 1.57 | 2.9 | 5.72 | 7.71 | 15.28 | 70.00 | 5.8 | 3.88 | 137.66 | 334 |
| 2 | 2 | 51 | 122 | 72 | 31.22 | 3.81 | 1.1 | 1.3 | 2.24 | 5.04 | 9.09 | 10.75 | 44.71 | 5.8 | 2.41 | 139.61 | 336 |
| 2 | 2 | 63 | 132 | 68 | 23.50 | 6 | 1.88 | 1.51 | 4.34 | 5.3 | 7.81 | 14.88 | 79.20 | 5.6 | 3.51 | 165.33 | 336 |
| 2 | 2 | 45 | 123 | 81 | 24.39 | 4.23 | 0.91 | 1.23 | 2.63 | 5.81 | 7.96 | 6.91 | 68.30 | 6.1 | 1.78 | 59.83 | 337 |
| 2 | 2 | 44 | 133 | 84 | 26.73 | 5.69 | 1.06 | 1.36 | 3.96 | 6.27 | 7.12 | 10.48 | 57.36 | 6.1 | 2.92 | 75.67 | 337 |
| 2 | 2 | 62 | 124 | 89 | 26.45 | 5.92 | 1.78 | 1.21 | 4.44 | 6.04 | 9.32 | 11.83 | 76.00 | 6.0 | 3.18 | 93.15 | 338 |
| 2 | 2 | 75 | 108 | 72 | 21.23 | 7.85 | 2.51 | 1.77 | 5.18 | 5.73 | 8.75 | 6.18 | 53.49 | **5.6** | 1.57 | 55.43 | 338 |
| 2 | 2 | 63 | 132 | 72 | 23.14 | 6.9 | 0.85 | 1.15 | 4.72 | 6.02 | 6.09 | 6.12 | 34.66 | 6.1 | 1.64 | 48.57 | 339 |
| 2 | 2 | 59 | 133 | 69 | 23.50 | 5.91 | 0.97 | 1.8 | 3.84 | 5.29 | 8.17 | 6.41 | 102.70 | 5.6 | 1.51 | 71.62 | 340 |
| 2 | 2 | 63 | 120 | 70 | 36.92 | 5.54 | 1.9 | 1.32 | 2.31 | 5.37 | 7.76 | 10.30 | 104.90 | 5.2 | 2.46 | 110.16 | 340 |
| 2 | 2 | 61 | 109 | 71 | 28.88 | 5.68 | 1.19 | 1.94 | 2.8 | 5.22 | 8.05 | 5.04 | 32.29 | 5.7 | 1.17 | 58.60 | 340 |
| 2 | 2 | 45 | 121 | 71 | 24.84 | 3.06 | 1.86 | 0.93 | 1.44 | 6.63 | 6.43 | 12.57 | 38.98 | 6.1 | 3.70 | 80.32 | 341 |
| 2 | 2 | 60 | 122 | 80 | 24.34 | 6.14 | 2.58 | 1.34 | 3.7 | 5.82 | 7.87 | 12.17 | 45.41 | **5.4** | 3.15 | 104.91 | 341 |
| 2 | 2 | 67 | 108 | 70 | 28.80 | 4.04 | 0.52 | 1.42 | 2.21 | 5.09 | 7.96 | 7.51 | 77.33 | 5.2 | 1.70 | 94.47 | 341 |
| 2 | 2 | 58 | 128 | 77 | 27.34 | 4.79 | 0.83 | 1.5 | 3.1 | 5.13 | 10.83 | 6.10 | 27.06 | **5.6** | 1.39 | 74.85 | 341 |
| 2 | 2 | 63 | 125 | 69 | 26.12 | 5.1 | 1.68 | 1.51 | 3.2 | 5.41 | 10.38 | 6.32 | 92.95 | 5.4 | 1.52 | 66.18 | 342 |
| 2 | 2 | 65 | 132 | 76 | 30.49 | 5.36 | 1.83 | 1.35 | 3.13 | 5.39 | 7.49 | 14.60 | 87.00 | 6.6 | 3.50 | 154.50 | 343 |
| 2 | 2 | 49 | 127 | 78 | 22.14 | 3.73 | 0.49 | 1.5 | 1.94 | 5.42 | 8.1 | 3.68 | 56.85 | 5.5 | 0.89 | 38.33 | 343 |
| 2 | 2 | 66 | 130 | 79 | 22.64 | 4.74 | 1.06 | 1.23 | 2.51 | 4.93 | 9.21 | 7.07 | 28.73 | **5.6** | 1.55 | 98.88 | 344 |
| 2 | 2 | 63 | 120 | 70 | 24.84 | 4.44 | 0.54 | 1.89 | 2.09 | 5.68 | 9.07 | 7.06 | 83.66 | 5.6 | 1.78 | 64.77 | 344 |
| 2 | 2 | 50 | 128 | 64 | 23.38 | 3.4 | 0.5 | 1.45 | 1.83 | 6.93 | 5.73 | 9.37 | 51.30 | 6.1 | 2.89 | 54.64 | 344 |
| 2 | 2 | 65 | 142 | 88 | 25.33 | 5.82 | 4.28 | 1.46 | 3.56 | 5.78 | 8.8 | 9.51 | 85.40 | 5.7 | 2.44 | 83.42 | 345 |
| 2 | 2 | 74 | 120 | 70 | 26.88 | 3.74 | 0.57 | 1.73 | 1.75 | 6.06 | 10.3 | 11.55 | 98.40 | 5.6 | 3.11 | 90.23 | 345 |
| 2 | 2 | 30 | 132 | 92 | 45.52 | 6.4 | 2.31 | 1.38 | 4.95 | 5.32 | 7.42 | 13.25 | 75.56 | 5.1 | 3.13 | 145.60 | 347 |
| 2 | 2 | 60 | 120 | 79 | 22.96 | 4.19 | 0.55 | 1.18 | 1.96 | 6.1 | 8.76 | 5.95 | 62.27 | 5.1 | 1.61 | 45.77 | 347 |
| 2 | 2 | 52 | 142 | 88 | 25.39 | 4.61 | 0.61 | 1.05 | 2.55 | 5.78 | 9.16 | 9.54 | 65.76 | 5.5 | 2.45 | 83.68 | 349 |
| 2 | 2 | 78 | 125 | 74 | 28.04 | 3.74 | 0.93 | 1.1 | 1.67 | 6.41 | 6.95 | 13.57 | 90.80 | 5.4 | 3.87 | 93.26 | 349 |
| 2 | 2 | 71 | 119 | 78 | 22.89 | 5.7 | 1.07 | 1.7 | 3.76 | 5.24 | 9.12 | 5.57 | 63.33 | 5.5 | 1.30 | 64.02 | 450 |
| 2 | 2 | 66 | 130 | 70 | 26.35 | 5.48 | 1.62 | 1.64 | 3.48 | 5.6 | 9.12 | 12.63 | 72.84 | 5.5 | 3.14 | 120.29 | 350 |
| 2 | 2 | 65 | 120 | 70 | 26.22 | 6.3 | 1.12 | 2 | 4.26 | 5.28 | 10.29 | 7.82 | 68.47 | 6.0 | 1.84 | 87.87 | 350 |
| 2 | 2 | 74 | 134 | 74 | 23.24 | 4.88 | 2.7 | 1.33 | 2.98 | 6.96 | 10.31 | 4.97 | 38.55 | 5.7 | 1.54 | 28.73 | 350 |
| 2 | 2 | 64 | 125 | 90 | 26.48 | 3.83 | 0.69 | 1.01 | 1.77 | 6.24 | 9.88 | 17.66 | 90.00 | 6.4 | 4.90 | 128.91 | 350 |
| 2 | 2 | 58 | 120 | 62 | 27.01 | 6.56 | 1.37 | 0.98 | 4.07 | 5.51 | 7.37 | 10.61 | 47.57 | 5.7 | 2.60 | 105.57 | 350 |
| 2 | 2 | 60 | 124 | 70 | 29.11 | 4.24 | 0.87 | 1.65 | 2.29 | 5.9 | 9.53 | 12.03 | 103.80 | 5.6 | 3.15 | 100.25 | 350 |
| 2 | 2 | 70 | 132 | 75 | 27.41 | 6.62 | 2.96 | 1.34 | 4.6 | 5.1 | 9.62 | 13.99 | 65.20 | 5.9 | 3.17 | 174.88 | 350 |
| 2 | 2 | 59 | 131 | 80 | 22.37 | 4.74 | 1.24 | 1.12 | 2.99 | 6.6 | 7.6 | 6.57 | 57.47 | 5.8 | 1.93 | 42.39 | 351 |
| 2 | 2 | 61 | 122 | 72 | 26.58 | 3.75 | 0.98 | 1.04 | 2.47 | 6.21 | 5.49 | 8.53 | 63.42 | 5.4 | 2.35 | 62.95 | 352 |
| 2 | 2 | 60 | 126 | 72 | 25.78 | 5.49 | 4.15 | 1.29 | 2.92 | 6.63 | 8.82 | 9.12 | 96.65 | 6.0 | 2.69 | 58.27 | 353 |
| 2 | 2 | 60 | 127 | 77 | 24.20 | 6.05 | 1.35 | 1.99 | 3.19 | 5.66 | 8.63 | 5.01 | 55.74 | 5.4 | 1.26 | 46.39 | 354 |
| 2 | 2 | 54 | 120 | 70 | 32.39 | 5.68 | 0.74 | 1.6 | 3.79 | 5.29 | 8.42 | 9.62 | 85.78 | 5.5 | 2.26 | 107.49 | 354 |
| 2 | 2 | 55 | 126 | 70 | 26.12 | 5.83 | 1.67 | 1.36 | 4.52 | 6.05 | 7.79 | 8.96 | 100.00 | 5.0 | 2.41 | 70.27 | 354 |
| 2 | 2 | 60 | 120 | 82 | 28.48 | 5.67 | 1.88 | 1.51 | 3.89 | 6.71 | 6.62 | 8.08 | 54.47 | **5.6** | 2.41 | 50.34 | 354 |
| 2 | 2 | 55 | 108 | 68 | 35.67 | 5.36 | 6.09 | 1.24 | 2.85 | 5.53 | 8.09 | 6.10 | 55.04 | 6.0 | 1.50 | 60.10 | 355 |
| 2 | 2 | 50 | 121 | 75 | 25.63 | 4.63 | 1.11 | 1.02 | 2.72 | 5.32 | 9.44 | 9.67 | 70.21 | **5.2** | 2.29 | 106.26 | 356 |
| 2 | 2 | 73 | 121 | 85 | 27.11 | 6.1 | 1.32 | 1.6 | 4.19 | 5.99 | 8.04 | 6.97 | 64.01 | 5.4 | 1.86 | 55.98 | 360 |
| 2 | 2 | 53 | 131 | 75 | 30.44 | 4.84 | 1.66 | 1.19 | 3.26 | 5.7 | 7.1 | 14.69 | 63.06 | 5.5 | 3.72 | 133.55 | 360 |
| 2 | 2 | 56 | 125 | 71 | 29.21 | 4.12 | 1.08 | 1.16 | 2.57 | 6.06 | 8.51 | 10.26 | 45.98 | 5.9 | 2.76 | 80.16 | 312 |
| 3 | 1 | 38 | 135 | 90 | 28.50 | 5.88 | 3.40 | 1.24 | 3.96 | 8.95 | 13.48 | 10.79 | 57.17 | 8.6 | 4.29 | 39.60 | 364 |
| 3 | 1 | 61 | 127 | 72 | 25.00 | 5.38 | 1.69 | 0.88 | 3.87 | 7.56 | 10.47 | 8.02 | 37.31 | 8.6 | 2.69 | 39.51 | 364 |
| 3 | 1 | 50 | 135 | 87 | 25.40 | 6.14 | 3.31 | 1.30 | 3.99 | 8.23 | 12.9 | 11.97 | 43.67 | 7.9 | 4.38 | 50.61 | 364 |
| 3 | 1 | 64 | 140 | 90 | 23.40 | 2.79 | 1.77 | 0.78 | 1.79 | 6.89 | 15.08 | 10.26 | 63.45 | 7.9 | 3.14 | 60.53 | 365 |
| 3 | 1 | 54 | 127 | 75 | 29.60 | 6.82 | 3.92 | 0.80 | 2.28 | 7.71 | 15.24 | 10.22 | 38.13 | 7.9 | 3.50 | 48.55 | 365 |
| 3 | 1 | 58 | 132 | 91 | 27.10 | 5.50 | 3.83 | 1.04 | 3.44 | 7.86 | 13.24 | 5.99 | 72.9 | 7.9 | 2.09 | 27.48 | 365 |
| 3 | 1 | 59 | 122 | 83 | 24.60 | 3.49 | 2.05 | 0.97 | 2.02 | 5.03 | 12.59 | 10.1 | 50.9 | 7.9 | 2.26 | 132.03 | 365 |
| 3 | 1 | 69 | 120 | 74 | 33.40 | 4.23 | 0.84 | 1.44 | 2.40 | 6.02 | 12.2 | 7.89 | 68.96 | 9.6 | 2.11 | 62.62 | 365 |
| 3 | 1 | 55 | 140 | 90 | 29.40 | 5.94 | 1.82 | 1.09 | 4.13 | 6 | 12.71 | 10.39 | 65.93 | 9.6 | 2.77 | 83.12 | 365 |
| 3 | 1 | 55 | 145 | 95 | 34.80 | 4.45 | 2.72 | 0.80 | 2.76 | 6.79 | 11.29 | 8.91 | 55.2 | 9.6 | 2.69 | 54.16 | 365 |
| 3 | 1 | 38 | 115 | 63 | 26.30 | 5.94 | 2.84 | 1.32 | 3.87 | 6.81 | 13.72 | 14.67 | 66.15 | 9.6 | 4.44 | 88.64 | 365 |
| 3 | 1 | 37 | 130 | 88 | 25.90 | 4.72 | 1.86 | 0.96 | 3.25 | 6.39 | 11.08 | 10.96 | 71.24 | 9.6 | 3.11 | 75.85 | 365 |
| 3 | 1 | 61 | 141 | 66 | 22.90 | 4.51 | 1.05 | 1.43 | 2.89 | 6.78 | 11.1 | 5.24 | 40.17 | 7.8 | 1.58 | 31.95 | 368 |
| 3 | 1 | 52 | 144 | 95 | 24.70 | 5.88 | 2.46 | 1.31 | 3.83 | 8.23 | 16.41 | 8.01 | 29.27 | 7.3 | 2.93 | 33.87 | 368 |
| 3 | 1 | 40 | 159 | 100 | 29.30 | 6.12 | 2.04 | 1.08 | 4.44 | 11.7 | 13.72 | 9.69 | 55.43 | 6.4 | 5.05 | 23.58 | 368 |
| 3 | 1 | 40 | 148 | 32 | 34.22 | 4.38 | 1.69 | 0.98 | 3.00 | 5.41 | 11.2 | 5.2 | 63.08 | 5.9 | 1.25 | 54.45 | 368 |
| 3 | 1 | 61 | 145 | 73 | 28.00 | 3.21 | 2.16 | 0.71 | 2.37 | 9.07 | 16.76 | 8.44 | 33.34 | 6.3 | 3.40 | 30.31 | 369 |
| 3 | 1 | 48 | 133 | 85 | 29.20 | 5.13 | 2.85 | 1.00 | 3.45 | 8.14 | 14.65 | 12.12 | 50.1 | 8.1 | 4.38 | 52.24 | 369 |
| 3 | 1 | 40 | 156 | 107 | 34.20 | 6.62 | 1.42 | 0.88 | 2.43 | 10.8 | 14.47 | 6.6 | 40.57 | 6.2 | 3.17 | 18.03 | 370 |
| 3 | 1 | 48 | 131 | 85 | 26.70 | 4.59 | 1.62 | 1.18 | 2.96 | 6.18 | 16.34 | 6.14 | 47.45 | 13.2 | 1.69 | 45.82 | 370 |
| 3 | 1 | 54 | 127 | 92 | 35.30 | 6.73 | 3.75 | 1.21 | 4.41 | 8.91 | 16.05 | 4.28 | 56.49 | 9.9 | 1.69 | 15.82 | 370 |
| 3 | 1 | 52 | 144 | 79 | 29.50 | 5.69 | 1.85 | 0.96 | 1.50 | 7.34 | 15.29 | 4.31 | 66.05 | 6.5 | 1.41 | 22.45 | 372 |
| 3 | 1 | 57 | 126 | 81 | 33.20 | 5.75 | 1.65 | 1.17 | 2.30 | 8.48 | 14 | 12.06 | 65.03 | 6.1 | 4.55 | 48.43 | 372 |
| 3 | 1 | 57 | 108 | 70 | 24.70 | 3.28 | 1.47 | 1.13 | 1.99 | 7.08 | 8.84 | 6.28 | 51.75 | 8.7 | 1.98 | 35.08 | 373 |
| 3 | 1 | 65 | 138 | 58 | 28.80 | 4.77 | 1.49 | 1.00 | 2.48 | 7.42 | 7.43 | 12.9 | 63.9 | 7 | 4.25 | 65.82 | 373 |
| 3 | 1 | 43 | 135 | 90 | 29.86 | 6.64 | 2.19 | 0.95 | 4.47 | 5.83 | 13.9 | 6.14 | 57.69 | 5.4 | 1.59 | 52.70 | 373 |
| 3 | 1 | 60 | 148 | 87 | 27.62 | 5.14 | 1.92 | 1.11 | 3.23 | 6.82 | 15.57 | 9.1 | 26.55 | 7.5 | 2.76 | 54.82 | 373 |
| 3 | 1 | 61 | 124 | 84 | 22.91 | 3.71 | 1.61 | 0.81 | 2.27 | 7.62 | 10.13 | 9.97 | 57.08 | 8.7 | 3.38 | 48.40 | 378 |
| 3 | 1 | 69 | 130 | 80 | 19.00 | 3.75 | 0.93 | 1.19 | 1.93 | 11 | 18.72 | 7.14 | 23.68 | 5.4 | 3.50 | 18.96 | 378 |
| 3 | 1 | 42 | 134 | 78 | 22.60 | 4.64 | 1.34 | 1.92 | 1.82 | 11.3 | 14.74 | 4.32 | 62.76 | 6.5 | 2.17 | 11.06 | 378 |
| 3 | 1 | 79 | 130 | 68 | 22.60 | 4.4 | 1.32 | 1.29 | 2.18 | 7.16 | 10.84 | 4.10 | 50.42 | 7.8 | 1.30 | 22.40 | 379 |
| 3 | 1 | 55 | 140 | 80 | 23.90 | 7.22 | 0.91 | 0.96 | 4.28 | 8.94 | 13.4 | 13.21 | 58.17 | 6.3 | 5.25 | 48.57 | 379 |
| 3 | 1 | 45 | 125 | 65 | 36.40 | 4.39 | 6.16 | 1.35 | 1.5 | 6.09 | 14.71 | 7.93 | 20.96 | 5.4 | 2.15 | 61.24 | 382 |
| 3 | 1 | 60 | 140 | 80 | 29.70 | 6.37 | 1.96 | 1.54 | 2.75 | 6.21 | 11.91 | 4.46 | 34.20 | 6.7 | 1.23 | 32.92 | 382 |
| 3 | 1 | 57 | 130 | 80 | 27.80 | 4.29 | 2.03 | 1.27 | 2.05 | 5.8 | 9.87 | 5.60 | 49.24 | 5.6 | 1.44 | 48.70 | 382 |
| 3 | 1 | 69 | 135 | 70 | 26.80 | 4.92 | 0.53 | 1.2 | 1.12 | 6.43 | 15.63 | 4.82 | 33.94 | 7.1 | 1.38 | 32.90 | 383 |
| 3 | 1 | 64 | 120 | 65 | 24.20 | 4.42 | 1.66 | 1.33 | 2.32 | 7.14 | 12.43 | 7.02 | 26.29 | 6.7 | 2.23 | 38.57 | 384 |
| 3 | 1 | 56 | 120 | 70 | 29.30 | 4.59 | 1.84 | 1.19 | 2.42 | 6.44 | 12.16 | 8.12 | 19.82 | 7.3 | 2.32 | 55.24 | 386 |
| 3 | 1 | 70 | 120 | 66 | 24.20 | 3.04 | 1.56 | 1.04 | 1.22 | 6.19 | 18.16 | 9.38 | 72.28 | 8.1 | 2.58 | 69.74 | 387 |
| 3 | 1 | 62 | 115 | 76 | 20.39 | 4.55 | 1.98 | 1.14 | 2.87 | 7.69 | 13.06 | 6.57 | 39.04 | 6.9 | 2.25 | 31.36 | 388 |
| 3 | 1 | 61 | 133 | 76 | 24.84 | 4.32 | 1.7 | 1.35 | 2.43 | 5.37 | 8.01 | 4.82 | 55.08 | 6.3 | 1.15 | 51.55 | 388 |
| 3 | 1 | 40 | 120 | 78 | 25.16 | 4.34 | 2.05 | 1.21 | 2.3 | 10.4 | 18.69 | 4.04 | 60.76 | 8.5 | 1.86 | 11.80 | 388 |
| 3 | 1 | 62 | 143 | 84 | 23.17 | 3.29 | 1.25 | 1.07 | 1.49 | 5.93 | 12.82 | 7.31 | 59.69 | 7.8 | 1.93 | 60.16 | 388 |
| 3 | 1 | 64 | 127 | 84 | 27.85 | 5.28 | 0.93 | 1.68 | 2.95 | 12.4 | 14.92 | 6.57 | 18.52 | 7.5 | 3.63 | 14.70 | 388 |
| 3 | 1 | 45 | 115 | 80 | 30.96 | 4.51 | 1.55 | 1.33 | 2.58 | 9.71 | 12.38 | 5.80 | 21.54 | 7.5 | 2.50 | 18.68 | 388 |
| 3 | 1 | 60 | 125 | 65 | 30.93 | 4.73 | 1.35 | 1.37 | 2.9 | 7.4 | 12.05 | 11.30 | 70.93 | 7.9 | 3.72 | 57.95 | 388 |
| 3 | 1 | 59 | 110 | 60 | 28.42 | 4.24 | 1.39 | 1.21 | 1.34 | 7.28 | 11.89 | 5.21 | 54.16 | 5.7 | 1.69 | 27.57 | 388 |
| 3 | 1 | 65 | 114 | 74 | 25.99 | 3.63 | 1 | 0.88 | 2.14 | 7.96 | 9.92 | 8.80 | 30.12 | 6.5 | 3.11 | 39.46 | 389 |
| 3 | 1 | 78 | 138 | 78 | 25.00 | 4.44 | 0.92 | 1.05 | 2.72 | 8.13 | 12.9 | 11.87 | 66.19 | 7.8 | 4.29 | 51.27 | 390 |
| 3 | 1 | 75 | 115 | 80 | 29.10 | 4.07 | 1.18 | 1.21 | 2 | 7.91 | 12.71 | 6.48 | 40.76 | 7.2 | 2.28 | 29.39 | 390 |
| 3 | 1 | 40 | 125 | 84 | 34.30 | 4.62 | 2.57 | 1.02 | 2.73 | 7.07 | 10.86 | 12.94 | 67.42 | 6.7 | 4.07 | 72.49 | 392 |
| 3 | 1 | 61 | 130 | 78 | 28.30 | 4.79 | 0.79 | 1.41 | 2.98 | 8.48 | 12.72 | 4.95 | 40.36 | 7.7 | 1.87 | 19.88 | 392 |
| 3 | 1 | 76 | 106 | 64 | 29.04 | 4.18 | 2.59 | 1.57 | 2.01 | 9.53 | 16.66 | 7.80 | 67.30 | 7.7 | 3.30 | 25.87 | 392 |
| 3 | 1 | 64 | 140 | 75 | 25.82 | 5.41 | 3.22 | 1.62 | 2.72 | 9.02 | 13.18 | 12.49 | 37.21 | 6.5 | 5.01 | 45.25 | 392 |
| 3 | 1 | 44 | 124 | 80 | 30.56 | 4.65 | 1.19 | 1.13 | 2.92 | 6.47 | 11.56 | 8.35 | 68.94 | 6.7 | 2.40 | 56.23 | 395 |
| 3 | 1 | 28 | 114 | 78 | 27.87 | 4.64 | 1.38 | 1.28 | 2.56 | 8.02 | 13.81 | 6.12 | 66.69 | 6 | 2.18 | 27.08 | 395 |
| 3 | 1 | 65 | 130 | 80 | 29.40 | 4.78 | 2.09 | 1.33 | 2.48 | 5.91 | 10.49 | 7.71 | 67.66 | 8.2 | 2.03 | 63.98 | 395 |
| 3 | 1 | 37 | 120 | 70 | 37.20 | 4.59 | 2.25 | 1.33 | 2.71 | 6.69 | 11.57 | 7.17 | 63.60 | 8.1 | 2.13 | 44.95 | 399 |
| 3 | 1 | 57 | 110 | 65 | 24.20 | 3.48 | 0.41 | 0.68 | 1.58 | 5.91 | 7.14 | 4.78 | 20.50 | 5.6 | 1.26 | 39.67 | 400 |
| 3 | 1 | 59 | 124 | 80 | 25.50 | 4.62 | 1.18 | 1.15 | 3.71 | 5.69 | 11.67 | 7.63 | 58.22 | 6.2 | 1.93 | 69.68 | 400 |
| 3 | 1 | 64 | 155 | 93 | 24.20 | 4.65 | 1.65 | 1.29 | 3.75 | 7.97 | 11.84 | 9.05 | 61.73 | 5.5 | 3.21 | 40.49 | 400 |
| 3 | 1 | 39 | 124 | 76 | 26.50 | 4.75 | 2.21 | 1 | 3.09 | 7.71 | 12.5 | 5.15 | 69.29 | 7 | 1.76 | 24.47 | 402 |
| 3 | 1 | 61 | 116 | 60 | 22.30 | 4.64 | 1.77 | 1.46 | 2.88 | 9.12 | 11.99 | 9.86 | 63.03 | 7.8 | 4.00 | 35.09 | 407 |
| 3 | 1 | 71 | 150 | 85 | 23.00 | 3.9 | 0.76 | 1.13 | 2.2 | 8.64 | 13.93 | 7.52 | 29.64 | 5.8 | 2.89 | 29.26 | 410 |
| 3 | 1 | 40 | 110 | 60 | 31.40 | 3.75 | 1.46 | 0.9 | 2.21 | 6.86 | 10.84 | 6.40 | 51.80 | 11.2 | 1.95 | 38.10 | 413 |
| 3 | 1 | 63 | 125 | 75 | 26.10 | 3.2 | 5.53 | 0.77 | 1.6 | 10.4 | 13.08 | 9.66 | 23.73 | 7.1 | 4.46 | 28.08 | 414 |
| 3 | 1 | 52 | 140 | 80 | 28.00 | 3.66 | 1.94 | 0.99 | 2.12 | 9.14 | 13.29 | 6.76 | 18.16 | 7.6 | 2.75 | 23.97 | 414 |
| 3 | 1 | 50 | 135 | 80 | 28.40 | 5.34 | 1.3 | 0.61 | 1.31 | 7.63 | 14.65 | 5.07 | 40.33 | 7.1 | 1.72 | 24.55 | 416 |
| 3 | 1 | 54 | 130 | 80 | 26.00 | 4.8 | 1.97 | 1.09 | 3.38 | 7.94 | 12.04 | 9.95 | 34.94 | 7.4 | 3.51 | 44.82 | 416 |
| 3 | 1 | 62 | 130 | 80 | 27.70 | 4.93 | 1.67 | 1.25 | 3.21 | 9.81 | 16.06 | 11.09 | 50.26 | 7.9 | 4.84 | 35.15 | 418 |
| 3 | 1 | 54 | 117 | 75 | 25.40 | 3.83 | 1.39 | 1.28 | 1.97 | 9.12 | 15.39 | 7.63 | 34.41 | 6.8 | 3.09 | 27.15 | 419 |
| 3 | 1 | 57 | 154 | 96 | 26.80 | 4.21 | 1.17 | 1.35 | 2.31 | 9.08 | 15.52 | 13.68 | 47.42 | 10.8 | 5.52 | 49.03 | 422 |
| 3 | 1 | 46 | 138 | 85 | 32.20 | 6.92 | 13.52 | 1.22 | 3.91 | 10.2 | 14.24 | 6.21 | 29.15 | 6 | 2.82 | 18.45 | 422 |
| 3 | 1 | 55 | 140 | 70 | 20.00 | 5.64 | 1.07 | 1.27 | 3.01 | 11.9 | 17.31 | 12.14 | 60.27 | 7.1 | 6.40 | 29.04 | 424 |
| 3 | 1 | 47 | 136 | 80 | 27.89 | 3.67 | 1.73 | 1.11 | 2.23 | 7.36 | 7.7 | 9.27 | 20.66 | 6.7 | 3.03 | 48.03 | 424 |
| 3 | 1 | 48 | 142 | 93 | 34.60 | 5.01 | 4.82 | 1.09 | 3.08 | 7.14 | 7.61 | 4.87 | 16.89 | 6 | 1.55 | 26.76 | 425 |
| 3 | 1 | 53 | 136 | 84 | 33.10 | 6.5 | 2.31 | 1.65 | 3.21 | 6.66 | 13.7 | 13.14 | 64.80 | 6.5 | 3.89 | 83.16 | 425 |
| 3 | 1 | 58 | 130 | 75 | 27.70 | 3.27 | 1.32 | 0.83 | 1.99 | 10.4 | 16.33 | 8.91 | 28.80 | 7.1 | 4.13 | 25.68 | 426 |
| 3 | 1 | 66 | 115 | 75 | 29.40 | 4.13 | 0.85 | 1.12 | 2.55 | 7.13 | 9.56 | 14.39 | 67.92 | 6.3 | 4.56 | 79.28 | 426 |
| 3 | 1 | 63 | 115 | 70 | 27.10 | 3.53 | 1.16 | 0.85 | 2.44 | 6.39 | 9.61 | 10.38 | 68.41 | 8.4 | 2.95 | 71.83 | 426 |
| 3 | 1 | 77 | 146 | 86 | 30.40 | 5.6 | 1.43 | 1.16 | 1.87 | 8.95 | 18.66 | 10.44 | 41.19 | 7.7 | 4.15 | 38.31 | 426 |
| 3 | 1 | 58 | 160 | 90 | 25.50 | 6.62 | 0.84 | 1.37 | 3.74 | 10.5 | 12.12 | 11.63 | 53.48 | 7.5 | 5.45 | 33.04 | 426 |
| 3 | 1 | 62 | 104 | 50 | 24.30 | 4.52 | 2.46 | 1.16 | 2.3 | 8.53 | 12.91 | 3.92 | 18.05 | 7.5 | 1.49 | 15.59 | 326 |
| 3 | 1 | 77 | 150 | 83 | 25.90 | 5.76 | 2.51 | 1.15 | 4.07 | 6.99 | 14.11 | 10.81 | 48.01 | 6 | 3.36 | 61.95 | 430 |
| 3 | 1 | 68 | 136 | 71 | 26.60 | 3.51 | 0.99 | 0.9 | 2.12 | 5.74 | 12.83 | 5.39 | 25.45 | 6.9 | 1.38 | 48.13 | 431 |
| 3 | 1 | 48 | 126 | 76 | 30.10 | 4.24 | 1.39 | 1.01 | 2.91 | 11.9 | 13.83 | 5.35 | 25.25 | 9.7 | 2.84 | 12.68 | 432 |
| 3 | 1 | 53 | 112 | 68 | 20.90 | 4.16 | 0.46 | 1.01 | 2.96 | 4.98 | 14.61 | 4.88 | 71.84 | 6.4 | 1.08 | 65.95 | 432 |
| 3 | 1 | 52 | 114 | 76 | 24.20 | 4.45 | 3.44 | 0.93 | 2.8 | 11 | 12.87 | 5.31 | 40.18 | 10.7 | 2.59 | 14.20 | 435 |
| 3 | 1 | 60 | 129 | 87 | 25.80 | 5.25 | 2.03 | 1.23 | 2.83 | 7.58 | 11.17 | 12.09 | 56.00 | 6.2 | 4.07 | 59.26 | 435 |
| 3 | 1 | 36 | 144 | 81 | 26.44 | 5.53 | 1.8 | 0.99 | 3.04 | 7.04 | 11.7 | 6.76 | 65.59 | 6.4 | 2.12 | 38.19 | 435 |
| 3 | 1 | 28 | 124 | 76 | 28.10 | 4.54 | 0.88 | 1.27 | 2.76 | 5.32 | 9.86 | 6.98 | 50.50 | 5.7 | 1.65 | 76.70 | 436 |
| 3 | 1 | 61 | 118 | 78 | 25.60 | 5.03 | 0.77 | 1.48 | 3.11 | 6.93 | 11.13 | 4.08 | 23.85 | 6.3 | 1.26 | 23.79 | 438 |
| 3 | 1 | 51 | 133 | 76 | 29.30 | 4.35 | 1.45 | 1.22 | 2.51 | 7.37 | 7.42 | 7.12 | 22.30 | 5.2 | 2.33 | 36.80 | 439 |
| 3 | 1 | 66 | 143 | 84 | 24.80 | 4.25 | 0.48 | 1.33 | 2.59 | 10.2 | 14.01 | 4.43 | 25.49 | 6.4 | 2.01 | 13.20 | 439 |
| 3 | 1 | 61 | 134 | 79 | 22.00 | 3.72 | 0.65 | 1.22 | 2.21 | 7.37 | 11.46 | 5.45 | 25.72 | 9.6 | 1.79 | 28.17 | 442 |
| 3 | 1 | 61 | 136 | 76 | 27.68 | 4.7 | 1.89 | 1.66 | 2.61 | 6.9 | 8.84 | 10.04 | 52.34 | 7.6 | 3.08 | 59.06 | 442 |
| 3 | 1 | 27 | 125 | 77 | 24.54 | 5.24 | 20.31 | 1.02 | 2 | 11.5 | 13.55 | 4.54 | 35.88 | 11 | 2.31 | 11.39 | 442 |
| 3 | 1 | 41 | 136 | 80 | 29.22 | 5.78 | 0.93 | 1.08 | 2.17 | 5.65 | 8.01 | 9.76 | 48.90 | 5.7 | 2.45 | 90.79 | 442 |
| 3 | 1 | 63 | 134 | 74 | 23.67 | 4.09 | 1.03 | 0.87 | 3.06 | 8.06 | 13.83 | 7.58 | 39.90 | 6.4 | 2.72 | 33.25 | 448 |
| 3 | 1 | 46 | 130 | 78 | 22.49 | 4.24 | 0.32 | 2.13 | 2.12 | 7.54 | 14.77 | 4.58 | 24.63 | 6.2 | 1.53 | 22.67 | 448 |
| 3 | 1 | 63 | 133 | 80 | 25.69 | 5.65 | 1.41 | 1.11 | 3.89 | 10.1 | 11 | 8.51 | 22.56 | 8.7 | 3.82 | 25.75 | 449 |
| 3 | 1 | 50 | 136 | 76 | 29.58 | 6.55 | 1.55 | 0.66 | 4.81 | 10.5 | 18.28 | 4.03 | 26.27 | 9.3 | 1.88 | 11.56 | 450 |
| 3 | 1 | 75 | 147 | 84 | 25.95 | 3.82 | 0.6 | 1.24 | 2.34 | 7.57 | 11.7 | 4.25 | 32.43 | 6.6 | 1.43 | 20.88 | 450 |
| 3 | 1 | 44 | 132 | 74 | 28.88 | 6.66 | 2.45 | 1.41 | 3.15 | 7.16 | 14.24 | 6.47 | 45.98 | 7 | 2.06 | 35.36 | 450 |
| 3 | 1 | 33 | 129 | 80 | 29.76 | 6.65 | 1.11 | 1.34 | 5.25 | 8.14 | 15.37 | 4.32 | 66.09 | 6.8 | 1.56 | 18.62 | 450 |
| 3 | 1 | 52 | 121 | 82 | 25.95 | 3.81 | 0.92 | 1.32 | 2.11 | 12 | 12.88 | 4.89 | 27.29 | 9.4 | 2.61 | 11.52 | 453 |
| 3 | 1 | 61 | 134 | 77 | 23.26 | 3.98 | 0.61 | 1.33 | 2.29 | 5.14 | 7.26 | 5.85 | 35.57 | 5.2 | 1.34 | 71.34 | 454 |
| 3 | 1 | 65 | 136 | 76 | 26.73 | 3.96 | 0.73 | 1.35 | 2.4 | 7.57 | 14.66 | 6.24 | 51.51 | 6.4 | 2.10 | 30.66 | 456 |
| 3 | 1 | 65 | 117 | 77 | 25.61 | 5.66 | 2.88 | 0.81 | 3.46 | 11.2 | 9.81 | 12.82 | 55.44 | 6.8 | 6.40 | 33.17 | 456 |
| 3 | 1 | 60 | 128 | 68 | 28.04 | 5.38 | 2.1 | 1.28 | 3.76 | 11.9 | 13.27 | 6.71 | 30.61 | 6.9 | 3.54 | 16.01 | 458 |
| 3 | 1 | 51 | 122 | 68 | 23.05 | 3.03 | 0.33 | 1.09 | 1.6 | 6.07 | 12.63 | 3.93 | 29.55 | 5.2 | 1.06 | 30.58 | 458 |
| 3 | 1 | 55 | 118 | 76 | 26.90 | 6.1 | 10.13 | 1.44 | 2.72 | 8.72 | 8.1 | 14.08 | 38.03 | 6 | 5.46 | 53.95 | 458 |
| 3 | 1 | 68 | 113 | 79 | 30.86 | 3.91 | 0.89 | 1.32 | 2.56 | 5.92 | 11.7 | 8.54 | 63.50 | 5.4 | 2.25 | 70.58 | 463 |
| 3 | 1 | 57 | 130 | 80 | 28.04 | 5.76 | 1 | 1.26 | 4.05 | 11.3 | 12.07 | 5.65 | 33.07 | 11.1 | 2.85 | 14.41 | 468 |
| 3 | 1 | 68 | 145 | 98 | 26.90 | 5.34 | 1.27 | 1.56 | 3.23 | 5.19 | 11.79 | 5.20 | 55.00 | 6.1 | 1.20 | 61.54 | 471 |
| 3 | 1 | 60 | 140 | 77 | 23.03 | 5.98 | 0.79 | 2.07 | 3.84 | 7.19 | 9.6 | 4.88 | 46.59 | 5.8 | 1.56 | 26.45 | 475 |
| 3 | 1 | 79 | 136 | 80 | 19.78 | 4.46 | 0.61 | 1.91 | 2.42 | 12.4 | 15.81 | 8.45 | 65.50 | 11.8 | 4.66 | 18.99 | 480 |
| 3 | 1 | 47 | 141 | 86 | 26.95 | 6.13 | 6.95 | 1.34 | 3.01 | 8.22 | 16.75 | 7.47 | 29.10 | 7.7 | 2.73 | 31.65 | 480 |
| 3 | 1 | 51 | 145 | 96 | 28.65 | 4.37 | 1.11 | 1.38 | 2.67 | 6.03 | 9.77 | 9.13 | 60.64 | 6.4 | 2.45 | 72.17 | 482 |
| 3 | 1 | 65 | 115 | 75 | 24.02 | 3.1 | 1.21 | 0.83 | 1.56 | 5.99 | 15.04 | 4.66 | 64.10 | 6.1 | 1.24 | 37.43 | 486 |
| 3 | 1 | 55 | 140 | 90 | 32.45 | 4.07 | 1.02 | 0.85 | 2.97 | 9.2 | 16.67 | 7.22 | 50.03 | 6.6 | 2.95 | 25.33 | 396 |
| 3 | 1 | 54 | 133 | 67 | 23.60 | 4.14 | 2.71 | 1 | 2.57 | 10.6 | 15.25 | 8.10 | 27.78 | 9.9 | 3.81 | 22.88 | 498 |
| 3 | 1 | 68 | 114 | 80 | 31.25 | 4.33 | 1.04 | 1.28 | 2.92 | 8.06 | 10.86 | 8.40 | 72.68 | 7.5 | 3.01 | 36.84 | 400 |
| 3 | 1 | 51 | 143 | 94 | 26.87 | 4.36 | 1 | 1.43 | 2.98 | 5.82 | 10.19 | 8.89 | 50.54 | 5.4 | 2.30 | 76.64 | 506 |
| 3 | 1 | 68 | 132 | 84 | 26.31 | 4.52 | 1 | 1.52 | 2.96 | 10.9 | 14.35 | 8.02 | 33.33 | 7.5 | 3.87 | 21.79 | 406 |
| 3 | 1 | 65 | 130 | 78 | 25.39 | 5.69 | 0.89 | 1.74 | 3.67 | 6.16 | 9.07 | 6.58 | 65.23 | 6.1 | 1.80 | 49.47 | 260 |
| 3 | 1 | 57 | 140 | 70 | 21.13 | 5.9 | 1.05 | 1.31 | 4.65 | 6.26 | 14.12 | 5.50 | 28.83 | 7.4 | 1.53 | 39.86 | 508 |
| 3 | 1 | 80 | 140 | 65 | 27.87 | 5.05 | 2.81 | 1.23 | 3.05 | 11.4 | 15.06 | 4.07 | 53.86 | 10.9 | 2.05 | 10.37 | 509 |
| 3 | 1 | 72 | 130 | 76 | 26.78 | 4.79 | 1.3 | 1.34 | 1 | 6.34 | 7.41 | 4.42 | 27.73 | 4.9 | 1.25 | 31.13 | 309 |
| 3 | 1 | 61 | 119 | 79 | 29.05 | 4.44 | 0.87 | 1.63 | 2.61 | 8.83 | 14.42 | 5.71 | 62.15 | 6.9 | 2.24 | 21.43 | 309 |
| 3 | 1 | 81 | 162 | 72 | 30.85 | 5.8 | 1.97 | 1.01 | 2.14 | 9.51 | 14.22 | 12.35 | 55.90 | 8.4 | 5.22 | 41.10 | 520 |
| 3 | 1 | 49 | 128 | 74 | 30.07 | 5.72 | 2.43 | 1.31 | 2.3 | 7.84 | 7.92 | 8.67 | 22.30 | 7.1 | 3.02 | 39.95 | 520 |
| 3 | 1 | 73 | 142 | 93 | 24.49 | 5.06 | 0.66 | 1.61 | 2.29 | 7.19 | 11.65 | 4.18 | 17.46 | 5.9 | 1.34 | 22.66 | 525 |
| 3 | 1 | 63 | 141 | 78 | 21.97 | 5.08 | 0.46 | 2.06 | 3.11 | 6.2 | 8.75 | 4.69 | 62.20 | 5.5 | 1.29 | 34.74 | 525 |
| 3 | 1 | 63 | 130 | 78 | 25.10 | 4.79 | 0.83 | 1.29 | 3.41 | 5.15 | 6.86 | 8.35 | 64.54 | 5.8 | 1.91 | 101.21 | 533 |
| 3 | 1 | 52 | 121 | 81 | 27.06 | 6.33 | 7.6 | 0.62 | 2.16 | 10.5 | 18 | 6.62 | 66.23 | 7.5 | 3.09 | 18.94 | 533 |
| 3 | 1 | 67 | 140 | 72 | 27.73 | 4.35 | 2.85 | 1.37 | 2.87 | 10.5 | 17.33 | 5.34 | 17.91 | 8.8 | 2.48 | 15.37 | 535 |
| 3 | 1 | 38 | 136 | 90 | 27.28 | 4.79 | 1.43 | 1.17 | 3.11 | 9.35 | 14.25 | 7.97 | 56.66 | 8.6 | 3.31 | 27.25 | 440 |
| 3 | 1 | 56 | 138 | 86 | 36.23 | 5.61 | 2.36 | 1.43 | 3.55 | 9.51 | 16.56 | 5.89 | 56.07 | 7.9 | 2.49 | 19.60 | 446 |
| 3 | 1 | 68 | 144 | 95 | 20.62 | 4.27 | 0.59 | 1.44 | 1.61 | 8.41 | 10.38 | 4.83 | 52.06 | 7 | 1.81 | 19.67 | 548 |
| 3 | 1 | 66 | 116 | 76 | 29.75 | 4.33 | 1.43 | 1.09 | 3.06 | 8.9 | 16.15 | 6.42 | 29.57 | 6.8 | 2.54 | 23.78 | 548 |
| 3 | 1 | 72 | 131 | 77 | 29.87 | 3.48 | 0.96 | 1.28 | 1.98 | 7.45 | 12.48 | 4.84 | 28.23 | 6.3 | 1.60 | 24.51 | 551 |
| 3 | 1 | 67 | 122 | 64 | 32.79 | 4.03 | 1.96 | 1.06 | 2.57 | 9.32 | 13.04 | 11.33 | 52.63 | 10.3 | 4.69 | 38.93 | 553 |
| 3 | 1 | 48 | 132 | 86 | 31.24 | 5.05 | 1.38 | 1.17 | 3.69 | 9 | 11.63 | 6.65 | 17.81 | 7.8 | 2.66 | 24.18 | 553 |
| 3 | 1 | 59 | 125 | 77 | 25.51 | 4.81 | 1.42 | 1.11 | 3.43 | 6.77 | 8.9 | 5.62 | 27.25 | 6.5 | 1.69 | 34.37 | 560 |
| 3 | 1 | 67 | 143 | 75 | 25.91 | 6.08 | 2.58 | 1.22 | 4.57 | 5.54 | 7.8 | 5.45 | 69.38 | 5.8 | 1.34 | 53.43 | 560 |
| 3 | 1 | 61 | 131 | 77 | 29.41 | 5.27 | 4.1 | 1.53 | 2.63 | 9.7 | 14.58 | 8.12 | 23.53 | 9.2 | 3.50 | 26.19 | 562 |
| 3 | 1 | 76 | 132 | 77 | 26.44 | 4.26 | 0.95 | 1.55 | 2.61 | 8.43 | 11.66 | 5.88 | 17.45 | 7.1 | 2.20 | 23.85 | 568 |
| 3 | 1 | 64 | 120 | 70 | 28.98 | 4.4 | 1.43 | 1.06 | 3 | 5.48 | 6.93 | 7.34 | 73.46 | 5.6 | 1.79 | 74.14 | 570 |
| 3 | 1 | 55 | 111 | 71 | 29.05 | 4.44 | 1.75 | 1.17 | 2.59 | 7.85 | 8.18 | 7.54 | 67.56 | 7.2 | 2.63 | 34.67 | 570 |
| 3 | 1 | 51 | 112 | 68 | 31.53 | 5.84 | 2.57 | 0.92 | 2.22 | 5.98 | 11.22 | 12.58 | 34.90 | 5.4 | 3.34 | 101.45 | 579 |
| 3 | 1 | 70 | 134 | 76 | 27.18 | 2.98 | 0.53 | 1.15 | 1.38 | 7.75 | 13.53 | 6.70 | 25.31 | 6.6 | 2.31 | 31.53 | 603 |
| 3 | 1 | 47 | 142 | 87 | 31.02 | 5.27 | 3.75 | 1.3 | 3.01 | 10.3 | 11.07 | 8.73 | 18.68 | 8 | 4.00 | 25.68 | 507 |
| 3 | 1 | 60 | 127 | 67 | 31.83 | 5.66 | 2.41 | 1.3 | 3.26 | 6.67 | 12.47 | 9.65 | 67.73 | 6.9 | 2.86 | 60.88 | 509 |
| 3 | 1 | 51 | 136 | 70 | 30.80 | 3.17 | 1.63 | 0.95 | 1.73 | 9.65 | 16.33 | 9.04 | 54.31 | 8.3 | 3.88 | 29.40 | 521 |
| 3 | 1 | 78 | 133 | 73 | 25.67 | 4.28 | 1.16 | 1.4 | 2.56 | 6.17 | 11.87 | 9.84 | 55.33 | 6.4 | 2.70 | 73.71 | 580 |
| 3 | 1 | 44 | 131 | 73 | 25.54 | 5.03 | 1.33 | 1.42 | 2.13 | 9.05 | 14.83 | 11.18 | 43.88 | 6.9 | 4.50 | 40.29 | 606 |
| 3 | 1 | 64 | 121 | 71 | 34.68 | 6.11 | 1.01 | 1.04 | 2.82 | 7.07 | 11.77 | 8.22 | 60.37 | 6.5 | 2.58 | 46.05 | 606 |
| 3 | 1 | 63 | 132 | 72 | 30.48 | 3.49 | 1.31 | 0.93 | 2 | 6.41 | 8.48 | 9.38 | 41.60 | 6.2 | 2.67 | 64.47 | 387 |
| 3 | 2 | 63 | 163 | 88 | 21.30 | 8.07 | 1.43 | 1.18 | 5.00 | 6.85 | 16.38 | 14.07 | 64.1 | 8.6 | 4.28 | 84.00 | 364 |
| 3 | 2 | 51 | 139 | 88 | 28.10 | 5.69 | 4.96 | 1.07 | 3.66 | 6.03 | 11.86 | 13.11 | 62.97 | 8.6 | 3.51 | 103.64 | 364 |
| 3 | 2 | 40 | 124 | 88 | 29.90 | 4.28 | 3.18 | 0.72 | 2.80 | 5.98 | 11.28 | 14.1 | 50.1 | 8.6 | 3.75 | 113.71 | 364 |
| 3 | 2 | 57 | 127 | 74 | 27.60 | 5.57 | 1.51 | 1.28 | 3.44 | 7.25 | 18.38 | 3.99 | 69.86 | 7.9 | 1.29 | 21.28 | 365 |
| 3 | 2 | 47 | 131 | 92 | 27.50 | 5.62 | 2.98 | 1.25 | 3.73 | 6.67 | 12.71 | 12.46 | 73.79 | 9.6 | 3.69 | 78.61 | 365 |
| 3 | 2 | 53 | 106 | 64 | 24.40 | 4.81 | 1.23 | 1.28 | 3.19 | 6.47 | 12.4 | 9.04 | 28 | 9.6 | 2.60 | 60.88 | 365 |
| 3 | 2 | 46 | 107 | 61 | 25.60 | 4.47 | 0.90 | 1.05 | 3.13 | 7.41 | 15.4 | 10.48 | 38.33 | 9.6 | 3.45 | 53.61 | 366 |
| 3 | 2 | 55 | 118 | 75 | 25.40 | 4.03 | 1.98 | 1.45 | 2.11 | 7.17 | 11.73 | 4 | 69.99 | 9.6 | 1.27 | 21.80 | 367 |
| 3 | 2 | 50 | 124 | 81 | 24.10 | 5.91 | 2.36 | 1.56 | 3.72 | 7.55 | 12.89 | 6.38 | 67.09 | 6.8 | 2.14 | 31.51 | 367 |
| 3 | 2 | 56 | 132 | 83 | 30.70 | 6.06 | 2.02 | 1.13 | 4.11 | 7.27 | 11.36 | 11.59 | 48.8 | 6.8 | 3.74 | 61.49 | 367 |
| 3 | 2 | 46 | 143 | 95 | 29.70 | 5.17 | 1.27 | 1.54 | 3.10 | 5.61 | 11.5 | 10.59 | 39.5 | 7.1 | 2.64 | 100.38 | 367 |
| 3 | 2 | 54 | 156 | 98 | 26.06 | 4.73 | 1.74 | 1.02 | 3.00 | 8.64 | 14.32 | 9.81 | 39.42 | 6.5 | 3.77 | 38.17 | 367 |
| 3 | 2 | 54 | 138 | 91 | 32.70 | 5.79 | 3.25 | 0.97 | 3.99 | 6.81 | 15.08 | 6.46 | 62.38 | 6.7 | 1.96 | 39.03 | 367 |
| 3 | 2 | 54 | 131 | 82 | 32.30 | 4.50 | 2.07 | 1.12 | 3.03 | 10.1 | 15.04 | 9.57 | 53.59 | 6 | 4.28 | 29.18 | 368 |
| 3 | 2 | 55 | 132 | 75 | 27.70 | 5.16 | 1.73 | 1.40 | 3.30 | 6.66 | 14 | 6.74 | 57.95 | 6.5 | 2.00 | 42.66 | 369 |
| 3 | 2 | 65 | 152 | 76 | 28.60 | 6.14 | 2.94 | 1.34 | 4.23 | 8.43 | 17.41 | 2.28 | 61.08 | 6.4 | 0.85 | 9.25 | 370 |
| 3 | 2 | 54 | 148 | 85 | 29.20 | 5.15 | 3.74 | 0.99 | 3.36 | 8.49 | 10.57 | 12.69 | 53.4 | 10.2 | 4.79 | 50.86 | 370 |
| 3 | 2 | 61 | 136 | 75 | 25.50 | 5.99 | 2.50 | 1.43 | 3.84 | 7.69 | 11.72 | 4.77 | 56.5 | 6.6 | 1.63 | 22.77 | 370 |
| 3 | 2 | 62 | 159 | 94 | 24.10 | 5.38 | 1.70 | 1.12 | 3.77 | 8.9 | 13.86 | 11.31 | 48.02 | 7.5 | 4.47 | 41.89 | 371 |
| 3 | 2 | 49 | 141 | 95 | 27.10 | 4.85 | 1.31 | 0.80 | 3.55 | 8.55 | 14.16 | 14.58 | 73.33 | 11.5 | 5.54 | 57.74 | 372 |
| 3 | 2 | 62 | 173 | 95 | 27.05 | 6.30 | 1.81 | 1.13 | 4.15 | 7.65 | 14.29 | 9.31 | 64.83 | 7.5 | 3.17 | 44.87 | 375 |
| 3 | 2 | 62 | 144 | 97 | 24.03 | 6.24 | 2.27 | 1.56 | 3.98 | 7.28 | 16.44 | 7.89 | 54.39 | 7.5 | 2.55 | 41.75 | 375 |
| 3 | 2 | 62 | 137 | 66 | 24.46 | 5.58 | 4.77 | 1.24 | 3.37 | 7.56 | 14.13 | 11.14 | 57.56 | 7.5 | 3.74 | 54.88 | 377 |
| 3 | 2 | 45 | 129 | 71 | 23.52 | 5.52 | 2.38 | 1.29 | 3.36 | 6.4 | 13.92 | 10.9 | 56.15 | 7.5 | 3.10 | 75.17 | 377 |
| 3 | 2 | 49 | 127 | 73 | 23.87 | 7.66 | 6.43 | 1.41 | 4.03 | 7.16 | 17.26 | 13.02 | 43.9 | 7.5 | 4.14 | 71.15 | 377 |
| 3 | 2 | 61 | 129 | 74 | 23.59 | 7.66 | 2.98 | 1.70 | 4.43 | 5.28 | 11.16 | 8.24 | 38.4 | 7.5 | 1.93 | 92.58 | 377 |
| 3 | 2 | 50 | 123 | 73 | 31.31 | 5.67 | 0.96 | 1.98 | 3.31 | 6.43 | 12.05 | 14.41 | 62.51 | 9.8 | 4.12 | 98.36 | 378 |
| 3 | 2 | 39 | 141 | 93 | 31.48 | 6.01 | 1.88 | 1.56 | 3.50 | 6.38 | 13.91 | 12.65 | 61.9 | 7.6 | 3.59 | 87.85 | 378 |
| 3 | 2 | 59 | 120 | 80 | 23.30 | 5.7 | 1.81 | 1.76 | 2.6 | 5.72 | 10.7 | 5.24 | 53.08 | 8.6 | 1.33 | 47.21 | 378 |
| 3 | 2 | 40 | 124 | 75 | 21.90 | 4.65 | 2.65 | 1.6 | 2.28 | 7.06 | 10.86 | 4.68 | 40.33 | 7.1 | 1.47 | 26.29 | 378 |
| 3 | 2 | 58 | 120 | 75 | 26.10 | 4.41 | 1.81 | 1.14 | 2.4 | 6.63 | 11.76 | 10.30 | 25.53 | 8 | 3.04 | 65.81 | 378 |
| 3 | 2 | 57 | 145 | 90 | 31.60 | 5.55 | 1.57 | 1.25 | 3.42 | 8.61 | 13.97 | 8.59 | 29.39 | 9.5 | 3.29 | 33.62 | 380 |
| 3 | 2 | 58 | 120 | 75 | 21.00 | 7.25 | 1.72 | 1.56 | 3.86 | 7.25 | 10.88 | 5.01 | 18.03 | 5.5 | 1.61 | 26.72 | 380 |
| 3 | 2 | 66 | 114 | 78 | 27.90 | 6.02 | 2.17 | 1.95 | 3.07 | 9.55 | 17.66 | 4.13 | 66.97 | 6.5 | 1.75 | 13.65 | 380 |
| 3 | 2 | 65 | 113 | 75 | 28.90 | 7.25 | 1.15 | 0.97 | 3.88 | 8.32 | 11.86 | 9.98 | 42.79 | 11.2 | 3.69 | 41.41 | 380 |
| 3 | 2 | 55 | 115 | 80 | 21.90 | 3.9 | 1.73 | 0.61 | 1 | 5.91 | 8.22 | 4.43 | 60.70 | 5.9 | 1.16 | 36.76 | 380 |
| 3 | 2 | 55 | 130 | 75 | 24.20 | 5.86 | 2 | 1.68 | 3 | 12.4 | 15.09 | 6.96 | 25.67 | 10.2 | 3.85 | 15.59 | 382 |
| 3 | 2 | 52 | 110 | 70 | 23.70 | 5.65 | 0.75 | 2.1 | 2.53 | 12 | 14.24 | 4.31 | 23.89 | 6.8 | 2.30 | 10.15 | 383 |
| 3 | 2 | 47 | 140 | 85 | 29.90 | 5.27 | 2.36 | 1.35 | 2.89 | 5.31 | 7.8 | 7.29 | 62.75 | 6.8 | 1.72 | 80.55 | 383 |
| 3 | 2 | 53 | 125 | 75 | 31.60 | 5.08 | 1.63 | 1.16 | 2.8 | 6.79 | 11.45 | 7.84 | 42.52 | 6.5 | 2.37 | 47.66 | 383 |
| 3 | 2 | 61 | 138 | 80 | 23.00 | 4.74 | 0.54 | 2.25 | 1.97 | 7.27 | 14.02 | 4.67 | 23.57 | 7.8 | 1.51 | 24.77 | 384 |
| 3 | 2 | 40 | 124 | 86 | 23.20 | 3.55 | 0.42 | 1.36 | 1.66 | 7.41 | 11.48 | 5.59 | 48.63 | 6.4 | 1.84 | 28.59 | 386 |
| 3 | 2 | 59 | 110 | 65 | 23.30 | 5.14 | 1.03 | 1.64 | 2.72 | 11.8 | 13.29 | 7.52 | 25.55 | 5.9 | 3.93 | 18.23 | 387 |
| 3 | 2 | 51 | 170 | 100 | 27.20 | 3.53 | 1.08 | 1.04 | 1.57 | 7.01 | 14.13 | 5.97 | 42.28 | 6 | 1.86 | 34.02 | 387 |
| 3 | 2 | 72 | 150 | 83 | 23.60 | 6.04 | 1.47 | 0.64 | 2.86 | 6.56 | 14.02 | 10.74 | 55.40 | 6.3 | 3.13 | 70.20 | 387 |
| 3 | 2 | 65 | 136 | 71 | 25.63 | 5.04 | 1.06 | 1.34 | 2.77 | 6.38 | 12.59 | 8.34 | 58.05 | 6.5 | 2.36 | 57.92 | 387 |
| 3 | 2 | 63 | 126 | 76 | 25.72 | 5.12 | 2.24 | 1.23 | 2.61 | 7.44 | 13.83 | 7.01 | 57.45 | 6.4 | 2.32 | 35.58 | 388 |
| 3 | 2 | 69 | 139 | 90 | 21.34 | 6.46 | 0.94 | 1.96 | 2.64 | 6.07 | 11.3 | 4.92 | 29.94 | 9 | 1.33 | 38.29 | 388 |
| 3 | 2 | 63 | 130 | 75 | 31.85 | 6.56 | 4.58 | 1.55 | 2.99 | 8.63 | 16.67 | 7.84 | 46.25 | 6.9 | 3.01 | 30.57 | 388 |
| 3 | 2 | 63 | 140 | 80 | 27.50 | 5.1 | 0.53 | 2.24 | 2.19 | 10.9 | 14.07 | 4.61 | 51.25 | 5.8 | 2.24 | 12.43 | 388 |
| 3 | 2 | 60 | 114 | 65 | 22.30 | 5.04 | 0.42 | 2.13 | 1.91 | 5.18 | 12.55 | 5.54 | 72.36 | 4.2 | 1.28 | 65.95 | 388 |
| 3 | 2 | 60 | 155 | 93 | 24.77 | 5.79 | 2.15 | 0.61 | 2.86 | 5.1 | 11.05 | 5.65 | 60.12 | 8.2 | 1.28 | 70.63 | 389 |
| 3 | 2 | 60 | 120 | 70 | 23.95 | 3.54 | 0.65 | 1.55 | 1.48 | 5.93 | 9.93 | 4.50 | 18.45 | 6.9 | 1.19 | 37.04 | 389 |
| 3 | 2 | 65 | 118 | 68 | 25.92 | 6.14 | 1.52 | 1.58 | 3.68 | 10.2 | 13.83 | 7.34 | 24.10 | 8.9 | 3.31 | 22.08 | 389 |
| 3 | 2 | 57 | 125 | 75 | 26.02 | 4.31 | 0.61 | 1.73 | 2.15 | 8.73 | 12.52 | 4.56 | 30.14 | 6.6 | 1.77 | 17.44 | 390 |
| 3 | 2 | 76 | 125 | 65 | 23.90 | 5.01 | 1.51 | 1.36 | 2.49 | 6.38 | 9.22 | 4.88 | 26.89 | 6.5 | 1.38 | 33.89 | 390 |
| 3 | 2 | 65 | 110 | 60 | 28.98 | 4.82 | 2.73 | 1.44 | 2.57 | 6.55 | 11.51 | 4.74 | 44.30 | 6.2 | 1.38 | 31.08 | 392 |
| 3 | 2 | 63 | 128 | 78 | 24.00 | 5.48 | 1.28 | 0.88 | 2.56 | 6 | 10.68 | 4.41 | 48.61 | 5.9 | 1.18 | 35.28 | 393 |
| 3 | 2 | 73 | 109 | 75 | 22.66 | 5.52 | 1.82 | 1.51 | 2.84 | 7 | 10.38 | 7.50 | 51.06 | 6.5 | 2.33 | 42.86 | 393 |
| 3 | 2 | 48 | 115 | 70 | 25.70 | 3.97 | 0.72 | 1.38 | 2.15 | 8.46 | 14.2 | 12.62 | 58.20 | 6.5 | 4.75 | 50.89 | 396 |
| 3 | 2 | 52 | 125 | 70 | 27.30 | 5.75 | 4.69 | 1.81 | 2 | 9.6 | 11.78 | 9.45 | 52.18 | 8.2 | 4.03 | 30.98 | 396 |
| 3 | 2 | 66 | 126 | 78 | 25.00 | 4.23 | 1.01 | 1.57 | 1.89 | 5.51 | 8.62 | 8.66 | 65.27 | 5 | 2.12 | 86.17 | 398 |
| 3 | 2 | 66 | 160 | 80 | 28.60 | 5.39 | 3.75 | 1.33 | 2.89 | 8.93 | 14.43 | 5.79 | 73.24 | 7 | 2.30 | 21.33 | 398 |
| 3 | 2 | 48 | 145 | 85 | 26.60 | 3.61 | 2.17 | 0.98 | 1.89 | 6.9 | 10.9 | 14.35 | 65.00 | 6.8 | 4.40 | 84.41 | 399 |
| 3 | 2 | 61 | 140 | 80 | 26.80 | 4.87 | 2.47 | 1.38 | 2.48 | 6.98 | 18.06 | 10.35 | 53.66 | 8.5 | 3.21 | 59.48 | 399 |
| 3 | 2 | 50 | 125 | 70 | 27.90 | 4.17 | 1.82 | 1.15 | 2.05 | 6.49 | 13.35 | 7.93 | 65.20 | 6.3 | 2.29 | 53.04 | 399 |
| 3 | 2 | 64 | 120 | 75 | 27.30 | 5.19 | 2.23 | 1.42 | 3.07 | 11.4 | 17.13 | 4.50 | 72.42 | 11.9 | 2.28 | 11.42 | 399 |
| 3 | 2 | 61 | 124 | 67 | 28.50 | 4.25 | 1.73 | 1.39 | 1.93 | 6.04 | 11.46 | 10.51 | 69.96 | 6.9 | 2.82 | 82.76 | 399 |
| 3 | 2 | 65 | 135 | 75 | 22.00 | 5.76 | 0.82 | 1.38 | 4.29 | 12.4 | 14.88 | 4.34 | 58.06 | 6.5 | 2.39 | 9.77 | 399 |
| 3 | 2 | 59 | 111 | 67 | 23.90 | 8 | 4.34 | 1.69 | 5.86 | 6.44 | 9.51 | 4.14 | 27.99 | 6.6 | 1.18 | 28.16 | 399 |
| 3 | 2 | 65 | 124 | 87 | 31.30 | 4.91 | 2.18 | 1.13 | 3.73 | 11.6 | 15.92 | 8.24 | 44.41 | 11.7 | 4.24 | 20.42 | 400 |
| 3 | 2 | 60 | 120 | 70 | 24.90 | 4.76 | 0.8 | 1.23 | 3.86 | 5.61 | 7.53 | 9.89 | 27.89 | 7 | 2.47 | 93.74 | 400 |
| 3 | 2 | 58 | 136 | 87 | 33.60 | 6.51 | 2.39 | 1.34 | 6 | 7.69 | 13.72 | 7.31 | 65.48 | 7.6 | 2.50 | 34.89 | 402 |
| 3 | 2 | 57 | 117 | 70 | 25.90 | 4.64 | 1.4 | 1.24 | 3.23 | 5.28 | 6.91 | 9.67 | 64.81 | 7.8 | 2.27 | 108.65 | 407 |
| 3 | 2 | 56 | 145 | 77 | 34.20 | 6.4 | 1.14 | 1.43 | 3.99 | 7.13 | 11.63 | 6.17 | 49.30 | 9.3 | 1.96 | 33.99 | 409 |
| 3 | 2 | 70 | 134 | 76 | 29.50 | 7 | 2.14 | 1.4 | 5.26 | 8.09 | 15.42 | 8.96 | 44.09 | 7.2 | 3.22 | 39.04 | 410 |
| 3 | 2 | 60 | 140 | 80 | 32.40 | 4.84 | 1.16 | 1.15 | 3.06 | 7.17 | 11.6 | 9.70 | 25.19 | 6.3 | 3.09 | 52.86 | 412 |
| 3 | 2 | 55 | 110 | 60 | 26.00 | 4.61 | 1.14 | 1.6 | 2.37 | 5.67 | 8.81 | 9.38 | 62.77 | 5.8 | 2.36 | 86.45 | 412 |
| 3 | 2 | 63 | 100 | 70 | 22.30 | 4.75 | 0.76 | 1.8 | 2.68 | 10.8 | 14.44 | 4.75 | 50.78 | 6.2 | 2.28 | 12.98 | 413 |
| 3 | 2 | 39 | 114 | 70 | 28.20 | 6.77 | 4.94 | 1.42 | 3.86 | 7.03 | 7.86 | 10.09 | 40.31 | 6.3 | 3.15 | 57.17 | 414 |
| 3 | 2 | 66 | 160 | 80 | 28.50 | 5.29 | 1.12 | 1.41 | 3.6 | 8.83 | 15.34 | 8.75 | 67.76 | 7.3 | 3.43 | 32.83 | 414 |
| 3 | 2 | 55 | 110 | 70 | 24.50 | 5.74 | 2.3 | 1.27 | 4.03 | 8.01 | 14.63 | 10.99 | 32.21 | 6.6 | 3.91 | 48.74 | 418 |
| 3 | 2 | 66 | 135 | 70 | 21.70 | 5.53 | 0.59 | 1.98 | 3.13 | 6.7 | 17.03 | 5.21 | 54.64 | 6.7 | 1.55 | 32.56 | 419 |
| 3 | 2 | 65 | 120 | 70 | 23.50 | 4.26 | 0.85 | 1.09 | 2.53 | 7.02 | 8.43 | 9.13 | 38.68 | 7.5 | 2.85 | 51.88 | 419 |
| 3 | 2 | 64 | 147 | 80 | 28.00 | 6.22 | 2.88 | 1.35 | 4.39 | 8.27 | 11.82 | 8.60 | 71.95 | 7.9 | 3.16 | 36.06 | 419 |
| 3 | 2 | 63 | 150 | 80 | 31.20 | 4.65 | 1.58 | 1.16 | 2.99 | 6.95 | 11.23 | 13.58 | 64.25 | 6.4 | 4.19 | 78.72 | 420 |
| 3 | 2 | 53 | 136 | 75 | 24.60 | 4.94 | 1.45 | 1.39 | 3.02 | 7.41 | 13.69 | 5.44 | 72.60 | 8.4 | 1.79 | 27.83 | 422 |
| 3 | 2 | 65 | 143 | 85 | 25.97 | 4.13 | 1.84 | 1.24 | 2.36 | 10.5 | 11.65 | 6.77 | 17.58 | 9.9 | 3.16 | 19.37 | 426 |
| 3 | 2 | 68 | 135 | 75 | 21.90 | 4.55 | 1.75 | 1.66 | 2.77 | 10.3 | 16.55 | 8.24 | 51.16 | 7.5 | 3.77 | 24.24 | 426 |
| 3 | 2 | 54 | 165 | 90 | 30.80 | 5.54 | 2.59 | 1.13 | 3.53 | 9.89 | 18.59 | 9.34 | 56.80 | 7.4 | 4.11 | 29.23 | 427 |
| 3 | 2 | 66 | 180 | 95 | 26.00 | 6.35 | 1.61 | 1.47 | 4.62 | 5.9 | 17.01 | 11.53 | 43.30 | 6.7 | 3.02 | 96.08 | 337 |
| 3 | 2 | 80 | 170 | 90 | 26.30 | 4.47 | 0.94 | 1.67 | 2.39 | 6.75 | 16.05 | 7.83 | 59.00 | 7.7 | 2.35 | 48.18 | 430 |
| 3 | 2 | 74 | 154 | 82 | 25.00 | 5.64 | 0.9 | 1.64 | 3.76 | 7.75 | 12.91 | 8.72 | 49.32 | 9.7 | 3.00 | 41.04 | 431 |
| 3 | 2 | 65 | 142 | 84 | 26.00 | 3.71 | 0.81 | 1.25 | 2.07 | 5.65 | 10.48 | 11.09 | 56.13 | 6.2 | 2.78 | 103.16 | 432 |
| 3 | 2 | 52 | 160 | 92 | 39.30 | 4.21 | 0.56 | 1.31 | 2.85 | 5.81 | 9.48 | 8.51 | 66.78 | 6.2 | 2.20 | 73.68 | 433 |
| 3 | 2 | 66 | 132 | 75 | 22.00 | 5.45 | 2.46 | 1.29 | 2.83 | 6.82 | 14.22 | 8.12 | 64.45 | 6.5 | 2.46 | 48.92 | 433 |
| 3 | 2 | 52 | 131 | 84 | 29.20 | 5.21 | 2.4 | 1.24 | 2.89 | 8.71 | 14.12 | 5.22 | 24.40 | 7.4 | 2.02 | 20.04 | 436 |
| 3 | 2 | 62 | 147 | 85 | 23.40 | 4.41 | 0.52 | 1.48 | 2.63 | 5.39 | 7.85 | 7.73 | 51.60 | 5 | 1.85 | 81.80 | 436 |
| 3 | 2 | 64 | 139 | 90 | 31.30 | 4.39 | 1.81 | 1.01 | 2.93 | 7.49 | 11.05 | 11.78 | 50.40 | 6.8 | 3.92 | 59.05 | 439 |
| 3 | 2 | 63 | 132 | 79 | 24.90 | 4.33 | 1.6 | 1.13 | 2.4 | 7.29 | 11.89 | 5.81 | 60.18 | 6.8 | 1.88 | 30.66 | 441 |
| 3 | 2 | 68 | 165 | 74 | 23.40 | 3.75 | 1.03 | 1.07 | 2.24 | 6.66 | 9.77 | 11.25 | 36.14 | 6.3 | 3.33 | 71.20 | 441 |
| 3 | 2 | 57 | 121 | 76 | 26.99 | 5.21 | 1.56 | 1.56 | 3.34 | 12.3 | 15.8 | 11.30 | 36.30 | 8.9 | 6.16 | 25.80 | 442 |
| 3 | 2 | 61 | 137 | 82 | 22.31 | 5.47 | 1.61 | 1.02 | 3.72 | 12.2 | 15.26 | 4.11 | 18.03 | 8.9 | 2.23 | 9.43 | 442 |
| 3 | 2 | 70 | 121 | 67 | 30.15 | 5.37 | 2.02 | 0.97 | 3.63 | 7.12 | 11.59 | 11.26 | 69.97 | 6.5 | 3.56 | 62.21 | 443 |
| 3 | 2 | 63 | 142 | 82 | 21.79 | 6.88 | 1.64 | 2.19 | 4.44 | 5.41 | 11.64 | 7.03 | 37.53 | 5.6 | 1.69 | 73.61 | 443 |
| 3 | 2 | 61 | 122 | 72 | 18.36 | 5.29 | 3.53 | 1.41 | 2.52 | 7.01 | 12.64 | 6.23 | 69.17 | 6.2 | 1.94 | 35.50 | 449 |
| 3 | 2 | 63 | 133 | 73 | 31.24 | 5.29 | 1.75 | 0.93 | 1.92 | 8.66 | 14.55 | 7.54 | 60.45 | 7.8 | 2.90 | 29.22 | 452 |
| 3 | 2 | 25 | 110 | 70 | 16.80 | 4.7 | 0.61 | 1.73 | 2.72 | 9.8 | 14.38 | 4.66 | 18.71 | 6.3 | 2.03 | 14.79 | 454 |
| 3 | 2 | 74 | 133 | 79 | 22.83 | 6.5 | 1.55 | 1.53 | 4.11 | 8.91 | 17.17 | 4.57 | 63.33 | 8.1 | 1.81 | 16.89 | 456 |
| 3 | 2 | 58 | 115 | 81 | 24.88 | 6.5 | 1.88 | 1.45 | 4.92 | 5.47 | 11.44 | 5.73 | 35.38 | 6.2 | 1.39 | 58.17 | 456 |
| 3 | 2 | 44 | 133 | 75 | 36.00 | 4.23 | 1.85 | 1.24 | 2.54 | 7.69 | 10.26 | 4.62 | 58.37 | 6.4 | 1.58 | 22.05 | 458 |
| 3 | 2 | 61 | 137 | 82 | 26.04 | 5.56 | 3.68 | 1.6 | 2.84 | 8.06 | 15.97 | 11.81 | 51.41 | 7.2 | 4.23 | 51.80 | 458 |
| 3 | 2 | 63 | 131 | 79 | 29.43 | 6.12 | 1.55 | 1.25 | 4.79 | 7.1 | 13.83 | 7.90 | 49.81 | 6.6 | 2.49 | 43.89 | 462 |
| 3 | 2 | 52 | 139 | 87 | 29.00 | 5.25 | 0.95 | 1.65 | 3.6 | 9.19 | 13.29 | 8.28 | 60.02 | 6.3 | 3.38 | 29.10 | 462 |
| 3 | 2 | 45 | 109 | 63 | 28.67 | 4.62 | 2.59 | 1.45 | 2.44 | 5 | 11.87 | 14.09 | 65.99 | 5.9 | 3.13 | 187.87 | 462 |
| 3 | 2 | 63 | 129 | 77 | 23.73 | 7.12 | 1.14 | 1.41 | 5.08 | 11 | 13.94 | 5.89 | 31.01 | 8.9 | 2.87 | 15.77 | 462 |
| 3 | 2 | 64 | 122 | 72 | 28.15 | 3.8 | 2.02 | 1.03 | 2.06 | 8.47 | 17.38 | 14.05 | 65.01 | 7.9 | 5.29 | 56.54 | 463 |
| 3 | 2 | 60 | 111 | 77 | 26.40 | 4.35 | 0.68 | 1.51 | 2.66 | 6.06 | 12.12 | 4.11 | 54.86 | 5 | 1.11 | 32.11 | 463 |
| 3 | 2 | 67 | 141 | 73 | 31.61 | 4.25 | 0.73 | 1.36 | 2.85 | 11 | 9.03 | 6.09 | 37.10 | 6.2 | 2.97 | 16.28 | 463 |
| 3 | 2 | 75 | 144 | 81 | 22.51 | 6.63 | 2.37 | 1.49 | 4.34 | 7.14 | 11.77 | 7.13 | 41.67 | 7.4 | 2.26 | 39.18 | 463 |
| 3 | 2 | 74 | 136 | 82 | 24.03 | 4.83 | 4.6 | 1.31 | 2.07 | 8.51 | 15.55 | 5.40 | 57.14 | 7.5 | 2.04 | 21.56 | 463 |
| 3 | 2 | 55 | 131 | 83 | 33.67 | 5.29 | 1.77 | 1.52 | 3.45 | 7.81 | 14.48 | 8.33 | 64.60 | 7 | 2.89 | 38.65 | 467 |
| 3 | 2 | 66 | 130 | 72 | 24.46 | 5.11 | 0.83 | 1.26 | 2.48 | 7.17 | 10.06 | 4.12 | 51.74 | 6.8 | 1.31 | 22.45 | 467 |
| 3 | 2 | 61 | 135 | 79 | 22.15 | 6.44 | 0.99 | 1.91 | 3.82 | 7.88 | 15.89 | 4.28 | 25.18 | 7.3 | 1.50 | 19.54 | 467 |
| 3 | 2 | 58 | 132 | 82 | 27.06 | 6.64 | 2.83 | 1.84 | 3.41 | 9.53 | 16.59 | 7.75 | 56.68 | 8.1 | 3.28 | 25.70 | 468 |
| 3 | 2 | 67 | 147 | 100 | 22.58 | 4.92 | 0.89 | 1.56 | 3.36 | 9.2 | 9.37 | 6.10 | 28.11 | 5.7 | 2.49 | 21.40 | 471 |
| 3 | 2 | 58 | 136 | 88 | 26.37 | 5.08 | 3.09 | 1.25 | 3.26 | 8.5 | 11.84 | 4.78 | 25.60 | 6.7 | 1.81 | 19.12 | 471 |
| 3 | 2 | 65 | 130 | 80 | 26.23 | 3.98 | 0.95 | 1.32 | 2.49 | 6.09 | 11.96 | 6.91 | 52.19 | 11.6 | 1.87 | 53.36 | 471 |
| 3 | 2 | 81 | 161 | 71 | 28.87 | 4.37 | 1.52 | 1.34 | 2.82 | 7.64 | 14.91 | 8.35 | 62.70 | 5.8 | 2.84 | 40.34 | 478 |
| 3 | 2 | 80 | 145 | 81 | 24.30 | 5.81 | 1.56 | 1.5 | 3.86 | 6.63 | 11.94 | 5.10 | 26.33 | 6.3 | 1.50 | 32.59 | 478 |
| 3 | 2 | 64 | 140 | 91 | 21.50 | 5.54 | 0.5 | 1.68 | 2.31 | 5.94 | 7.61 | 5.16 | 49.27 | 5.9 | 1.36 | 42.30 | 482 |
| 3 | 2 | 50 | 110 | 66 | 29.88 | 4.14 | 0.99 | 0.64 | 2.09 | 5.18 | 8.3 | 4.37 | 34.50 | 5.4 | 1.01 | 52.02 | 482 |
| 3 | 2 | 56 | 137 | 85 | 21.33 | 4.33 | 1.03 | 1.65 | 2.4 | 6.97 | 9.76 | 6.45 | 27.92 | 5.4 | 2.00 | 37.18 | 484 |
| 3 | 2 | 46 | 128 | 79 | 24.20 | 10.86 | 22.65 | 0.58 | 3.31 | 11.7 | 16.59 | 5.73 | 30.33 | 10.3 | 2.98 | 13.99 | 484 |
| 3 | 2 | 58 | 160 | 90 | 37.94 | 6.46 | 2.18 | 1.36 | 3.46 | 7.92 | 16.17 | 13.42 | 66.15 | 7.1 | 4.72 | 60.72 | 487 |
| 3 | 2 | 68 | 131 | 76 | 27.48 | 6.59 | 2.66 | 1.55 | 4.8 | 6.73 | 15.1 | 8.70 | 51.90 | 6.7 | 2.60 | 53.87 | 487 |
| 3 | 2 | 60 | 117 | 83 | 29.67 | 5.67 | 1.52 | 1.48 | 3.5 | 11.7 | 15.13 | 13.82 | 34.31 | 10.2 | 7.16 | 33.91 | 490 |
| 3 | 2 | 51 | 141 | 92 | 29.01 | 6.97 | 0.97 | 1.61 | 3.92 | 10.6 | 13.11 | 7.42 | 24.23 | 13.2 | 3.50 | 20.87 | 496 |
| 3 | 2 | 62 | 122 | 68 | 24.44 | 4.93 | 1.16 | 1.34 | 3.14 | 5.52 | 11.24 | 5.25 | 57.40 | 6.5 | 1.29 | 51.98 | 502 |
| 3 | 2 | 63 | 112 | 72 | 29.14 | 4.53 | 1.55 | 1.15 | 3.42 | 5.68 | 8.06 | 4.13 | 66.87 | 6.1 | 1.04 | 37.89 | 444 |
| 3 | 2 | 65 | 137 | 77 | 26.50 | 5.97 | 1.3 | 1.34 | 4.46 | 11.4 | 14.68 | 5.43 | 69.95 | 11.5 | 2.75 | 13.76 | 504 |
| 3 | 2 | 69 | 134 | 74 | 25.71 | 3.73 | 1.98 | 0.93 | 2.46 | 11.8 | 11.91 | 9.69 | 24.55 | 8.7 | 5.08 | 23.38 | 506 |
| 3 | 2 | 58 | 121 | 71 | 25.64 | 4.8 | 0.55 | 0.58 | 3.25 | 8.1 | 14.94 | 4.18 | 54.83 | 7 | 1.50 | 18.17 | 406 |
| 3 | 2 | 66 | 125 | 76 | 30.48 | 4.12 | 2.54 | 1.59 | 2.43 | 5.92 | 10.89 | 7.81 | 48.62 | 7 | 2.05 | 64.55 | 507 |
| 3 | 2 | 53 | 126 | 76 | 31.43 | 4.73 | 2.74 | 1.13 | 2.94 | 6.5 | 13.49 | 8.19 | 56.50 | 5.5 | 2.37 | 54.60 | 507 |
| 3 | 2 | 56 | 146 | 78 | 28.16 | 4.98 | 1.72 | 1.41 | 3.35 | 7.87 | 11.73 | 6.01 | 28.96 | 6.7 | 2.10 | 27.51 | 508 |
| 3 | 2 | 49 | 150 | 84 | 25.96 | 6.1 | 1.39 | 1.64 | 4.24 | 9.77 | 13.03 | 11.79 | 48.06 | 7.6 | 5.12 | 37.61 | 508 |
| 3 | 2 | 53 | 127 | 77 | 24.44 | 4.5 | 2.36 | 1.1 | 3.02 | 5.93 | 12.24 | 7.51 | 70.10 | 5.7 | 1.98 | 61.81 | 309 |
| 3 | 2 | 71 | 143 | 94 | 27.33 | 6.69 | 0.49 | 1.53 | 3.87 | 10.7 | 18.37 | 9.45 | 48.62 | 9.9 | 4.48 | 26.36 | 512 |
| 3 | 2 | 60 | 118 | 84 | 24.69 | 4.51 | 0.82 | 1.45 | 2.72 | 7.62 | 14.68 | 3.84 | 37.16 | 7.3 | 1.30 | 18.64 | 513 |
| 3 | 2 | 72 | 129 | 75 | 19.38 | 3.93 | 0.95 | 1.54 | 2.07 | 7.59 | 10.05 | 6.84 | 53.04 | 6.5 | 2.31 | 33.45 | 513 |
| 3 | 2 | 69 | 121 | 75 | 23.19 | 4.25 | 0.82 | 1.4 | 2.34 | 7.54 | 14.13 | 6.66 | 64.03 | 6.7 | 2.23 | 32.97 | 513 |
| 3 | 2 | 60 | 116 | 82 | 23.14 | 5.87 | 1.2 | 1.5 | 3.78 | 6.87 | 11.43 | 6.86 | 32.74 | 7 | 2.09 | 40.71 | 515 |
| 3 | 2 | 71 | 134 | 74 | 22.21 | 4.66 | 0.58 | 0.79 | 2.54 | 8.02 | 13.02 | 5.32 | 58.16 | 7.3 | 1.90 | 23.54 | 515 |
| 3 | 2 | 63 | 133 | 83 | 20.03 | 5.04 | 0.44 | 0.84 | 3.09 | 6.4 | 13.4 | 9.55 | 54.49 | 6.4 | 2.72 | 65.86 | 316 |
| 3 | 2 | 68 | 112 | 78 | 23.19 | 6.89 | 2.38 | 1.34 | 4.59 | 6.81 | 14.08 | 8.05 | 51.60 | 6.4 | 2.44 | 48.64 | 516 |
| 3 | 2 | 68 | 137 | 81 | 21.50 | 5.39 | 1.58 | 1.9 | 3.31 | 11.1 | 13.1 | 8.66 | 59.18 | 8.6 | 4.29 | 22.67 | 420 |
| 3 | 2 | 68 | 116 | 80 | 24.92 | 5.47 | 1.55 | 1.61 | 3.44 | 7.72 | 13.99 | 7.35 | 53.24 | 7.3 | 2.52 | 34.83 | 520 |
| 3 | 2 | 63 | 131 | 81 | 32.86 | 6.94 | 1.33 | 0.85 | 4.69 | 11.9 | 13.04 | 4.07 | 43.71 | 10.9 | 2.15 | 9.69 | 520 |
| 3 | 2 | 67 | 142 | 74 | 33.11 | 5.23 | 1.02 | 1.32 | 3.6 | 6 | 13.09 | 6.08 | 51.60 | 6.3 | 1.62 | 48.64 | 521 |
| 3 | 2 | 61 | 128 | 80 | 24.66 | 4.68 | 1.48 | 1.53 | 3.03 | 9.58 | 13.52 | 13.33 | 65.77 | 8.8 | 5.68 | 43.85 | 521 |
| 3 | 2 | 65 | 114 | 80 | 28.44 | 6.22 | 1.63 | 1.5 | 3.98 | 7.81 | 14.74 | 9.30 | 58.40 | 7.1 | 3.23 | 43.16 | 521 |
| 3 | 2 | 68 | 132 | 72 | 26.17 | 3.67 | 0.92 | 1.12 | 1.96 | 6.9 | 8.61 | 8.89 | 20.00 | 7 | 2.73 | 52.29 | 523 |
| 3 | 2 | 57 | 122 | 77 | 25.60 | 5.31 | 1.29 | 1.39 | 3.43 | 5.23 | 11.44 | 8.78 | 30.35 | 5.8 | 2.04 | 101.50 | 535 |
| 3 | 2 | 63 | 132 | 78 | 23.37 | 6.05 | 2.28 | 1.53 | 3.1 | 7.64 | 9.74 | 6.23 | 65.81 | 6.5 | 2.12 | 30.10 | 437 |
| 3 | 2 | 78 | 137 | 77 | 25.65 | 5.38 | 2.36 | 1.13 | 3.39 | 5.43 | 9.69 | 11.37 | 48.83 | 6.9 | 2.74 | 117.82 | 540 |
| 3 | 2 | 71 | 141 | 81 | 22.58 | 4.77 | 0.5 | 2.21 | 2.36 | 6.66 | 10.08 | 4.67 | 51.86 | 6.5 | 1.38 | 29.56 | 543 |
| 3 | 2 | 68 | 111 | 77 | 24.45 | 5.15 | 1.2 | 1.44 | 3.2 | 8.12 | 15.89 | 7.27 | 42.10 | 6.9 | 2.62 | 31.47 | 548 |
| 3 | 2 | 68 | 140 | 70 | 29.48 | 4.23 | 1.7 | 1.06 | 2.74 | 7.72 | 15.87 | 5.00 | 44.87 | 8 | 1.72 | 23.70 | 551 |
| 3 | 2 | 67 | 144 | 76 | 26.30 | 5.35 | 2.83 | 1.21 | 3.7 | 7.7 | 16.12 | 5.65 | 30.40 | 6.9 | 1.93 | 26.90 | 551 |
| 3 | 2 | 69 | 130 | 82 | 27.88 | 4.37 | 0.7 | 1.53 | 2.63 | 5.06 | 7.46 | 6.15 | 62.79 | 5.6 | 1.38 | 78.85 | 551 |
| 3 | 2 | 63 | 126 | 70 | 27.41 | 4.56 | 0.45 | 1.81 | 2.94 | 7.76 | 7.49 | 11.26 | 57.75 | 7.1 | 3.88 | 52.86 | 560 |
| 3 | 2 | 48 | 117 | 72 | 22.55 | 5.02 | 2.86 | 1.31 | 3.82 | 7.01 | 11.62 | 9.20 | 47.00 | 7.2 | 2.87 | 52.42 | 560 |
| 3 | 2 | 65 | 143 | 78 | 25.81 | 5.7 | 3.46 | 1.61 | 3 | 10.1 | 14.28 | 4.34 | 17.05 | 8.3 | 1.96 | 13.07 | 560 |
| 3 | 2 | 60 | 121 | 71 | 34.38 | 4.21 | 3.87 | 1.22 | 2.41 | 12 | 15.24 | 5.32 | 18.02 | 11.4 | 2.84 | 12.52 | 563 |
| 3 | 2 | 53 | 128 | 80 | 28.72 | 5.47 | 1.14 | 1.46 | 3.82 | 6.47 | 7.89 | 7.62 | 62.48 | 6.6 | 2.19 | 51.31 | 565 |
| 3 | 2 | 58 | 129 | 79 | 30.30 | 7.53 | 3.19 | 1.87 | 5.11 | 6.93 | 12.64 | 6.19 | 38.20 | 6.5 | 1.91 | 36.09 | 565 |
| 3 | 2 | 69 | 122 | 76 | 26.44 | 4.85 | 1.05 | 1.34 | 3.36 | 5.57 | 7.98 | 8.67 | 66.44 | 6.2 | 2.15 | 83.77 | 567 |
| 3 | 2 | 48 | 130 | 76 | 28.04 | 4.53 | 2.64 | 1.48 | 2.74 | 8.45 | 14.79 | 6.72 | 65.07 | 8.5 | 2.52 | 27.15 | 567 |
| 3 | 2 | 57 | 123 | 78 | 28.30 | 6.45 | 2.03 | 1.37 | 4.21 | 7.25 | 14.8 | 11.73 | 56.50 | 6.8 | 3.78 | 62.56 | 585 |
| 3 | 2 | 41 | 135 | 79 | 35.70 | 6.99 | 3.02 | 1.1 | 2.28 | 7.07 | 9.97 | 7.70 | 32.80 | 7 | 2.42 | 43.14 | 585 |
| 3 | 2 | 60 | 125 | 65 | 33.71 | 3.24 | 0.82 | 1.02 | 1.89 | 7.21 | 9.76 | 6.10 | 29.19 | 7.4 | 1.95 | 32.88 | 500 |
| 3 | 2 | 76 | 138 | 82 | 18.99 | 4.46 | 0.61 | 1.89 | 2.25 | 5.39 | 8.39 | 5.32 | 19.32 | 5.4 | 1.27 | 56.30 | 503 |
| 3 | 2 | 73 | 110 | 76 | 24.32 | 5.2 | 2.08 | 1.35 | 3.11 | 6.36 | 10.88 | 9.19 | 65.17 | 6.2 | 2.60 | 64.27 | 507 |
| 3 | 2 | 63 | 128 | 76 | 31.56 | 4.77 | 1.68 | 1.44 | 3.08 | 5.41 | 6.81 | 7.76 | 39.03 | 5.9 | 1.87 | 81.26 | 507 |
| 3 | 2 | 77 | 141 | 71 | 18.99 | 4.98 | 0.9 | 1.38 | 3.26 | 7.98 | 11.46 | 5.15 | 46.76 | 6.7 | 1.83 | 22.99 | 409 |
| 3 | 2 | 79 | 131 | 69 | 21.91 | 4.97 | 2.2 | 1.05 | 3.58 | 8.58 | 15.63 | 8.93 | 57.20 | 7.6 | 3.41 | 35.16 | 612 |
| 3 | 2 | 78 | 135 | 75 | 31.16 | 4.83 | 1.35 | 1.25 | 3 | 9.35 | 15.23 | 7.89 | 33.60 | 7.9 | 3.28 | 26.97 | 515 |
| 3 | 2 | 78 | 140 | 88 | 23.50 | 4.86 | 0.82 | 2.18 | 2.4 | 6.32 | 6.97 | 7.36 | 62.39 | 5.6 | 2.07 | 52.20 | 415 |
| 3 | 2 | 62 | 122 | 68 | 29.22 | 3.98 | 1.71 | 1.32 | 2.59 | 6.25 | 9.26 | 14.80 | 36.52 | 6.1 | 4.11 | 107.64 | 415 |
| 3 | 2 | 74 | 135 | 81 | 23.44 | 4.39 | 1.15 | 1.35 | 2.64 | 6.65 | 14.6 | 7.11 | 61.13 | 5.7 | 2.10 | 45.14 | 543 |
| 3 | 2 | 65 | 146 | 99 | 26.29 | 6.19 | 1.15 | 1.56 | 4.1 | 7.08 | 14.04 | 6.04 | 39.66 | 7.4 | 1.90 | 33.74 | 578 |
| 3 | 2 | 60 | 122 | 72 | 28.40 | 4.67 | 1.85 | 1.19 | 2.9 | 11.5 | 13.37 | 7.52 | 50.50 | 11.2 | 3.84 | 18.82 | 440 |

Group: 1 Control 2 pre-diabetes 3 T2DM

Gender: 1 male 2 female

**Abbreviations：**UA, uric acid; BMI, body mass index; DBP, diastolic blood pressure; FINS, fasting insulin; FPG, fasting plasma glucose; HbA1c, glycosylated hemoglobin; HDL, high-density lipoprotein; HOMA-IR/β, homeostasis model assessment-insulin resistance/β-cell function; LDL, low-density lipoprotein; PBG, postprandial blood glucose; Pre-DM, prediabetes; SBP, systolic blood pressure; T2DM, type 2 diabetes mellitus; TC, total cholesterol; TG, triglycerides.
