# Supplementary material for: SLC2A9 rs1014290 Polymorphism is Associated with Prediabetes and Type 2 Diabetes
Source: Int J Endocrinol. 2022 Dec 12;2022:4947684. doi: 10.1155/2022/4947684 (PMC9763018; doi:10.1155/2022/4947684)
Supplement: Supplementary Materials — Original data-1. Collect general information such as age, gender, body mass index, and blood pressure. We use an automatic biochemical analyzer to detect blood glucose at each time of the glucose tolerance test, uric acid (UA), and blood lipids (including TC, TG, LDL, and HDL) (Hitachi 7600-110, Japan). Detection of glycated hemoglobin (HbA1c) by chromatographic analysis (ADAMS A1c HA-8180 analyzer, Japan). Determination of serum insulin levels by electrochemiluminescence (Roche Cobas e601, Germany). The degree of insulin resistance was assessed by calculating beta cell function (HOMA-β) and the insulin resistance index (HOMA-IR), using the HOMA2 calculator version 2.2.3 (Oxford University Diabetes Trial Unit). Original data-2. Genotyping and determination of gene polymorphisms were carried out with reference to the literature [20]. Genomic DNA extraction kit (Generay Biotechnology, Shanghai, China). Genotyping of patient SLC2A9 by DNA sequencing (ABI3730 genetic analyzer, Applied Biosystems, USA). The primers for gene amplification were as follows: 5'-GGATTCACAACTATCTTACTCAT-3' (forward); 5'-CAGGGTTATGTTCCATTTATCT-3' (reverse); designed using Primer Premier 5.0 (Premier Biosoft International, CA, USA). The samples were sequenced using GeneMarker v2.2.0 (PA, USA). [file 4947684.f1.zip › original data-2.docx]

Concise supplementary material description:

Genotyping and determination of gene polymorphisms were carried out with reference to the literature^[20]^. Genomic DNA extraction kit (Generay Biotechnology, Shanghai, China). Genotyping of patient SLC2A9 by DNA sequencing (ABI3730 genetic analyzer, Applied Biosystems, USA). The primers for gene amplification were as follows: 5′-GGATTCACAACTATCTTACTCAT-3′ (forward); 5′-CAGGGTTATGTTCCATTTATCT-3′ (reverse); designed using Primer Premier 5.0 (Premier Biosoft International, CA, USA). The samples were sequenced using GeneMarker v2.2.0 (PA, USA).

| **con** | **S1** | **pre-DM** | **S1** | **DM** | **S1** |
| --- | --- | --- | --- | --- | --- |
|  | **rs1014290** |  | **rs1014290** |  | **rs1014290** |
|  | **A/G** |  | **A/G** |  | **A/G** |
| 1 | A A | 349 | G G | 707 | G G |
| 2 | A A | 350 | G G | 708 | G G |
| 3 | A A | 351 | G G | 709 | G G |
| 4 | A A | 352 | G G | 710 | G G |
| 5 | A A | 353 | G G | 711 | G G |
| 6 | A A | 354 | G G | 712 | G G |
| 7 | A A | 355 | G G | 713 | G G |
| 8 | A A | 356 | G G | 714 | G G |
| 9 | A A | 357 | G G | 715 | G G |
| 10 | A A | 358 | G G | 716 | G G |
| 11 | A A | 359 | G G | 717 | G G |
| 12 | A A | 360 | G G | 718 | G G |
| 13 | A A | 361 | G G | 719 | G G |
| 14 | A A | 362 | G G | 720 | G G |
| 15 | A A | 363 | G G | 721 | G G |
| 16 | A A | 364 | G G | 722 | G G |
| 17 | A A | 365 | G G | 723 | G G |
| 18 | A A | 366 | G G | 724 | G G |
| 19 | A A | 367 | G G | 725 | G G |
| 20 | A A | 368 | G G | 726 | G G |
| 21 | A A | 369 | G G | 727 | G G |
| 22 | A A | 370 | G G | 728 | G G |
| 23 | A A | 371 | G G | 729 | G G |
| 24 | A A | 372 | G G | 730 | G G |
| 25 | A A | 373 | G G | 731 | G G |
| 26 | A A | 374 | G G | 732 | G G |
| 27 | A A | 375 | G G | 733 | G G |
| 28 | A A | 376 | G G | 734 | G G |
| 29 | A A | 377 | G G | 735 | G G |
| 30 | A A | 378 | G G | 736 | G G |
| 31 | A A | 379 | G G | 737 | G G |
| 32 | A A | 380 | G G | 738 | G G |
| 33 | A A | 381 | G G | 739 | G G |
| 34 | A A | 382 | G G | 740 | G G |
| 35 | A A | 383 | G G | 741 | G G |
| 36 | A A | 384 | G G | 742 | G G |
| 37 | A A | 385 | G G | 743 | G G |
| 38 | A A | 386 | G G | 744 | G G |
| 39 | A A | 387 | G G | 745 | G G |
| 40 | A A | 388 | G G | 746 | G G |
| 41 | A A | 389 | G G | 747 | G G |
| 42 | A A | 390 | G G | 748 | G G |
| 43 | A A | 391 | G G | 749 | G G |
| 44 | A A | 392 | G G | 750 | G G |
| 45 | A A | 393 | G G | 751 | G G |
| 46 | A A | 394 | G G | 752 | G G |
| 47 | A A | 395 | G G | 753 | G G |
| 48 | A A | 396 | G G | 754 | G G |
| 49 | A A | 397 | G G | 755 | G G |
| 50 | A A | 398 | G G | 756 | G G |
| 51 | A A | 399 | G G | 757 | G G |
| 52 | A A | 400 | G G | 758 | G G |
| 53 | A A | 401 | G G | 759 | A A |
| 54 | A A | 402 | G G | 760 | A A |
| 55 | A A | 403 | G G | 761 | A A |
| 56 | A A | 404 | G G | 762 | A A |
| 57 | A A | 405 | G G | 763 | A A |
| 58 | A A | 406 | G G | 764 | A A |
| 59 | A A | 407 | G G | 765 | A A |
| 60 | A A | 408 | G G | 766 | A A |
| 61 | A A | 409 | G G | 767 | A A |
| 62 | A A | 410 | G G | 768 | A A |
| 63 | A A | 411 | G G | 769 | A A |
| 64 | A A | 412 | A A | 770 | A A |
| 65 | A A | 413 | A A | 771 | A A |
| 66 | A A | 414 | A A | 772 | A A |
| 67 | A A | 415 | A A | 773 | A A |
| 68 | A A | 416 | A A | 774 | A A |
| 69 | A A | 417 | A A | 775 | A A |
| 70 | A A | 418 | A A | 776 | A A |
| 71 | A A | 419 | A A | 777 | A A |
| 72 | A A | 420 | A A | 778 | A A |
| 73 | A A | 421 | A A | 779 | A A |
| 74 | A A | 422 | A A | 780 | A A |
| 75 | A A | 423 | A A | 781 | A A |
| 76 | A A | 424 | A A | 782 | A A |
| 77 | A A | 425 | A A | 783 | A A |
| 78 | A A | 426 | A A | 784 | A A |
| 79 | A A | 427 | A A | 785 | A A |
| 80 | A A | 428 | A A | 786 | A A |
| 81 | A A | 429 | A A | 787 | A A |
| 82 | A A | 430 | A A | 788 | A A |
| 83 | A A | 431 | A A | 789 | A A |
| 84 | A A | 432 | A A | 790 | A A |
| 85 | A A | 433 | A A | 791 | A A |
| 86 | A A | 434 | A A | 792 | A A |
| 87 | A A | 435 | A A | 793 | A A |
| 88 | A A | 436 | A A | 794 | A A |
| 89 | A A | 437 | A A | 795 | A A |
| 90 | A A | 438 | A A | 796 | A A |
| 91 | A A | 439 | A A | 797 | A A |
| 92 | A A | 440 | A A | 798 | A A |
| 93 | A A | 441 | A A | 799 | A A |
| 94 | A A | 442 | A A | 800 | A A |
| 95 | A A | 443 | A A | 801 | A A |
| 96 | A A | 444 | A A | 802 | A A |
| 97 | A A | 445 | A A | 803 | A A |
| 98 | A A | 446 | A A | 804 | A A |
| 99 | A A | 447 | A A | 805 | A A |
| 100 | A A | 448 | A A | 806 | A A |
| 101 | A A | 449 | A A | 807 | A A |
| 102 | A A | 450 | A A | 808 | A A |
| 103 | A A | 451 | A A | 809 | A A |
| 104 | A A | 452 | A A | 810 | A A |
| 105 | A G | 453 | A A | 811 | A A |
| 106 | A G | 454 | A A | 812 | A A |
| 107 | A G | 455 | A A | 813 | A A |
| 108 | A G | 456 | A A | 814 | A A |
| 109 | A G | 457 | A A | 815 | A A |
| 110 | A G | 458 | A A | 816 | A A |
| 111 | A G | 459 | A A | 817 | A A |
| 112 | A G | 460 | A A | 818 | A A |
| 113 | A G | 461 | A A | 819 | A A |
| 114 | A G | 462 | A A | 820 | A A |
| 115 | A G | 463 | A A | 821 | A A |
| 116 | A G | 464 | A A | 822 | A A |
| 117 | A G | 465 | A A | 823 | A A |
| 118 | A G | 466 | A A | 824 | A A |
| 119 | A G | 467 | A A | 825 | A A |
| 120 | A G | 468 | A A | 826 | A A |
| 121 | A G | 469 | A A | 827 | A A |
| 122 | A G | 470 | A A | 828 | A A |
| 123 | A G | 471 | A A | 829 | A A |
| 124 | A G | 472 | A A | 830 | A A |
| 125 | A G | 473 | A A | 831 | A A |
| 126 | A G | 474 | A A | 832 | A A |
| 127 | A G | 475 | A A | 833 | A A |
| 128 | A G | 476 | A A | 834 | A A |
| 129 | A G | 477 | A A | 835 | A A |
| 130 | A G | 478 | A A | 836 | A A |
| 131 | A G | 479 | A A | 837 | A A |
| 132 | A G | 480 | A A | 838 | A A |
| 133 | A G | 481 | A A | 839 | A A |
| 134 | A G | 482 | A A | 840 | A A |
| 135 | A G | 483 | A A | 841 | A A |
| 136 | A G | 484 | A A | 842 | A A |
| 137 | A G | 485 | A A | 843 | A A |
| 138 | A G | 486 | A A | 844 | A A |
| 139 | A G | 487 | A A | 845 | A A |
| 140 | A G | 488 | A A | 846 | A A |
| 141 | A G | 489 | A A | 847 | A A |
| 142 | A G | 490 | A A | 848 | A A |
| 143 | A G | 491 | A A | 849 | A A |
| 144 | A G | 492 | A A | 850 | A A |
| 145 | A G | 493 | A A | 851 | A A |
| 146 | A G | 494 | A A | 852 | A A |
| 147 | A G | 495 | A A | 853 | A A |
| 148 | A G | 496 | A A | 854 | A A |
| 149 | A G | 497 | A A | 855 | A A |
| 150 | A G | 498 | A A | 856 | A A |
| 151 | A G | 499 | A A | 857 | A A |
| 152 | A G | 500 | A A | 858 | A A |
| 153 | A G | 501 | A A | 859 | A A |
| 154 | A G | 502 | A A | 860 | A A |
| 155 | A G | 503 | A A | 861 | A A |
| 156 | A G | 504 | A A | 862 | A A |
| 157 | A G | 505 | A A | 863 | A A |
| 158 | A G | 506 | A A | 864 | A A |
| 159 | A G | 507 | A A | 865 | A A |
| 160 | A G | 508 | A A | 866 | A A |
| 161 | A G | 509 | A A | 867 | A A |
| 162 | A G | 510 | A A | 868 | A A |
| 163 | A G | 511 | A A | 869 | A A |
| 164 | A G | 512 | A A | 870 | A A |
| 165 | A G | 513 | A A | 871 | A A |
| 166 | A G | 514 | A A | 872 | A A |
| 167 | A G | 515 | A A | 873 | A A |
| 168 | A G | 516 | A A | 874 | A A |
| 169 | A G | 517 | A A | 875 | A A |
| 170 | A G | 518 | A A | 876 | A A |
| 171 | A G | 519 | A A | 877 | A A |
| 172 | A G | 520 | A A | 878 | A A |
| 173 | A G | 521 | A A | 879 | A A |
| 174 | A G | 522 | A A | 880 | A A |
| 175 | A G | 523 | A A | 881 | A A |
| 176 | A G | 524 | A A | 882 | A A |
| 177 | A G | 525 | A A | 883 | A A |
| 178 | A G | 526 | A A | 884 | A A |
| 179 | A G | 527 | A G | 885 | A A |
| 180 | A G | 528 | A G | 886 | A A |
| 181 | A G | 529 | A G | 887 | A G |
| 182 | A G | 530 | A G | 888 | A G |
| 183 | A G | 531 | A G | 889 | A G |
| 184 | A G | 532 | A G | 890 | A G |
| 185 | A G | 533 | A G | 891 | A G |
| 186 | A G | 534 | A G | 892 | A G |
| 187 | A G | 535 | A G | 893 | A G |
| 188 | A G | 536 | A G | 894 | A G |
| 189 | A G | 537 | A G | 895 | A G |
| 190 | A G | 538 | A G | 896 | A G |
| 191 | A G | 539 | A G | 897 | A G |
| 192 | A G | 540 | A G | 898 | A G |
| 193 | A G | 541 | A G | 899 | A G |
| 194 | A G | 542 | A G | 900 | A G |
| 195 | A G | 543 | A G | 901 | A G |
| 196 | A G | 544 | A G | 902 | A G |
| 197 | A G | 545 | A G | 903 | A G |
| 198 | A G | 546 | A G | 904 | A G |
| 199 | A G | 547 | A G | 905 | A G |
| 200 | A G | 548 | A G | 906 | A G |
| 201 | A G | 549 | A G | 907 | A G |
| 202 | A G | 550 | A G | 908 | A G |
| 203 | A G | 551 | A G | 909 | A G |
| 204 | A G | 552 | A G | 910 | A G |
| 205 | A G | 553 | A G | 911 | A G |
| 206 | A G | 554 | A G | 912 | A G |
| 207 | A G | 555 | A G | 913 | A G |
| 208 | A G | 556 | A G | 914 | A G |
| 209 | A G | 557 | A G | 915 | A G |
| 210 | A G | 558 | A G | 916 | A G |
| 211 | A G | 559 | A G | 917 | A G |
| 212 | A G | 560 | A G | 918 | A G |
| 213 | A G | 561 | A G | 919 | A G |
| 214 | A G | 562 | A G | 920 | A G |
| 215 | A G | 563 | A G | 921 | A G |
| 216 | A G | 564 | A G | 922 | A G |
| 217 | A G | 565 | A G | 923 | A G |
| 218 | A G | 566 | A G | 924 | A G |
| 219 | A G | 567 | A G | 925 | A G |
| 220 | A G | 568 | A G | 926 | A G |
| 221 | A G | 569 | A G | 927 | A G |
| 222 | A G | 570 | A G | 928 | A G |
| 223 | A G | 571 | A G | 929 | A G |
| 224 | A G | 572 | A G | 930 | A G |
| 225 | A G | 573 | A G | 931 | A G |
| 226 | A G | 574 | A G | 932 | A G |
| 227 | A G | 575 | A G | 933 | A G |
| 228 | A G | 576 | A G | 934 | A G |
| 229 | A G | 577 | A G | 935 | A G |
| 230 | A G | 578 | A G | 936 | A G |
| 231 | A G | 579 | A G | 937 | A G |
| 232 | A G | 580 | A G | 938 | A G |
| 233 | A G | 581 | A G | 939 | A G |
| 234 | A G | 582 | A G | 940 | A G |
| 235 | A G | 583 | A G | 941 | A G |
| 236 | A G | 584 | A G | 942 | A G |
| 237 | A G | 585 | A G | 943 | A G |
| 238 | A G | 586 | A G | 944 | A G |
| 239 | A G | 587 | A G | 945 | A G |
| 240 | A G | 588 | A G | 946 | A G |
| 241 | A G | 589 | A G | 947 | A G |
| 242 | A G | 590 | A G | 948 | A G |
| 243 | A G | 591 | A G | 949 | A G |
| 244 | A G | 592 | A G | 950 | A G |
| 245 | A G | 593 | A G | 951 | A G |
| 246 | A G | 594 | A G | 952 | A G |
| 247 | A G | 595 | A G | 953 | A G |
| 248 | A G | 596 | A G | 954 | A G |
| 249 | A G | 597 | A G | 955 | A G |
| 250 | A G | 598 | A G | 956 | A G |
| 251 | A G | 599 | A G | 957 | A G |
| 252 | A G | 600 | A G | 958 | A G |
| 253 | A G | 601 | A G | 959 | A G |
| 254 | A G | 602 | A G | 960 | A G |
| 255 | A G | 603 | A G | 961 | A G |
| 256 | A G | 604 | A G | 962 | A G |
| 257 | A G | 605 | A G | 963 | A G |
| 258 | A G | 606 | A G | 964 | A G |
| 259 | A G | 607 | A G | 965 | A G |
| 260 | A G | 608 | A G | 966 | A G |
| 261 | A G | 609 | A G | 967 | A G |
| 262 | A G | 610 | A G | 968 | A G |
| 263 | A G | 611 | A G | 969 | A G |
| 264 | A G | 612 | A G | 970 | A G |
| 265 | A G | 613 | A G | 971 | A G |
| 266 | A G | 614 | A G | 972 | A G |
| 267 | A G | 615 | A G | 973 | A G |
| 268 | A G | 616 | A G | 974 | A G |
| 269 | A G | 617 | A G | 975 | A G |
| 270 | A G | 618 | A G | 976 | A G |
| 271 | A G | 619 | A G | 977 | A G |
| 272 | A G | 620 | A G | 978 | A G |
| 273 | A G | 621 | A G | 979 | A G |
| 274 | A G | 622 | A G | 980 | A G |
| 275 | A G | 623 | A G | 981 | A G |
| 276 | A G | 624 | A G | 982 | A G |
| 277 | A G | 625 | A G | 983 | A G |
| 278 | G G | 626 | A G | 984 | A G |
| 279 | G G | 627 | A G | 985 | A G |
| 280 | G G | 628 | A G | 986 | A G |
| 281 | G G | 629 | A G | 987 | A G |
| 282 | G G | 630 | A G | 988 | A G |
| 283 | G G | 631 | A G | 989 | A G |
| 284 | G G | 632 | A G | 990 | A G |
| 285 | G G | 633 | A G | 991 | A G |
| 286 | G G | 634 | A G | 992 | A G |
| 287 | G G | 635 | A G | 993 | A G |
| 288 | G G | 636 | A G | 994 | A G |
| 289 | G G | 637 | A G | 995 | A G |
| 290 | G G | 638 | A G | 996 | A G |
| 291 | G G | 639 | A G | 997 | A G |
| 292 | G G | 640 | A G | 998 | A G |
| 293 | G G | 641 | A G | 999 | A G |
| 294 | G G | 642 | A G | 1000 | A G |
| 295 | G G | 643 | A G | 1001 | A G |
| 296 | G G | 644 | A G | 1002 | A G |
| 297 | G G | 645 | A G | 1003 | A G |
| 298 | G G | 646 | A G | 1004 | A G |
| 299 | G G | 647 | A G | 1005 | A G |
| 300 | G G | 648 | A G | 1006 | A G |
| 301 | G G | 649 | A G | 1007 | A G |
| 302 | G G | 650 | A G | 1008 | A G |
| 303 | G G | 651 | A G | 1009 | A G |
| 304 | G G | 652 | A G | 1010 | A G |
| 305 | G G | 653 | A G | 1011 | A G |
| 306 | G G | 654 | A G | 1012 | A G |
| 307 | G G | 655 | A G | 1013 | A G |
| 308 | G G | 656 | A G | 1014 | A G |
| 309 | G G | 657 | A G | 1015 | A G |
| 310 | G G | 658 | A G | 1016 | A G |
| 311 | G G | 659 | A G | 1017 | A G |
| 312 | G G | 660 | A G | 1018 | A G |
| 313 | G G | 661 | A G | 1019 | A G |
| 314 | G G | 662 | A G | 1020 | A G |
| 315 | G G | 663 | A G | 1021 | A G |
| 316 | G G | 664 | A G | 1022 | A G |
| 317 | G G | 665 | A G | 1023 | A G |
| 318 | G G | 666 | A G | 1024 | A G |
| 319 | G G | 667 | A G | 1025 | A G |
| 320 | G G | 668 | A G | 1026 | A G |
| 321 | G G | 669 | A G | 1027 | A G |
| 322 | G G | 670 | A G | 1028 | A G |
| 323 | G G | 671 | A G | 1029 | A G |
| 324 | G G | 672 | A G | 1030 | A G |
| 325 | G G | 673 | A G | 1031 | A G |
| 326 | G G | 674 | A G | 1032 | A G |
| 327 | G G | 675 | A G | 1033 | A G |
| 328 | G G | 676 | A G | 1034 | A G |
| 329 | G G | 677 | A G | 1035 | A G |
| 330 | G G | 678 | A G | 1036 | A G |
| 331 | G G | 679 | A G | 1037 | A G |
| 332 | G G | 680 | A G | 1038 | A G |
| 333 | G G | 681 | A G | 1039 | A G |
| 334 | G G | 682 | A G | 1040 | A G |
| 335 | G G | 683 | A G | 1041 | A G |
| 336 | G G | 684 | A G | 1042 | A G |
| 337 | G G | 685 | A G | 1043 | A G |
| 338 | G G | 686 | A G | 1044 | A G |
| 339 | G G | 687 | A G | 1045 | A G |
| 340 | G G | 688 | A G | 1046 | A G |
| 341 | G G | 689 | A G | 1047 | A G |
| 342 | G G | 690 | A G | 1048 | A G |
| 343 | G G | 691 | A G | 1049 | A G |
| 344 | G G | 692 | A G | 1050 | A G |
| 345 | G G | 693 | A G | 1051 | A G |
| 346 | G G | 694 | A G | 1052 | A G |
| 347 | G G | 695 | A G | 1053 | A G |
| 348 | G G | 696 | A G | 1054 | A G |
|  |  | 697 | A G | 1055 | A G |
|  |  | 698 | A G | 1056 | A G |
|  |  | 699 | A G | 1057 | A G |
|  |  | 700 | A G | 1058 | A G |
|  |  | 701 | A G |  |  |
|  |  | 702 | A G |  |  |
|  |  | 703 | A G |  |  |
|  |  | 704 | A G |  |  |
|  |  | 705 | A G |  |  |
|  |  | 706 | A G |  |  |
